# A mild and atom-efficient four-component cascade strategy for the construction of biologically relevant 4-hydroxyquinolin-2(1*H*)-one derivatives

**DOI:** 10.3762/bjoc.22.18

**Published:** 2026-02-09

**Authors:** Dmitrii A Grishin, Kseniia I Sharkovskaia, Ilya G Kolmakov, Daria A Ipatova, Rostislav A Petrov, Nikolai D Dagaev, Dmitry A Skvortsov, Maria G Khrenova, Valeriy V Andreychev, Sergei A Evteev, Yan A Ivanenkov, Roman L Antipin, Olga А Dontsova, Elena K Beloglazkina

**Affiliations:** 1 Department of Chemistry, M.V. Lomonosov Moscow State University, 119991 Moscow, Russian Federation; 2 P. Hertsen Moscow Oncology Research Institute, 2nd Botkinsky proezd, 3, 125284, Moscow, Russian Federation

**Keywords:** antibacterial activity, 4-hydroxyquinolin-2(1*H*)-one, ʟ-proline catalysis, Meldrum’s acid, Michael addition, multicomponent reaction

## Abstract

A mild and atom-economical four-component cascade reaction has been developed, enabling the efficient and selective synthesis of previously inaccessible 4-hydroxyquinolin-2(1*H*)-one derivatives. Utilizing readily available 6-halo-4-hydroxyquinolinones, aromatic aldehydes, Meldrum’s acid, and alcohols under ʟ-proline catalysis, the reaction proceeds via in situ formation of arylidene-substituted Meldrum acids followed by sequential Michael-type addition and subsequent cascade transformations. This versatile one-pot protocol delivers structurally diverse open-chain 3-arylpropanoate esters in moderate to good yields (46–69%), while cyclic pyranoquinolinones are formed under kinetically controlled conditions. Subsequent transformations afford isopropyl and cyclohexyl analogues via hydrolysis–esterification. A preliminary biological evaluation revealed low cytotoxicity and modest antibacterial activity against *Escherichia coli* ΔtolC strains. This sustainable synthetic approach constitutes the first direct access to scarcely explored open-chain quinolinone esters, expanding the medicinal chemistry toolbox with promising scaffolds for drug discovery.

## Introduction

Contemporary organic chemistry focuses on the development of universal, efficient, and environmentally benign synthetic methods that address the challenges of constructing complex molecular architectures [1–3]. Among these, multicomponent reactions (MCRs) have emerged as powerful tools, enabling the one-pot combination of three or more reactants to form multiple bonds in a single step [4–8]. This approach not only facilitates the rapid assembly of structurally complex and functionally diverse molecules, but also aligns with the principles of green and sustainable chemistry, offering high atom economy, operational simplicity, and remarkable versatility [9–13].

Owing to their flexibility in combining diverse building blocks, MCRs have found widespread application in the synthesis of biologically active compounds. This versatility significantly shortens synthetic cycles and enables the parallel exploration of diverse chemical space. The impact of MCRs is well documented in medicinal chemistry and pharmaceutical research, including the development of several approved drugs [14–18].

**Figure 1:**
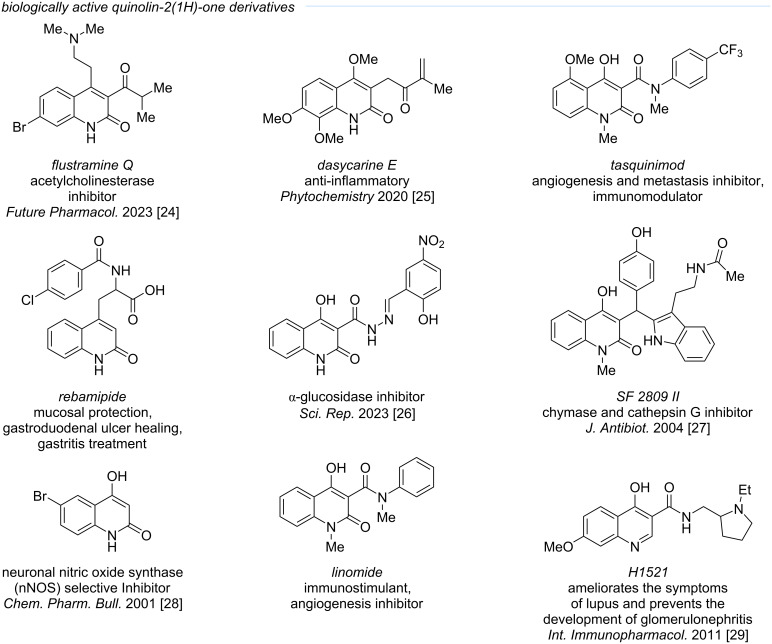
Examples of biologically active quinolin-2(1*H*)-ones.

Recently, 4-hydroxyquinoline-2(1*H*)-ones and their derivatives have attracted considerable attention due to their broad spectrum of biological activities (Figure 1) [19–29]. These include potent antibacterial, antiviral, antifungal, anti-inflammatory, and neurotropic effects, making them promising scaffolds for drug development [29–32]. Notably, many derivatives display pronounced antimicrobial properties, with several members of this class exhibiting inhibitory activity against *Mycobacterium tuberculosis*, thus representing potential candidates for antituberculosis therapy [33]. Their efficacy has also been demonstrated against *Escherichia coli*, *Staphylococcus aureus*, and *Pseudomonas aeruginosa* [34]. The diversity of pharmacological activities renders 4-hydroxyquinoline-2(1*H*)-one derivatives highly valuable targets in medicinal chemistry, driving continued interest in their synthesis and exploration [23]. However, the synthetic accessibility of structurally diversified 4-hydroxyquinolinone derivatives remains a key limitation for systematic biological exploration.

Despite the broad utility of MCRs in medicinal chemistry, no general and operationally simple method has been reported for the modular assembly of 4-hydroxyquinoline scaffolds bearing both C6-halogen and γ-(3,4-dimethoxyphenyl)propanoic acid motifs. Existing approaches to such derivatives comprise typically multiple steps, are limited in scope, or require harsh reaction conditions, restricting their application in rapid analogue generation. The combination of these pharmacophores within a single framework has not yet been systematically explored, presenting an opportunity to evaluate potential cooperative effects on antibacterial activity.

**Figure 2:**
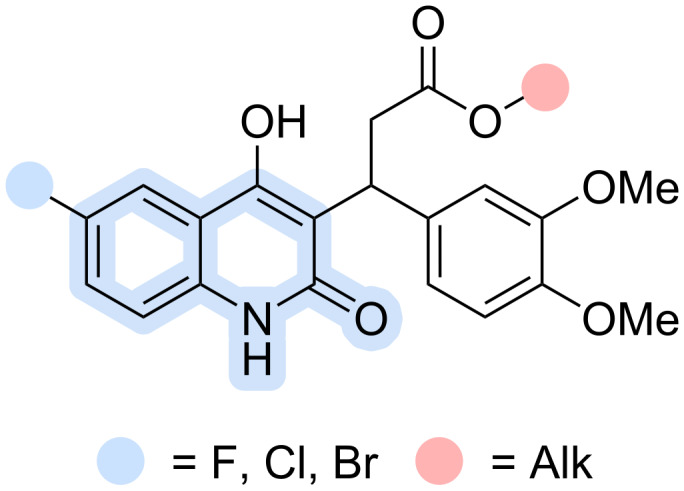
Structures obtained via rational design aimed at enhancing antibacterial activity.

In our preliminary studies, we employed a rational design strategy to identify a promising new chemotype based on the 4-hydroxyquinoline core – 3-(6-halo-4-hydroxy-2-oxo-1,2-dihydroquinolin-3-yl)-3-(3,4-dimethoxyphenyl)propanoates (Figure 2). The C6 position of the quinoline ring was halogenated with fluorine, chlorine, or bromine, as halogenation at this site is known to influence both the physicochemical properties and biological activity [33]. Halogen substituents in this position have been shown to modulate antibacterial, antiviral, and antiproliferative activities, likely through electronic effects and improved target binding. The C3 position was functionalized with a propanoic acid moiety, explored in both its open-chain (acid) and cyclized (lactone) forms [35]. The γ-position of the propanoic acid side chain was further modified with a 3,4-dimethoxyphenyl group, inspired by earlier reports [36]. The carboxylic acid functionality was either retained or converted into methyl, ethyl, isopropyl, or cyclohexyl esters to evaluate the influence of esterification on biological activity. Preliminary results indicated that compounds of this chemotype may possess antibacterial potential, forming the basis for the present study on their synthesis and biological evaluation.

**Scheme 1:**
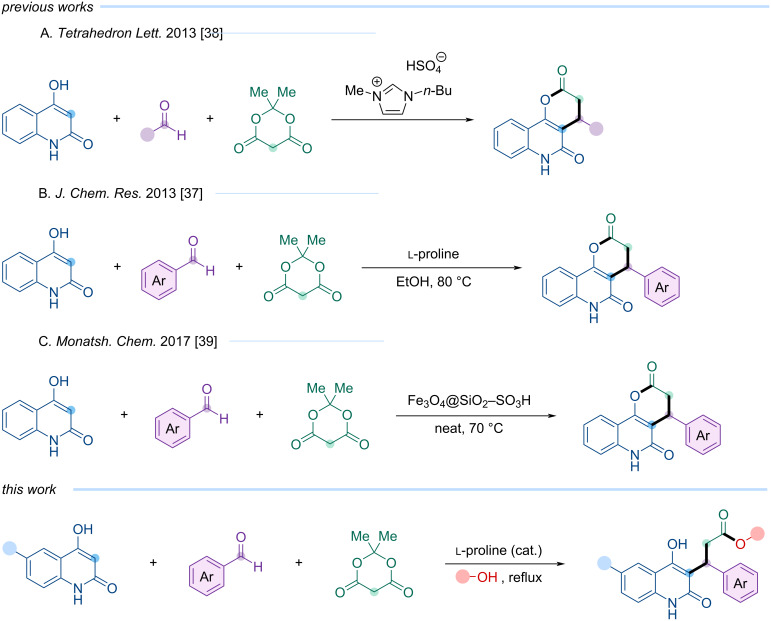
Previously reported and newly developed 3-(4-hydroxy-2-oxo-1,2-dihydroquinolin-3-yl)-3-arylpropanoic acid derivatives.

A literature survey revealed that derivatives of 3-(4-hydroxy-2-oxo-1,2-dihydroquinolin-3-yl)-3-phenylpropanoic acid have not, to the best of our knowledge, been previously described. Structurally related compounds, such as 4-aryl-4,6-dihydro-2*H*-pyrano[3,2-*c*]quinoline-2,5(3*H*)-diones, have been reported only in a few cases, typically via multicomponent reactions catalyzed by diverse agents (Scheme 1). For instance, in 2013, Shi et al. described a three-component reaction involving 4-hydroxyquinolin-2(1*H*)-one, an aromatic aldehyde, and Meldrum’s acid, catalyzed by ʟ-proline under heating [37]. In the same year, Kurosh Rad-Moghadam and co-workers developed a related protocol operating at room temperature using the ionic liquid [Bmim]HSO_4_ as a catalyst, expanding the scope to include aliphatic aldehydes [38]. Later, in 2017, Esmayeel Abbaspour-Gilandeh et al. reported a modified variant of this transformation under solvent-free heating, catalyzed by Fe_3_O_4_@SiO_2_–SO_3_H nanoparticles [39].

However, all previously reported multicomponent protocols inevitably terminate at the formation of the cyclic pyranoquinolinone scaffold, and no controlled access to open-chain quinolinone esters has been demonstrated so far.

Herein, building upon previous studies and our preliminary findings, we report an efficient four-component strategy for the synthesis of previously inaccessible 4-hydroxyquinoline-2(1*H*)-one derivatives of the type shown in Figure 2, together with an initial evaluation of their antibacterial and cytotoxic properties. This strategy delivers rapid access to these previously unreported compounds, offering a versatile platform for systematic structure–activity relationship exploration and paving the way for the development of next-generation antibacterial agents with optimized potency and selectivity.

## Results and Discussion

### Chemistry

**Scheme 2:**
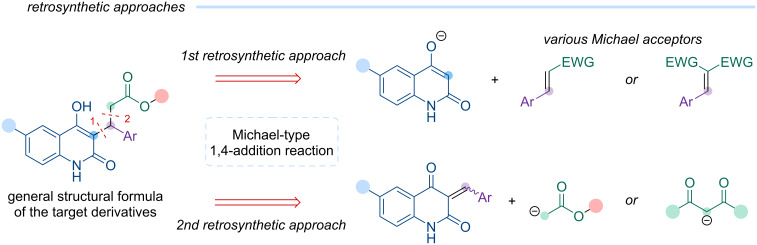
Retrosynthetic analysis: two alternative approaches to target compounds.

For developing a synthetic strategy for esters of 3-(4-hydroxy-2-oxo-1,2-dihydroquinolin-3-yl)-3-arylpropanoic acids, we initially performed a retrosynthetic analysis of the target chemotype (Scheme 2) and proposed two plausible synthetic routes, identifying a Michael 1,4-addition as the key step. The first approach involves the 1,4-Michael addition of the ambident anion derived from 6-halo-4-hydroxyquinolin-2-one to suitably activated unsaturated compounds. Considering the target structures (Figure 2), suitable Michael acceptors include α,β-unsaturated esters, aldehydes, methyl ketones, arylidene malonates, cyanoacrylates, and others. The second approach consists of the Michael 1,4-addition of the corresponding acetate or malonate anion to 3-arylidenequinolinedione. Both strategies were evaluated in subsequent studies.

**Scheme 3:**
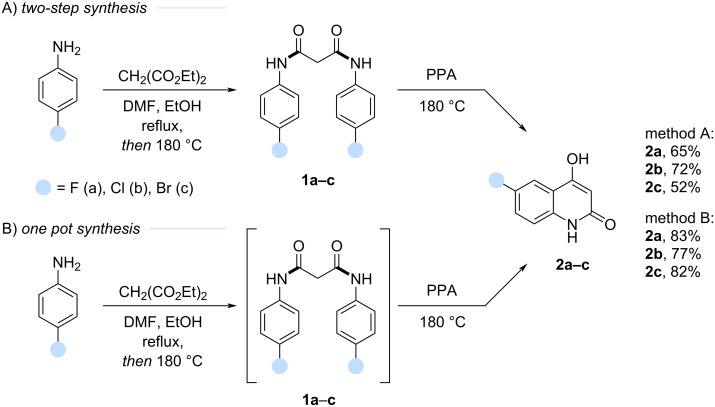
Two-stage synthesis A) and one-stage one-pot synthesis B) of 6-halogen-4-hydroxyquinoline-2(1*H*)-ones **2a**–**c**.

To implement these routes, 6-halogen-4-hydroxyquinoline-2-ones **2a**–**c** were prepared. The synthesis (Scheme 3) involved the optimization of a two-step procedure reported previously for their preparation from corresponding anilines and malonic ester [40–43].

The intermediate *N*^1^,*N*^3^-bis(4-halogenophenyl)malonamides **1a**–**c** were synthesized from 4-halogen-substituted anilines and diethyl malonate in the presence of catalytic DMF in ethanol (Scheme 3a) [40]. A small amount of ethanol ensured homogeneity of the reaction mixture, allowed gradual temperature increase, and prevented sudden charring. DMF, with its high boiling point, acted as a homogenizer by preventing solidification after ethanol evaporation. While the literature procedure required ca. 8 hours [40], we optimized it to afford compounds **1a**–**c** in approximately 4 hours with comparable yields (52–72%). Subsequent treatment of malonamides **1a**–**c** with polyphosphoric acid (PPA) at 180 °C yielded 6-halogen-4-hydroxyquinoline-2(1*H*)-ones **2a**–**c** quantitatively.

Although the two-step sequence generally performed well, isolation of malonamides **1a**–**c** was associated with significant material loss, impacting the overall yield. To address this, a one-pot synthesis directly from anilines was developed, avoiding isolation of intermediates. After approximately 4 hours, the reaction mixture solidified, indicating malonamide formation which was followed by the addition of PPA and continuation of the reaction to afford products **2a**–**c** in improved yields (77–83%). The resulting products showed negligible impurities as judged by ^1^H NMR and were identical to those obtained via the two-step procedure.

**Scheme 4:**
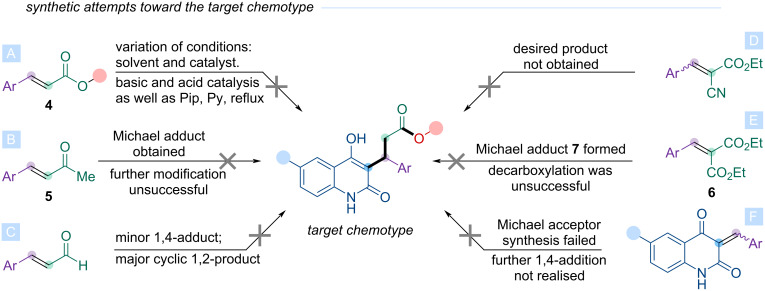
Previous synthetic attempts toward the target chemotype using various approaches.

The 6-halo-4-hydroxyquinolin-2-ones **2a**–**c** were then subjected to Michael addition reactions with various acceptors following the retrosynthetic approaches (Scheme 2), and the results are discussed below (Scheme 4).

No Michael adducts were obtained from reactions of **2a**–**c** with α,β-unsaturated esters, such as (*E*)-3-(3,4-dimethoxyphenyl)acrylic acid esters **3** (see Supporting Information File 1) exemplified by compound **4**. Different solvents and catalysts, both acidic and basic, were screened at room temperature, including refluxing in pyridine with piperidine, but the desired 1,4-addition adduct was not detected (Scheme 4A). Michael adducts formed with α,β-unsaturated methyl ketones (e.g., compound **5**). However, the subsequent haloform reaction failed to produce the target acid, yielding only trace amounts of a complex, difficult-to-separate mixture (Scheme 4B). Also reactions of α,β-unsaturated aldehydes with **2a**–**c** afforded only minor amounts of the 1,4-addition product, while the cyclic 1,2-addition product predominated (Scheme 4C). The reaction of α,β-unsaturated cyanoacrylate with **2c** also failed to yield the desired product (Scheme 4D).

For the reaction of α,β-unsaturated malonate **6** with **2a**–**c**, various bases (sodium methoxide, triethylamine, potassium hydroxide) and solvents (ethanol, DMF, pyridine) were tested, mostly at room temperature, with an additional attempt at refluxing in ethanol/DMF. The desired malonate Michael adduct **7** (isolated and fully characterized, see Supporting Information File 1) was obtained exclusively when potassium hydroxide was used as base in the reaction between **2b** and arylidenemalonate **6** in DMF at room temperature.

The malonate Michael adduct **7** was then subjected to various decarboxylation conditions. The Krapcho protocol (NaCl or NaCN, wet DMSO, 160 °C) failed to yield the target product. Acidic and alkaline hydrolysis followed by decarboxylation also proved unsuccessful (Scheme 4E).

Subsequently, following the second retrosynthetic approach (Scheme 2), compounds **2b** and **2c** were reacted with 3,4-dimethoxybenzaldehyde via Michael 1,4-addition. No condensation products were obtained and the reaction mixtures consisted solely of starting materials. It is likely that under these conditions, the nucleophilicity of 4-hydroxyquinolinones was insufficient to attack the aldehyde carbonyl, preventing product formation (Scheme 4F).

Based on the results obtained, it can be concluded that a sufficiently reactive Michael acceptor is likely necessary to achieve successful 1,4-addition with the relatively weak nucleophile that is 4-hydroxyquinolin-2-one. Among the acceptors tested, arylidene malonates showed the best performance (Scheme 4E). However, the Michael adducts obtained could not be effectively decarboxylated.

To overcome this limitation, we proposed replacing the arylidene malonate with an analogue that allows easier decarboxylation. Meldrum’s acid derivatives, specifically 5-arylidene-2,2-dimethyl-1,3-dioxane-4,6-dione, were considered promising candidates due to the presence of two electron-withdrawing groups and their well-known facile decarboxylation properties. Literature reports indicated that Meldrum’s acid can introduce into molecule fragments containing the CH_2_C(O)R moiety, where R depends on the nucleophile in the reaction mixture [44–45].

Previous studies have reported the synthesis of compounds structurally related to our targets – 4-aryl-4,6-dihydro-2*H*-pyrano[3,2-*c*]quinoline-2,5(3*H*)-diones – via multicomponent reactions catalyzed by various agents (Scheme 1) [37–39]. These one-pot reactions presumably proceed through in situ formation of the Michael acceptor from the condensation of an aromatic aldehyde with Meldrum’s acid, followed by decarboxylation and lactonization to yield the pyranoquinolinedione products (Scheme 1).

We hypothesized that employing an alcohol as solvent could induce nucleophilic attack by the alcohol on the carbonyl group of the Meldrum’s acid fragment, thereby favoring formation of an open-chain ester rather than a lactone upon decarboxylation.

Following this rationale, the reaction of 4-hydroxyquinolin-2-ones **2a**–**c**, 3,4-dimethoxybenzaldehyde, and Meldrum’s acid in boiling methanol in the presence of ʟ-proline yielded the corresponding methyl esters **10a**–**c** in good yields (57–69%). Similarly, using ethanol under the same conditions afforded the ethyl esters **9a**–**c** with moderate yields (46–50%).

**Scheme 5:**
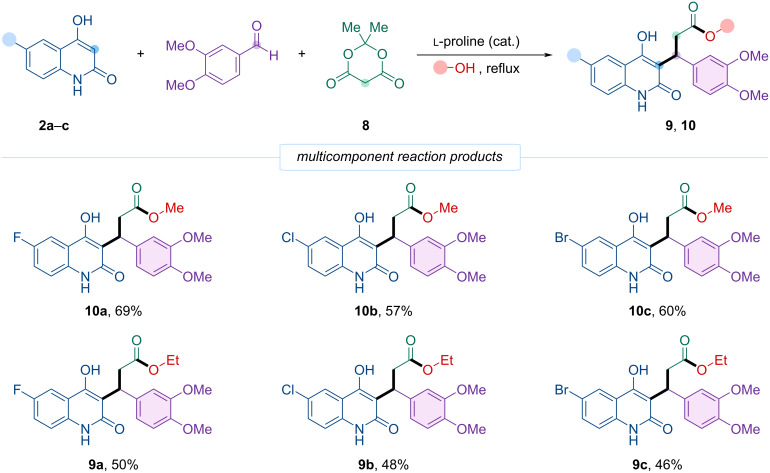
Four-component synthesis of 3-(6-halo-4-hydroxy-2-oxo-1,2-dihydroquinolin-3-yl)-3-(3,4-dimethoxyphenyl)propanoic acid esters **9** and **10**.

Thus, we developed a novel multicomponent organocatalyzed cascade reaction involving 4-hydroxyquinolinones **2a**–**c**, veratraldehyde, and Meldrum’s acid in alcohol (methanol or ethanol) with ʟ-proline catalysis. Importantly, by employing the alcohol simultaneously as both a reactant and the reaction medium and by fine-tuning the reaction conditions, this cascade can be deliberately diverted from the formation of the kinetically favored pyranoquinolinone intermediates toward thermodynamically controlled alcoholysis, ultimately affording open-chain quinolinone esters. As a result, the transformation efficiently produces the corresponding methyl and ethyl esters **10a**–**c** and **9a**–**c** in good yields (Scheme 5).

**Scheme 6:**
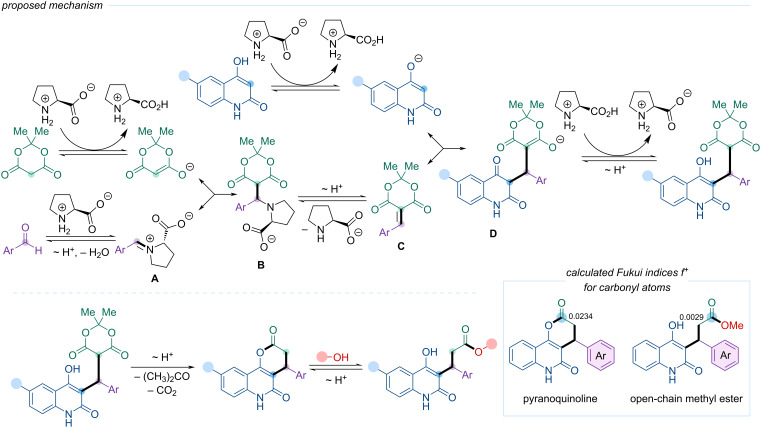
The proposed mechanism of the four-component reaction.

The proposed mechanism of the four-component reaction is illustrated in Scheme 6. ʟ-Proline, via its amino group, nucleophilically attacks the carbonyl of the aromatic aldehyde, forming an iminium zwitterion intermediate **A**. Concurrently, ʟ-proline promotes the generation of the Meldrum's acid anion, which then undergoes Michael addition to iminium zwitterion **A**. The resulting Michael adduct **B** eliminates deprotonated ʟ-proline through proton migration, yielding arylidene Meldrum's acid **C**.

Simultaneously, ʟ-proline facilitates the formation of the 4-hydroxyquinolin-2-one anion, which undergoes Michael addition to arylidene intermediate **C**. The thus formed adduct **D** is protonated by ʟ-proline and undergoes tautomerization.

During the initial experiments to synthesizing methyl and ethyl esters via this four-component reaction, we observed the parallel formation of an undesired cyclic product, pyranoquinolinone. The targeted open-chain esters were mainly obtained by refluxing the corresponding alcohol for several days. Optimal yields of these esters required extended reaction times and elevated temperatures. Shorter reaction durations or lower temperatures (30–50 °C) favored the formation of the cyclic lactone, which predominated under such conditions.

These findings suggest that intramolecular attack by the quinolinone OH group, leading to cyclization and formation of pyranoquinolinone, proceeds faster than nucleophilic attack by the external alcohol. Thus, under mild or brief reaction conditions, the lactone forms predominantly (Scheme 1B). However, the lactone appears capable of slow transesterification in the presence of excess alcohol, converting it into the open-chain ester upon prolonged heating.

The lower stability of the cyclic product, compared to the open-chain ester, is likely due to the electronic effects within pyranoquinoline. As an enol ester conjugated with an amide and an aromatic ring, electron-withdrawing groups decrease electron density on the enol OH, while aliphatic substituents (methyl, ethyl) increase electron density on the alcohol oxygen. This renders the pyranoquinoline carbonyl more electrophilic than that of the acyclic ester, facilitating its alcoholysis at elevated temperature and extended reaction times. Consequently, pyranoquinolines may be regarded as kinetic products, formed rapidly under mild conditions, whereas the open-chain esters represent thermodynamically favored, more stable products formed upon prolonged heating.

To support the hypothesis that the carbonyl carbon atom in pyranoquinoline exhibits higher electrophilicity than in the open-chain ester, quantum chemical calculations were performed to evaluate their electrophilic properties (see Supporting Information File 1, pages S3 and S50–S51). The methyl ester was selected as the model open-chain compound. Calculations revealed that the initial atomic charge on the carbonyl carbon in the lactone was +0.234, whereas in the methyl ester it was slightly lower at +0.225.

Further quantification was performed using a set of quantum chemical descriptors that describe electrophilic properties of an atom: Fukui atomic index, *f*^+^ [46], condensed local electrophilicity index, ω, condensed local softness, *s*^+^, and relative electrophilicity index, *s*^+^/*s^−^* [47] (Table 1). These quantities consistently confirmed the trend: greater electrophilicity is observed for the carbonyl carbon in pyranoquinoline, corroborating the experimental observations.

**Table 1:** Electron density descriptors (Fukui atomic index, *f^+^*; condensed local electrophilicity index, ω; condensed local softness, *s*^+^; relative electrophilicity index *s^+^*/*s^−^*) calculated for the carbonyl carbon atom in pyranoquinoline and in the open-chain ester (see Scheme 6).

	*f*^+^, e^a^	ω, e·eV	*s^+^*, Hartree·e	s^+^/s^−^

pyranoquinoline	0.0234	0.02613	0.0785	3.9874
methyl ester	0.0029	0.00277	0.0098	0.9910

^a^e is the elementary charge unit.

Based on these results, the proposed mechanism of the multicomponent reaction involves initial lactone formation, followed by ring opening (transesterification/alcoholysis) under the influence of alcohol, ultimately yielding the open-chain ester (Scheme 6). This mechanistic rationale explains both the observed selectivity and the intrinsic limitations of the reaction scope imposed by the dual role of the alcohol as nucleophile and solvent.

Interestingly, replacing alcohols with secondary amines (e.g., pyrrolidine) as the reaction medium did not lead to the desired amides. Experimental trials resulted in the formation of complex mixtures (as confirmed by ^1^H NMR), suggesting that the high reactivity of amines interferes with the selectivity of the cascade. This highlights the unique role of the alcohol component as a mild nucleophile and a compatible solvent in our protocol.

To obtain the isopropyl esters of 3-(6-halogen-4-hydroxy-2-oxo-1,2-dihydroquinolin-3-yl)-3-(3,4-dimethoxyphenyl)propanoic acids, a four-component reaction was attempted using isopropanol as the solvent under the established conditions. This resulted in a mixture of the open-chain isopropyl ester and the corresponding cyclic product, with the latter predominating. When a mixture of isopropanol and benzene (1:1.5) was employed as the solvent, no isopropyl ester was formed; pyranoquinoline was the major product. Attempts at equilibrium transesterification using sodium isopropylate in isopropanol also failed to yield the desired product. Similar efforts to synthesize cyclohexyl esters via this approach were unsuccessful.

**Scheme 7:**
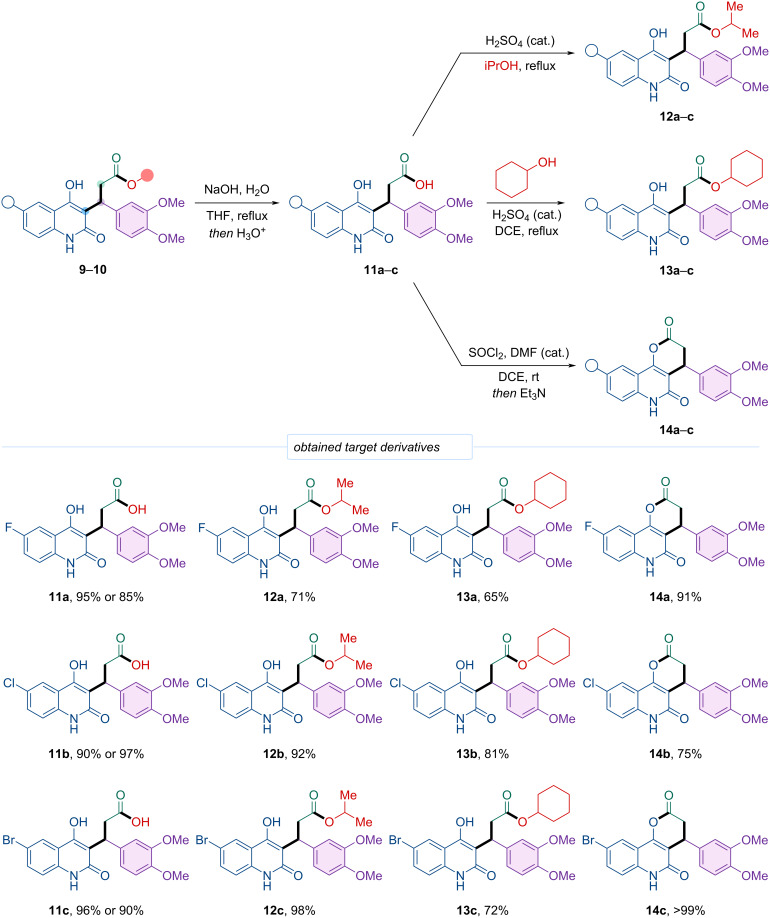
Synthesis of isopropyl (**12a**–**c**) and cyclohexyl (**13а**–**с**) esters of 3-(4-hydroxy-2-oxo-1,2-dihydroquinolin-3-yl)-3-phenylpropanoic acids **11а**–**с** and pyranoquinolines **14a**–**c** and product scope.

Due to the inability to obtain the target isopropyl and cyclohexyl esters directly via the four-component reaction, an alternative synthetic route was employed (Scheme 7). The corresponding acids **11a**–**c**, derived from hydrolysis of methyl esters **10a**–**c** and ethyl esters **9a–c** with 5 M aqueous alkali in refluxing THF, were isolated in high yields (85–97%). Subsequent esterification of acids **11a**–**c** afforded the isopropyl esters **12a**–**c** in 71–98% yields.

Similar attempts were made using cyclohexyl alcohol both as reagent and solvent in Fischer–Speier esterification; however, difficulties in purifying the reaction mixtures due to the large excess of alcohol prevented isolation of the desired products. Employing in situ acyl chloride formation from acids **11a**–**c** in the presence of a slight excess of cyclohexanol led to pyranoquinoline **14** formation instead of the open-chain ester. The same cyclic product **14** was obtained via Fischer–Speier esterification with a small excess of cyclohexanol in refluxing dichloromethane. Moreover, Steglich esterification (using DCC and DMAP in dichloromethane), known for its mild conditions and compatibility with sterically hindered substrates, also produced the cyclic pyranoquinoline **14** rather than the expected ester. During preparative chromatography on silica gel with a methanol-containing eluent, isolated pyranoquinoline **14** underwent alcoholysis, converting it into the corresponding methyl ester **10** at the column outlet. These observations further support the mechanistic considerations of the four-component reaction discussed earlier.

Optimized conditions for synthesizing the target cyclohexyl esters **13a**–**c** involved treatment of acids **11a**–**c** with a refluxing mixture of cyclohexanol and 1,2-dichloroethane in the presence of concentrated sulfuric acid. Using a substantial excess of cyclohexanol diluted with 1,2-dichloroethane allowed efficient reaction progress and facile isolation of pure products, with yields ranging from 65 to 81%.

Pyranoquinolines **14a**–**c** can be prepared from acids **11a**–**c** via Fischer–Speier esterification, Steglich esterification, or intramolecular acylation through in situ formation of acyl chlorides. The cyclic products were also obtained via the four-component reaction using non-nucleophilic acetonitrile as solvent. However, this method requires subsequent purification by preparative chromatography, and, as noted, pyranoquinolines **14a**–**c** readily undergo alcoholysis on silica columns with methanol-containing eluents.

Among these methods, in situ acyl chloride formation using thionyl chloride in the presence of DMF in 1,2-dichloroethane is advantageous due to low cost, straightforward reaction handling, high yields, and product purity. After a short reaction time, triethylamine was added, affording pyranoquinolines **14a**–**c** in excellent yields of 75–100%.

While the initial stages of the cascade resemble previously reported multicomponent protocols leading to pyranoquinolinone scaffolds, our study demonstrates that fine-tuning the reaction conditions allows deliberate diversion toward open-chain ester derivatives. These 3-(4-hydroxyquinolin-3-yl)propanoate esters were previously inaccessible, as earlier protocols consistently afforded cyclic pyranoquinolinones.

Pyranoquinolinones are the kinetic products, whereas the open-chain esters represent the thermodynamically favored species formed via controlled alcoholysis under prolonged reflux. This insight resolves a long-standing selectivity issue and transforms a known cascade into a synthetically divergent, operationally simple one-pot process. The selective access to methyl and ethyl esters is synthetically valuable, serving as versatile intermediates for subsequent conversion into isopropyl and cyclohexyl esters.

### Cytotoxicity and antibacterial activity

Selected synthesized compounds were evaluated for their antibacterial activity and cytotoxicity. The compounds exhibited poor water solubility but were readily soluble in DMSO. Experimental details are provided in Supporting Information File 1, pages S3 and S52–S53.

Cytotoxicity of compounds **9–14** was assessed in vitro using an MTT assay [48] on a panel of human cell lines, including non-cancerous HEK293T and VA13, as well as cancerous MCF7 and A549 cells (see Supporting Information File 1, page S52). Most compounds showed no significant cytotoxicity at concentrations up to 100 μmol/L. However, compounds **10a**, **10b**, and **10c** demonstrated considerable toxicity toward both the rapidly proliferating HEK293T and the slowly proliferating VA13 cell lines. This cytotoxicity pattern contrasts with that of the well-known topoisomerase II inhibitor doxorubicin (see Supporting Information File 1, page S52), whose toxicity correlates with the cell proliferation rate. Compounds **13b** and **13c** exhibited non-specific toxicity across all tested cell lines.

**Figure 3:**
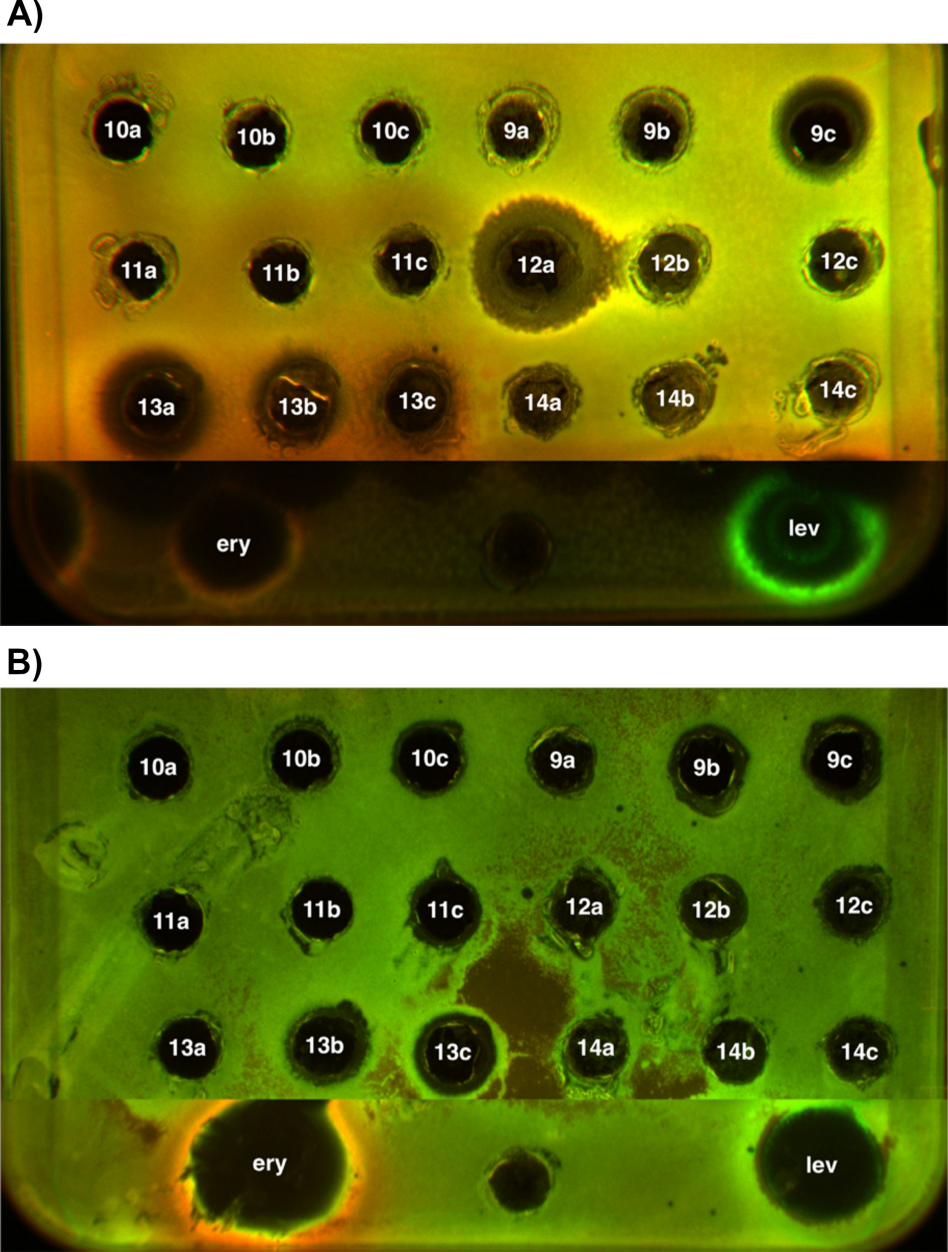
In vitro antibacterial activity studies. А) In vitro antibacterial activity using the *E. coli* ΔtolC strain with modified reflux system; B) in vitro antibacterial activity using the *E. coli* lptD mut strain with modified cell membrane; ery – erythromycin, lev – levofloxacin.

Antibacterial activities of compounds **9**–**14** were evaluated in vivo using the diffusion-in-agar method [49] against *Escherichia coli* ΔtolC and *E. coli* lptD mutant strains. None of the compounds exhibited activity against the lptD mutant. Compound **12a** showed the broadest growth inhibition zone, with compounds **9c** and **13a** also demonstrating significant effects against the ΔtolC strain, which is more susceptible due to its compromised efflux system. The minimum inhibitory concentrations (MICs) of compounds **13a**, **9c**, and **12a** against the *E. coli* lptD mutant strain were 400 μmol/L (Figure 3 and Supporting Information File 1, Table S1).

Importantly, compounds **12a**, **9c**, and **13a** inhibited the growth of the *E. coli* ΔtolC strain while exhibiting minimal cytotoxicity toward human cell lines, even at concentrations up to 100 μmol/L. Their cytotoxicity profile differs markedly from that of known topoisomerase II inhibitors.

Although the synthesized compounds did not demonstrate pronounced antibacterial activity, their low toxicity toward human cells and distinctive molecular scaffold render them promising candidates for further development as biologically active agents.

## Conclusion

In conclusion, we have developed a novel organocatalyzed four-component cascade reaction that provides the first direct and atom-economical access to structurally elusive 3-(4-hydroxy-2-oxo-1,2-dihydroquinolin-3-yl)-3-phenylpropanoate esters. These open-chain esters represent a scarcely explored motif within the quinolinone family, which are typically inaccessible through conventional Michael addition strategies that favor pyranoquinoline formation.

The synthetic approach employs readily available 6-halo-4-hydroxyquinolin-2(1*H*)-ones, aromatic aldehydes, Meldrum’s acid, and alcohols under mild ʟ-proline-catalyzed conditions, affording a range of esters in good to excellent yields. Mechanistic studies suggest a cascade sequence involving pyranoquinoline intermediates followed by thermodynamically driven alcoholysis in the presence of excess alcohol solvent at elevated temperatures.

Thus, although the initial bond-forming events share similarities with previously reported multicomponent cascades, the present study establishes a fundamentally different synthetic outcome, enabling access to open-chain quinolinone esters that were previously inaccessible within this reaction framework.

The utility of the method was further demonstrated through optimized one-pot syntheses and efficient hydrolysis and esterification protocols. This concise and sustainable strategy offers a valuable platform for accessing medicinally relevant quinolinone scaffolds and holds promise for the development of bioactive compounds featuring this privileged core.

Although significant antibacterial activity was not observed in the tested biological models, the findings provide a solid foundation for further structure–activity relationship (SAR) studies aimed at identifying key structural features responsible for the predicted biological effects. Importantly, the absence of cytotoxicity confirms the pharmacological potential of the 4-hydroxyquinolin-2(1*H*)-one scaffold as a versatile template for future targeted modifications to develop active derivatives.

## Supporting Information

File 1 Experimental procedures and analytical characterization of all synthesized compounds.

## Data Availability

All data that supports the findings of this study is available in the published article and/or the supporting information of this article.
