# Supplementary material for: A mild and atom-efficient four-component cascade strategy for the construction of biologically relevant 4-hydroxyquinolin-2(1H)-one derivatives
Source: Beilstein J Org Chem. 2026 Feb 9;22:244–56. doi: 10.3762/bjoc.22.18 (PMC12908358; doi:10.3762/bjoc.22.18)
Supplement: File 1 — Experimental procedures and analytical characterization of all synthesized compounds. [file Beilstein_J_Org_Chem-22-244-s001.pdf]

BEILSTEIN JOURNAL OF ORGANIC CHEMISTRY

Supporting Information

for

A mild and atom-efficient four-component cascade strategy for the construction of biologically relevant 4-hydroxyquinolin-2(1*H*)-one derivatives

Dmitrii A. Grishin, Kseniia I. Sharkovskaia, Ilya G. Kolmakov, Daria A. Ipatova, Rostislav A. Petrov, Nikolai D. Dagaev, Dmitry A. Skvortsov, Maria G. Khrenova, Valeriy V. Andreychev, Sergei A. Evteev, Yan A. Ivanenkov, Roman L. Antipin, Olga A. Dontsova and Elena K. Beloglazkina

Beilstein J. Org. Chem. **2026,** *22,* 244–256. doi:10.3762/bjoc.22.18

Experimental procedures and analytical characterization of all synthesized compounds

License and Terms: This is a supporting information file under the terms of the Creative Commons Attribution License (https://creativecommons.org/licenses/by/4.0). Please note that the reuse, redistribution and reproduction in particular requires that the author(s) and source are credited and that individual graphics may be subject to special legal provisions.
The license is subject to the *Beilstein Journal of Organic Chemistry* terms and conditions: (https://www.beilstein-journals.org/bjoc/terms)

Table of contents

General information S2

Chemistry S2

Computational chemistry S3

Biology S3

Synthesis of compounds S4

Synthesis of *N*^{1},*N*^{3}-diphenylmalonamides S4

Synthesis of 4-hydroxyquinolin-2(1*H*)-ones S6

Synthesis of (*E*)-3-(3,4-dimethoxyphenyl)acrylic acid (**3**) S9

Synthesis of methyl (*E*)-3-(3,4-dimethoxyphenyl)acrylate (**4**) S9

Synthesis of (*E*)-4-(3,4-dimethoxyphenyl)but-3-en-2-one (**5**) S10

Synthesis of diethyl 2-(3,4-dimethoxybenzylidene)malonate (**6**) S11

Synthesis of diethyl 2-((6-chloro-4-hydroxy-2-oxo-1,2-dihydroquinolin-3-yl)(3,4-dimethoxyphenyl)methyl)malonate (**7**) S11

Synthesis of 2,2-dimethyl-1,3-dioxane-4,6-dione (Meldrum's acid, **8**) S12

Synthesis of target ethyl esters S13

Synthesis of target methyl esters S16

Synthesis of target acids S19

Synthesis of target isopropyl esters S23

Synthesis of target cyclohexyl esters S26

Synthesis of target pyranoquinolines S29

NMR spectra of synthesized novel compounds S32

Equilibrium geometry configurations of considered molecules S50

In vitro antibacterial activity studies S52

Minimum inhibitory concentration evaluation S52

References S54

General information

Chemistry

Control over the course of reactions and purity of chromatographic separation of substances was carried out by thin-layer chromatography (TLC) on Merck TLC Silica gel 60 F254 plates. MN Kieselgel 60 0.04–0.063 mm/230–400 mesh ASTM silica gel was used for preparative chromatography. UV lamp (254, 365 nm), potassium permanganate solution, ninhydrin were used to show the stains.

^{1}H, ^{13}C, ^{19}F NMR spectra were recorded on a Bruker Avance-400 instrument with operating frequencies of 400, 100, and 376 MHz. $CDCl_3$, DMSO-d_6 were used as solvents. Chemical shifts are given in parts per million (ppm) on the δ scale and are calibrated based on the residual peak of the deuterated solvent. The coupling constants *J* are given in hertz (Hz). The following abbreviations were used for the allocation of signal multiplicities: bs – broad singlet, s – singlet, d – doublet, dd – doublet of doublets, ddd – doublet of doublets of doublets, t – triplet, dt – doublet of triplets, td – triplet of doublets, tt – triplet of triplets, q – quartet, dq – doublet of quartets, qd – quartet of doublets, sept – septet, m – multiplet.

LCMS: HPLC–MS analysis of samples was performed using LC-20 Promlinence liquid chromatograph (Shimadzu, Japan) and LCMS-2020 quadrupole chromatomass spectrometer (Shimadzu, Japan) equipped with a DUIS + ESI electrospray ionization source. Registration was performed on a Jupiter C4 column (phenomenex, USA), 150 × 4.6 mm, 5 μm particle size. Registration was performed in gradient mode, deionized water (with addition of 0.1% formic acid) and acetonitrile were used as mobile phase. The mobile phase feed rate was 1.0 mL/min, and the column temperature was 40 °C. Ionization of analytes was carried out under electrospray conditions (DUIS+ESI), source parameters: desolvation line heating temperature 250 °C, sample ionization unit heating temperature 400 °C, spray gas – 1.5 L/min, drying gas – 15 L/min, capillary voltage 4500 V (in negative mode −4500 V). Mass spectra were recorded in the range of *m/z* 170–2000, scanning speed 1875 μs.

HRMS: the study was performed using a system consisting of a LC-30 Nexera liquid chromatograph (Shimadzu, Japan) equipped with a DGU-20A degasser, two LC-30AD chromatographic pumps, a CTO-20A column thermostat and an Orbitrap QExactive Plus high-resolution mass spectrometer (Thermo Scientific, USA) with a mass analyzer based on an orbital ion trap. Methanol was used as the sample solvent and mobile phase. We injected 0.2 μL of the sample into a 0.2 mL/min stream of methanol without chromatographic separation directly into the ion source. The analysis time is 2 minutes. The high-resolution mass spectrometer was operated in the mode of recording positively and negatively charged ions under electrospray ionization (ESI) conditions. FullMS scanning mode. Ion source parameters: pressure of drying gas – 12, atomizing gas and curtain gas fluxes – 4 and 1

conventional units, desolvation line temperature – 320 °C, voltage on the needle – 3.8 kV (ESI+) and 3.2 kV (ESI−), RF voltage on the S-lens – 55 uC. Mass spectra were recorded in the range of *m/z* 100–2500 with a spectral resolution value equal to 70000. Mass spectrometer control, data acquisition and primary data processing were performed using Xcalibur software (Thermo Scientific, USA).

IR spectra were recorded on a Thermo Scientific Nicolet iS5 FT-IR spectrometer. The spectra were recorded in KBr pellets at a resolution of 4 cm^{-1}, the number of scans was 20.

Commercially available reagents were used without further purification. Solvents were purified and absolutized according to standard techniques.

Computational chemistry

Geometry optimizations were initially performed using the composite B97-3c method with the def2-TZVPP basis set using ORCA software. Atomic charges were calculated using the Hirshfeld scheme based on the B3LYP density functional with the def2-TZVPP basis set.

Subsequently, quantum chemical calculations of the equilibrium geometry configuration of selected molecules were performed at the ωB97x-D3/def2-SVP [1,2] Kohn-Sham DFT level using ORCA software [3]. Electron density analysis was performed using Multiwfn program package [4]. The following descriptors were calculated to estimate electrophilic properties of the carbonyl carbon atom : Fukui atomic indices, f^{+} [5], condensed local electrophilicity index, ω, condensed local softness, s^{+}, and relative electrophilicity index s^{+}/s^{-} [6].

Biology

MTT assay: The MTT cytotoxicity assay was conducted following the procedure outlined in [7] using the Janus automatic station (PerkinElmer). A cell suspension (2500 cells for HEK293T and A549; 5000 cells for VA13 and 6000 for MCF7) in 140 μL of Dulbecco's modified Eagle medium (DMEM)/F12 was added to 96-well plates. The cells were incubated at 37 °C in a CO_2 incubator for 24 hours. Stock solutions of drugs in DMSO (20 mM) were prepared and diluted with a complete medium just before the experiment. The maximum compound concentration used was 100 μmol/L, and the DMSO concentration did not exceed 0.5% by volume. All experiments were performed in triplicate. After 72 hours of incubation with tested substances, MTT reagent (3-(4,5-dimethylthiazol-2-yl)-2,5-diphenyltetrazolium bromide) (Paneco) was added to the cells to a final concentration of 0.5 g/L. The cells were incubated with the MTT reagent for 2–4 hours at 37 °C in a CO_2 incubator. Afterward, the medium containing the MTT reagent was removed, and 120 μL of DMSO was added. The cells were incubated with DMSO for at least 10 minutes at room temperature on an orbital shaker to dissolve the formazan formed during MTT reduction. The optical absorption of formazan was measured at 565 nm using a VICTOR X5 Plate Reader. A four-parameter nonlinear regression model was applied to calculate the IC_{50abs}.

Synthesis of compounds

Synthesis of N^1,N^3-diphenylmalonamides

Synthesis of N^1,N^3-bis(4-fluorophenyl)malonamide (1a)

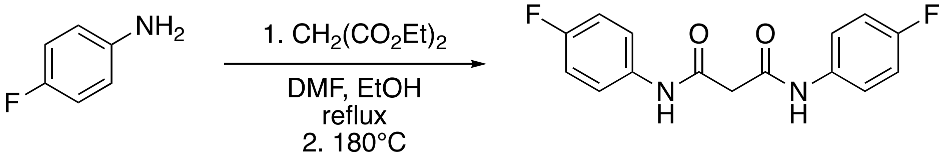

4 mL of 4-fluoroaniline (42 mmol, 2.2 equiv), 15 mL EtOH, 2.9 mL (19 mmol, 1 equiv) diethyl malonate and 100 μL DMF were placed in a round-bottomed 100 mL flask equipped with a magnetic stirrer. The reaction mixture was intensively mixed, gradually increasing the temperature in the oil bath to 180 °C. First, the reaction mixture was heated with a Liebig condenser installed, into which cold water was supplied. After that, the heating of the reaction mixture continued, turning off the water supply to the Liebig condenser, thereby converting it into an air condenser. After a few hours, with a decrease in temperature, the solidification of the reaction mass and the formation of the product in the form of gray crystals were observed. Cold EtOH was added to the cooled crushed reaction mass, and precipitation of a gray product was observed. The flask was placed in the freezer for a while to complete the precipitation of the product, which was subsequently filtered, washed with a small amount of cold EtOH and dried in a drying cabinet at 100 °C. As a result, a pure product was obtained in the form of a powder weighing 3.59 g with a yield of 65%.

^{1}H NMR spectrum (400 MHz, DMSO-d_6) δ 10.23 (s, 2H, NH), 7.66 – 7.56 (m, 4H, H_{Ar}), 7.15 (t, J = 8.9 Hz, 4H, H_{Ar}), 3.44 (s, 2H, CH_2).

The spectroscopic data correspond to those given earlier in the literature [8].

Synthesis of N^1,N^{3-}bis(4-chlorophenyl)malonamide (1b)

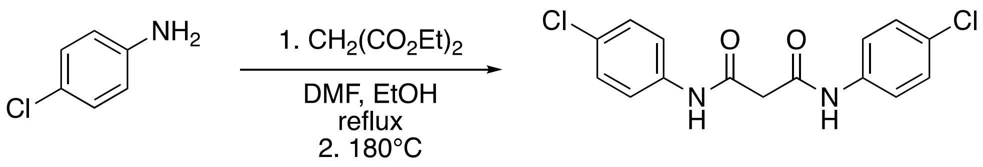

5.12 g (40 mmol, 2.2 equiv) of 4-chloroaniline, 15 mL of EtOH, 2.8 mL (18 mmol, 1 equiv) of diethyl malonate and 100 μL of DMF were placed in a 100 mL round-bottomed flask equipped with a magnetic stirrer. The reaction mixture was intensively mixed, gradually increasing the temperature in the oil bath to 180 °C. First, the reaction mixture was heated with a Liebig condenser installed, into which cold water was supplied. After that, the heating of the reaction mixture continued, turning off the water supply to the Liebig condenser, thereby converting it into an air condenser. After several hours, at lowering temperature, solidification of the reaction mass and formation of the product in the

form of yellowish crystals were observed. Cold EtOH was added to the cooled crushed reaction mass and a light-yellow precipitate of the product was observed. The flask was placed in the freezer for a while to complete the precipitation of the product, which was subsequently filtered, washed with a small amount of cold EtOH and dried in a drying cabinet at 100 °C. As a result, a pure product was obtained in the form of a powder weighing 4.31 g with a yield of 72%.

^{1}H NMR spectrum (400 MHz, DMSO-d_6) δ 10.32 (s, 2H, NH), 7.69 – 7.54 (m, 4H, H_{Ar}), 7.44 – 7.28 (m, 4H, H_{Ar}), 3.46 (s, 2H, CH_2).

The spectroscopic data correspond to those given earlier in the literature [8].

Synthesis of *N*^{1},*N*^{3}-bis(4-bromophenyl)malonamide (1c)

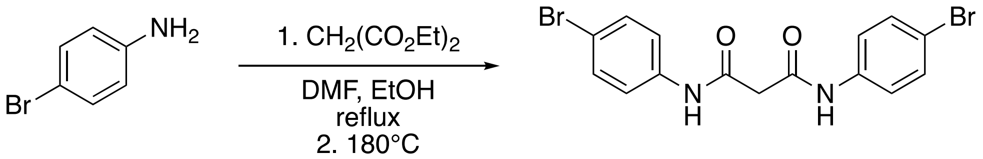

5.3 g (31 mmol, 2.2 equiv) of 4-bromaniline, 15 mL of EtOH, 2.14 mL (14 mmol, 1 equiv) of diethyl malonate and 100 μL of DMF were placed in a 100 mL round-bottomed flask equipped with a magnetic stirrer. The reaction mixture was intensively mixed, gradually increasing the temperature in the oil bath to 180 °C. First, the reaction mixture was heated with a Liebig condenser installed, into which cold water was supplied. After that, the heating of the reaction mixture continued, turning off the water supply to the Liebig condenser, thereby converting it into an air condenser. After a few hours, when the temperature was lowered, solidification of the reaction mass and formation of the product in the form of light gray crystals were observed. Cold EtOH was added to the cooled crushed reaction mass, and a light gray precipitate of the product was observed. The flask was placed in the freezer for a while to complete the precipitation of the product, which was subsequently filtered, washed with a small amount of cold EtOH and dried in a drying cabinet at 100 °C. As a result, a pure product was obtained in the form of a powder weighing 2.98 g with a yield of 52%.

^{1}H NMR spectrum (400 MHz, DMSO-d_6) δ 10.31 (s, 2H, NH), 7.57 (d, J = 9.0 Hz, 4H, H_{Ar}), 7.48 (d, J = 9.0 Hz, 4H, H_{Ar}), 3.47 (s, 2H, CH_2).

The spectroscopic data are consistent with those reported earlier in the literature [9].

Synthesis of 4-hydroxyquinolin-2(1*H*)-ones

Synthesis of 6-fluoro-4-hydroxyquinolin-2(1*H*)-one (2a)

Method 1:

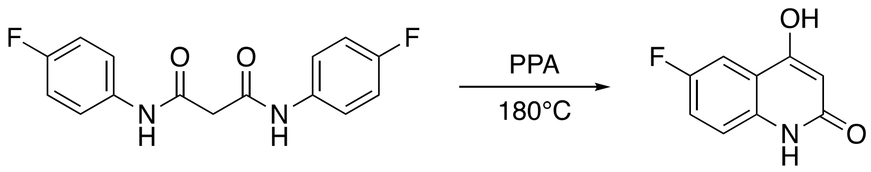

3.57 g (12 mmol) of dry powder N^1,N^3-bis(4-fluorophenyl)malonamide (**1a**) was added to a 100 mL round-bottomed flask equipped with a magnetic stirrer. PPA was added in an amount sufficient to completely absorb the entire volume of malonamide in the flask. The resulting thick mass was left to stir vigorously under heating (oil bath temperature ≈180 °C) with a chlorocalcium tube. After some time, ice water was added to the thick dark colored reaction mass. The precipitated dark green precipitate was filtered, washed with water until the filtrate was neutral. The wet residue on the filter was dried in a drying cabinet at 100 °C. As a result, the pure product was obtained as a powder weighing 2.09 g in quantitative yield.

Method 2:

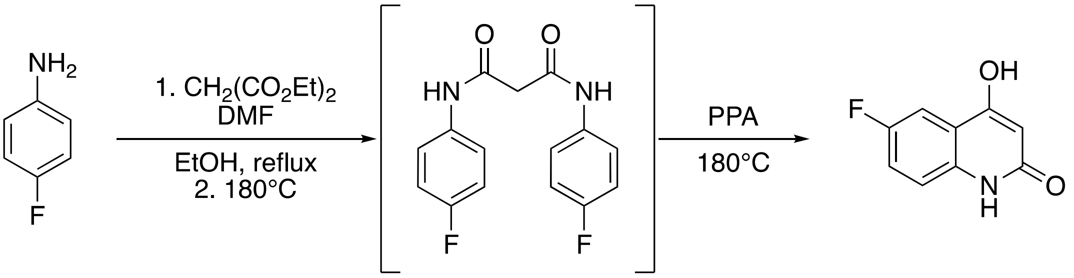

In a 100 mL round-bottomed flask equipped with a magnetic stirrer and a reflux condenser, 3.42 mL of 4-fluoroaniline (36 mmol, 2.2 equiv), 15 mL of EtOH, 2.48 mL of diethyl malonate (16.4 mmol, 1 equiv) and 100 μL of DMF were placed. The resulting mixture was boiled for one hour. An air condenser was installed and the temperature of the oil bath was raised to 180 °C. The synthesis was then carried out under these conditions for several hours. After a few hours a dry solid product was formed. PPA was added to the reaction mixture in an amount that allowed to completely dissolve the contents of the flask. The reaction mixture was stirred intensively at 180 °C for several hours. After the reaction mixture had cooled down, it was poured into a beaker with ice. The formation of a grey precipitate was observed. Cold water was added and the mixture was stirred at room temperature for 30 minutes. The precipitate formed was filtered off and washed to neutral on a Schott filter. The wet residue was dried in a drying cabinet at 115 °C. As a result, the pure product was obtained as a powder weighing 2.44 g in 83% yield.

^{1}**H NMR spectrum** (400 MHz, DMSO-d_6) δ 11.48 (bs, 1H, OH), 11.30 (s, 1H, NH), 7.45 (dd, *J* = 9.3, 2.9 Hz, 1H, H_{Ar}), 7.38 (td, *J* = 8.7, 3.0 Hz, 1H, H_{Ar}), 7.27 (dd, *J* = 9.0, 4.8 Hz, 1H, H_{Ar}), 5.77 (s, 1H, C(OH)=C**H**).

The spectroscopic data are consistent with those reported earlier in the literature [8].

Synthesis of 6-chloro-4-hydroxyquinolin-2(1*H*)-one (2b)

Method 1:

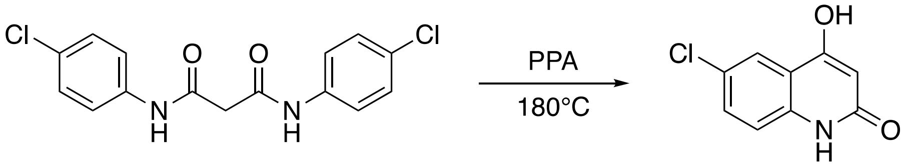

4.31 g (13 mmol) of dry powder N^1,N^3-bis(4-chlorophenyl)malonamide (**1b**) was added to a 100 mL round-bottomed flask equipped with a magnetic stirrer. PPA was added in an amount sufficient to completely absorb the entire volume of malonamide in the flask. The resulting thick mass was left to stir vigorously under heating (oil bath temperature ~180 °C) with a chlorocalcium tube. After some time, ice water was added to the thick reaction mass of yellow-green color. The precipitated light green precipitate was filtered, washed with water until the filtrate was neutral. The wet residue on the filter was dried in a drying cabinet at 100 °C. As a result, the pure product was obtained as a powder weighing 2.53 g in quantitative yield.

Method 2:

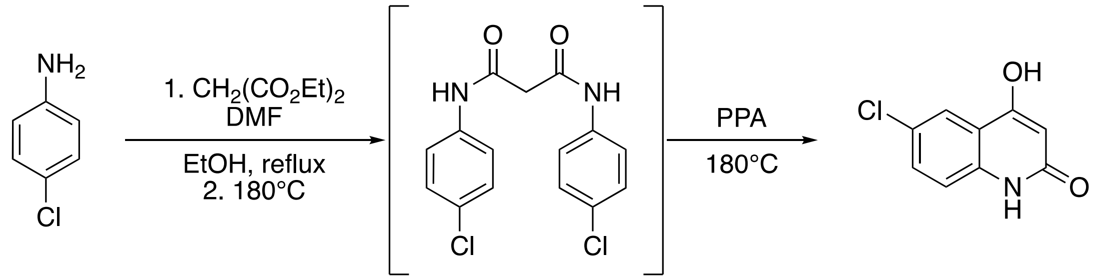

In a 100 mL round-bottomed flask equipped with a magnetic stirrer and a reflux condenser, 11.7 g (91.77 mmol, 2 equiv) of 4-chloroaniline, 15 mL of EtOH, 7 mL (46.10 mmol, 1 equiv) of diethyl malonate and 1 mL of DMF were placed. The resulting mixture was boiled for one hour. Having installed an air condenser, the temperature of the oil bath was raised to 180 °C. The synthesis was then carried out under these conditions for several hours. After a few hours a dry solid product was formed. PPA was added to the reaction mixture in an amount that allowed to completely dissolve the contents of the flask. The reaction mixture was stirred intensively at 180 °C for several hours. After the reaction mixture had cooled down, it was poured into a beaker with ice. The formation of a greenish-yellow precipitate was observed. Cold water was added and the mixture was stirred at room

temperature for 30 minutes. The resulting precipitate was filtered off and washed to neutral on a Schott filter. The wet residue was dried in a drying cabinet at 115 °C. As a result, the pure product was obtained as a powder weighing 6.9 g in 77% yield.

^{1}H NMR spectrum (400 MHz, DMSO-*d*6) δ 11.57 (bs, 1H, OH), 11.34 (s, 1H, NH), 7.70 (d, *J* = 2.4 Hz, 1H, H_{Ar}), 7.52 (dd, *J* = 8.8, 2.4 Hz, 1H, H_{Ar}), 7.26 (d, *J* = 8.8 Hz, 1H, H_{Ar}), 5.76 (s, 1H, C**H**=C(OH)).

The spectroscopic data are consistent with those reported earlier in the literature [8].

Synthesis of 6-bromo-4-hydroxyquinolin-2(1*H*)-one (2c)

Method 1:

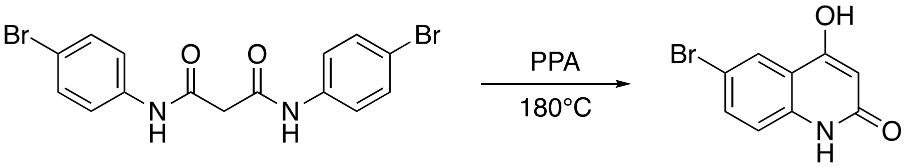

2.4 g (6 mmol) of dry powder *N*^{1},*N*^{3}-bis(4-bromophenyl)malonamide (**1c**) was added to a 100 mL round-bottomed flask equipped with a magnetic stirrer. PPA was added in an amount sufficient to completely absorb the entire volume of malonamide in the flask. The resulting thick mass was left to stir vigorously under heating (oil bath temperature ≈180 °C) with a chlorocalcium tube. After some time, ice water was added to the thick reaction mass of dark green color. The precipitated light green precipitate was filtered, washed with water until the filtrate was neutral. The wet residue on the filter was dried in a drying cabinet at 100 °C. As a result, the pure product was obtained as a powder weighing 1.4 g in quantitative yield.

Method 2:

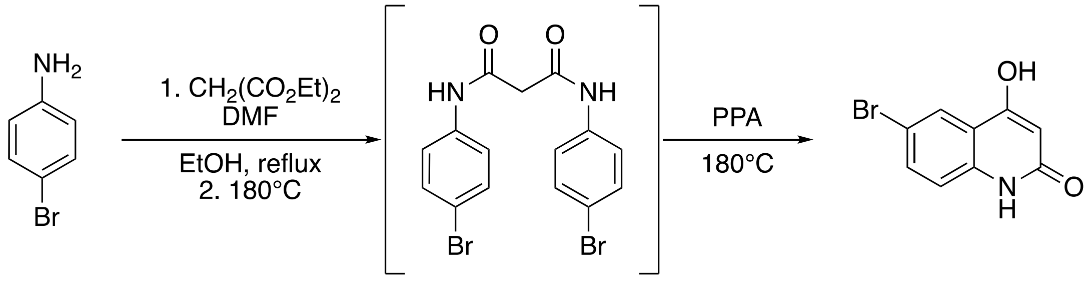

4.0 g (23.25 mmol, 2.2 equiv) of 4-bromaniline, 15 mL of EtOH, 1.6 mL (10.57 mmol, 1 equiv) of diethyl malonate and 100 μL of DMF were placed in a 100 mL round-bottomed flask equipped with a magnetic stirrer and a reflux condenser. The resulting mixture was boiled for one hour. An air condenser was set up and the temperature of the oil bath was raised to 180 °C. The synthesis was then carried out under these conditions for several hours. After a few hours a dry solid product was formed. PPA was added to the reaction mixture in an amount that allowed to completely dissolve the contents

of the flask. The reaction mixture was stirred intensively at 180 °C for several hours. After the reaction mixture cooled down, it was poured into a beaker with ice. The formation of a greenish colored precipitate was observed. Cold water was added and the mixture was stirred at room temperature for 30 minutes. The precipitate formed was filtered off and washed to neutral on a Schott filter. The wet residue was dried in a drying cabinet at 115 °C. As a result, the pure product was obtained as a powder weighing 2.1 g in 82% yield.

^{1}H NMR spectrum (400 MHz, DMSO-d_6) δ 11.56 (bs, 1H, OH), 11.35 (s, 1H, NH), 7.84 (d, J = 2.3 Hz, 1H, H_{Ar}), 7.63 (dd, J = 8.7, 2.3 Hz, 1H, H_{Ar}), 7.20 (d, J = 8.8 Hz, 1H, H_{Ar}), 5.74 (s, 1H, C**H**=C(OH)).

The spectroscopic data are consistent with those reported earlier in the literature [10].

Synthesis of (*E*)-3-(3,4-dimethoxyphenyl)acrylic acid (3)

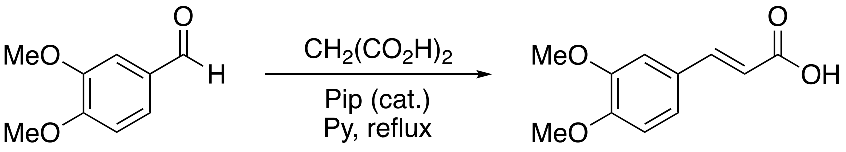

In a 100 mL round-bottomed flask equipped with a magnetic stirrer and a reflux condenser, 1.00 g (6 mmol, 1 equiv) of 3,4-dimethoxybenzaldehyde, 1.25 g (12 mmol, 2 equiv) of malonic acid, 50 mL of pyridine and 320 μL of piperidine were added. The reaction mixture was stirred at boiling (oil bath temperature 120 °C). The formation of the reaction product was monitored by TLC. The solvent was removed from the reaction mixture under reduced pressure. Then ≈50 mL of 1 M HCl solution was added. The formed white precipitate was filtered off, washed with water and then dried in the drying cabinet. As a result, the pure product was obtained as a white powder weighing 1.19 g in 95% yield.

^{1}H NMR spectrum (400 MHz, $CDCl_3$) δ 7.74 (d, J = 15.8 Hz, 1H, C_{Ar}HC=), 7.14 (dd, J = 8.3, 2.0 Hz, 1H, H_{Ar}), 7.08 (d, J = 2.0 Hz, 1H, H_{Ar}), 6.88 (d, J = 8.3 Hz, 1H, H_{Ar}), 6.33 (d, J = 15.9 Hz, 1H, =C**H**CO_2H), 3.93 (s, 6H, 2×OMe)

The spectroscopic data are consistent with those reported earlier in the literature [11]

Synthesis of methyl (*E*)-3-(3,4-dimethoxyphenyl)acrylate (4)

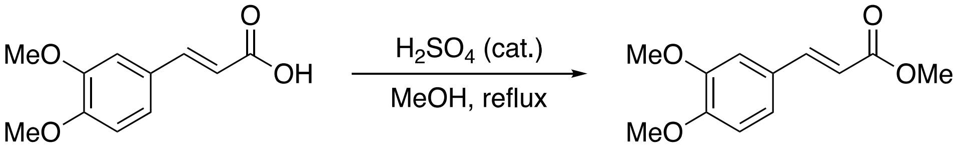

In a 100 mL round-bottomed flask equipped with a magnetic stirrer and a reflux condenser, 0.45 g (2.2 mmol) of (*E*)-3-(3,4-dimethoxyphenyl)acrylic acid (**3**) and 20 mL of MeOH were placed.

The resulting mixture was heated (to an oil bath temperature of 60 °C) while stirring, and 250 μL of concentrated H_2SO_4 was added after complete dissolution of the substrate. The reaction mixture was heated to boiling (oil bath temperature ≈120 °C) and further synthesis was carried out under these conditions. The formation of the reaction product was monitored by TLC (PE/EtOAc (1:1)). The resulting reaction mixture was concentrated under reduced pressure. A saturated $NaHCO_3$ solution was added. The reaction mixture was extracted with EtOAc. The obtained organic phase was washed with saturated NaCl solution, dried over anhydrous Na_2SO_4, concentrated under reduced pressure. The residue obtained was an oil, gradually crystallizing, dried under reduced pressure. The product was then separated individually by column chromatography (SiO_2, eluent PE/EtOAc (2:1)). As a result, the pure product was obtained as a white crystalline mass weighing 0.47 g in 97% yield.

^{1}**H NMR spectrum** (400 MHz, $CDCl_3$) δ 7.61 (d, *J* = 15.9 Hz, 1H, C_{Ar}HC=), 7.08 (dd, *J* = 8.3, 1.9 Hz, 1H, H_{Ar}), 7.02 (d, *J* = 1.9 Hz, 1H, H_{Ar}), 6.84 (d, *J* = 8.3 Hz, 1H, H_{Ar}), 6.29 (d, *J* = 15.9 Hz, 1H, =C**H**CO_2Me), 3.89 (s, 6H, 2×OMe), 3.77 (s, 3H, CO_2Me).

The spectroscopic data are consistent with those reported earlier in the literature [12].

Synthesis of (*E*)-4-(3,4-dimethoxyphenyl)but-3-en-2-one (5)

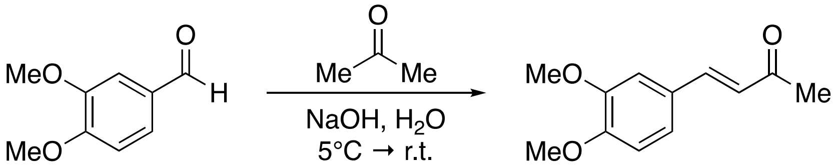

1.5 g (9.07 mmol) of veratraldehyde and 10 mL of acetone were added to a 250 mL three-necked flask placed in an ice bath and equipped with a magnetic stirrer, a thermometer, a dropping funnel and an air condenser. From the dropping funnel, a solution of 0.5 g (12.5 mmol) NaOH in 10 mL water was slowly added to the reaction mixture while stirring. The temperature of the mixture was maintained at ≈5 °C and the synthesis was carried out under these conditions. The formation of the reaction product was monitored by TLC. Then, 1 M HCl solution was added to the reaction mixture, acetone was removed under reduced pressure and the mixture was extracted with EtOAc. The organic phase was washed with water and saturated NaCl solution. The resulting organic layer was dried over anhydrous Na_2SO_4, concentrated and dried under reduced pressure. The product was isolated individually by preparative chromatography. The resulting product was obtained with a mass of 1.55 g in 83% yield.

^{1}**H NMR spectrum** (400 MHz, $CDCl_3$) δ 7.44 (d, *J* = 16.1 Hz, 1H, C**H**=CHC(O)Me), 7.10 (dd, *J* = 8.3, 2.0 Hz, 1H, H_{Ar}), 7.05 (d, *J* = 2.0 Hz, 1H, H_{Ar}), 6.86 (d, *J* = 8.3 Hz, 1H, H_{Ar}), 6.58 (d, *J* = 16.2 Hz, 1H, CH=C**H**C(O)Me), 3.89 (s, 6H, 2×OMe), 2.34 (s, 3H, C(O)Me).

The spectroscopic data are consistent with those reported earlier in the literature [13]

Synthesis of diethyl 2-(3,4-dimethoxybenzylidene)malonate (6)

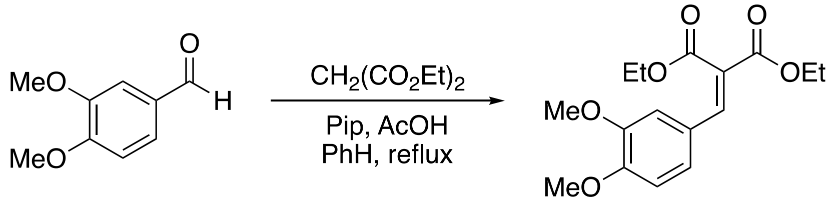

3.00 g (18 mmol, 1 equiv) of veratraldehyde, 3.55 mL (23 mmol, 1.3 equiv) of diethyl malonate, 250 μL of piperidine, 90 μL of concentrated acetic acid and 40 mL of benzene were placed in a 100 mL round-bottomed flask equipped with a magnetic stirrer, a Dean–Stark apparatus and a reflux condenser. The reaction mixture was stirred at boiling. The extent of reaction product formation was monitored by TLC. The mixture was concentrated and dried under reduced pressure. The product was isolated individually by preparative chromatography (SiO_2, eluent PE/EtOAc (10:1) → (5:1) → (2:1) → (1:1)). As a result, the pure product was obtained as a light yellow colored oil weighing 1.91 g in 34% yield.

^{1}H NMR spectrum (400 MHz, $CDCl_3$) δ 7.63 (s, 1H, C_{Ar}CH=), 7.06 (dd, *J* = 8.4, 2.1 Hz, 1H, H_{Ar}), 7.00 (d, *J* = 2.1 Hz, 1H, H_{Ar}), 6.83 (d, *J* = 8.4 Hz, 1H, H_{Ar}), 4.32 (q, *J* = 7.1 Hz, 2H, C**H**$_2$CH$_3$), 4.26 (q, *J* = 7.1 Hz, 2H, C**H**$_2$CH$_3$), 3.88 (s, 3H, OMe), 3.83 (s, 3H, OMe), 1.29 (t, *J* = 7.1 Hz, 6H, 2×CH$_2$C**H**$_3$).

The spectroscopic data are consistent with those reported earlier in the literature [14]

Synthesis of diethyl 2-((6-chloro-4-hydroxy-2-oxo-1,2-dihydroquinolin-3-yl)(3,4-dimethoxyphenyl)methyl)malonate (7)

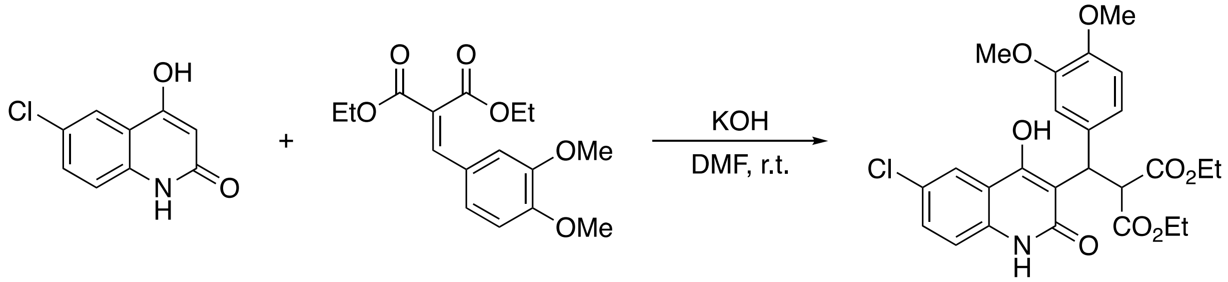

0.30 g (1.54 mmol, 1.2 equiv) of 6-chloro-4-hydroxyquinoline-2(1*H*)-one (**2b**), 0.11 g (2.04 mmol, 1.6 equiv) of potassium hydroxide and 10 mL of DMF were placed in a 100 mL round-bottomed flask equipped with a magnetic stirrer. The resulting mixture was stirred for about half an hour under argon atmosphere. A solution of 0.39 g (1.27 mmol, 1 equiv) of malonate **6** in 5 mL of DMF was added to the reaction mixture. The reaction mixture was further stirred at room temperature. The extent of product formation was monitored by TLC (DCM/MeOH (20:1)). DMF was distilled off from the reaction mixture under reduced pressure. 1 M HCl solution was added and precipitation was

observed. Column chromatography (SiO_2, eluent DCM/MeOH (75:1) → (50:1) → (20:1), dry application) was carried out. As a result, the product was obtained with a mass of 0.22 g in 34% yield.

^{1}H NMR spectrum (400 MHz, DMSO-d_6) δ 11.49 (s, 1H, NH), 10.81 (s, 1H, OH), 7.98 (d, J = 2.3 Hz, 1H, H_{Ar}), 7.49 (dd, J = 8.8, 2.3 Hz, 1H, H_{Ar}), 7.22 (d, J = 8.8 Hz, 1H, H_{Ar}), 7.15 (s, 1H, H_{Ar}), 7.00 (d, J = 8.3 Hz, 1H, H_{Ar}), 6.80 (d, J = 8.4 Hz, 1H, H_{Ar}), 5.23 (br.s, 1H, C**H**($CO_2Et)_2$), 5.08 (d, J = 11.9 Hz, 1H, C**H**CH($CO_2Et)_2$), 3.95 (qd, J = 7.1, 2.7 Hz, 4H, 2×C**H**$_2$$CH_3$), 3.68 (s, 3H, OMe), 3.67 (s, 3H, OMe), 0.98 (t, J = 7.1 Hz, 3H, CH_2C**H**$_3$), 0.94 (t, J = 7.1 Hz, 3H, CH_2C**H**$_3$).

ESI-HRMS (*m/z*): calc. for $[C_{25}H_{27}ClNO_8]^+$ 504.1420, found 504.1421 $[M+H]^+$.

Synthesis of 2,2-dimethyl-1,3-dioxane-4,6-dione (Meldrum's acid) (8)

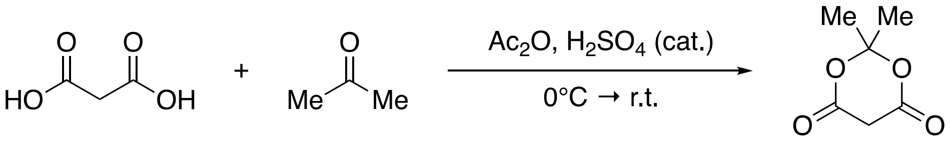

10.4 g (0.1 mol, 1 equiv) of malonic acid and 12 mL (0.127 mol, 1.3 equiv) of acetic anhydride were added to a chemical beaker fitted with a magnetic stirrer, placed in an ice bath with sodium chloride. The resulting mixture was cooled under vigorous stirring and 0.3 mL (5.6 mmol, 0.04 equiv) of concentrated H_2SO_4 was added slowly at ≈0 °C. Next, 8 mL (0.1 mol, 1 equiv) of acetone was gradually added to the reaction mixture from a dropping funnel, taking care that the temperature did not exceed 15 °C. After addition of acetone was completed, the ice bath was removed and the reaction mixture was stirred until it reached room temperature. After that it was stirred for another 10 minutes at room temperature. The obtained mixture in a beaker was placed in the freezer overnight. The resulting crystalline mass was crushed, transferred to a Schott filter, washed with 5 ml of cold 5% H_2SO_4 solution and ice water. The resulting white mass was dried on a Schott filter at reduced pressure for several hours. As a result, a white colored powder of 8.7 g was obtained in 60% yield.

^{1}H NMR spectrum (400 MHz, $CDCl_3$) δ 3.63 (s, 2H, CH_2), 1.79 (s, 6H, CH_3).

The spectroscopic data are consistent with those reported earlier in the literature [15].

Synthesis of target ethyl esters

Synthesis of ethyl 3-(3,4-dimethoxyphenyl)-3-(6-fluoro-4-hydroxy-2-oxo-1,2-dihydroquinolin-3-yl)propanoate (9a)

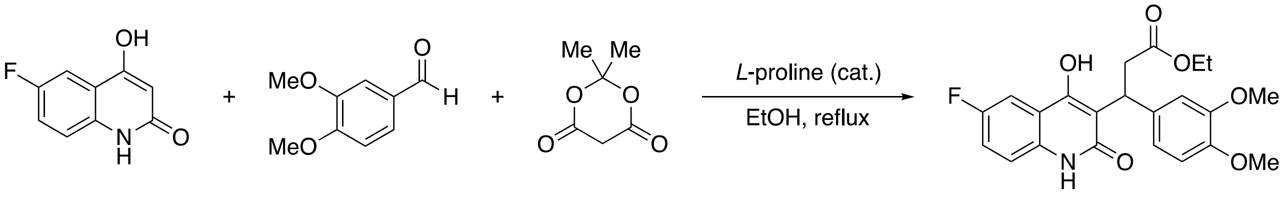

0.54 g (3 mmol, 1 equiv) of 6-fluoro-4-hydroxyquinoline-2(1*H*)-one (**2a**) and 50 mL of ethanol were placed in a 100 mL round-bottomed flask equipped with a magnetic stirrer and a reflux condenser, and the resulting suspension was stirred vigorously and gradually heated. Then 0.5 g (3 mmol, 1 equiv) of 3,4-dimethoxybenzaldehyde, 0.44 g (3 mmol, 1 equiv) of Meldrum's acid **8** and 0.033 g (0.3 mmol, 0.1 equiv) of L-proline were added. The reaction mixture was stirred vigorously at boiling (oil bath temperature ≈100 °C) for a day. The extent of reaction product formation was monitored by TLC (eluent CH_2Cl_2/MeOH (20:1)). The reaction mixture was applied to silica gel for subsequent dry application to the column. The individual substance was isolated by column chromatography (SiO_2, eluent CH_2Cl_2/MeOH (30:1), dry application). As a result, the pure product was obtained as a yellow colored powder weighing 0.62 g in 50% yield.

^{1}H NMR spectrum (400 MHz, DMSO-d_6) δ 11.37 (s, 1H, NH), 10.50 (s, 1H, OH), 7.72 (dd, *J* = 10.2, 2.8 Hz, 1H, H_{Ar}), 7.35 (td, *J* = 8.7, 2.8 Hz, 1H, H_{Ar}), 7.26 (dd, *J* = 9.0, 4.9 Hz, 1H, H_{Ar}), 7.06 (d, *J* = 2.1 Hz, 1H, H_{Ar}), 6.90 (dd, *J* = 8.5, 2.0 Hz, 1H, H_{Ar}), 6.80 (d, *J* = 8.4 Hz, 1H, H_{Ar}), 4.93 (t, *J* = 7.9 Hz, 1H, CH), 3.95 (qd, *J* = 7.1, 1.0 Hz, 2H, C**H**$_2$CH$_3$), 3.69 (s, 3H, OMe), 3.68 (s, 3H, OMe), 3.36 (dd, *J* = 15.6, 8.4 Hz, 1H, CHC**H**$_2$), 3.24 (dd, *J* = 15.6, 7.5 Hz, 1H, CHC**H**$_2$), 1.04 (t, *J* = 7.1 Hz, 3H, CH$_2$C**H**$_3$).

^{13}C NMR spectrum (100 MHz, DMSO-d_6) δ 172.10 (**C**O$_2$Et), 162.81 (C(O)NH), 156.98 (d, *J* = 2.6 Hz, **C**(OH)=C), 156.7 (d, *J* = 236.7 Hz, C_{Ar}F), 148.2 (**C$_{Ar}$**OCH$_3$), 147.2 (**C$_{Ar}$**OCH$_3$), 135.6 (C_{Ar}), 134.4 (C_{Ar}), 119.7 (C_{Ar}), 118.13 (d, *J* = 24.7 Hz, C_{Ar}), 116.78 (d, *J* = 8.5 Hz, C_{Ar}), 116.02 (d, *J* = 8.1 Hz, C_{Ar}), 114.9 (C(OH)=**C**), 111.9 (C_{Ar}), 111.5 (C_{Ar}), 107.93 (d, *J* = 24.4 Hz, C_{Ar}), 59.55 (**C**H$_2$CH$_3$), 55.5 (OCH$_3$), 55.4 (OCH$_3$), 36.6 (CH**C**H$_2$), 36.0 (**C**HCH$_2$), 14.0 (CH$_2$**C**H$_3$).

^{19}F NMR spectrum (376 MHz, DMSO-d_6) δ −121.30 (td, *J* = 9.7, 9.3, 5.1 Hz).

ESI-HRMS (*m/z*): calc. for $[C_{22}H_{21}FNO_6]^-$ 414.1353, found 414.1363 $[M-H]^-$.

ESI-LCMS (*m/z*): calc. for $[C_{22}H_{21}FNO_6]^-$ 414.1, found 414.2 $[M-H]^-$ (99.423%).

Synthesis of ethyl 3-(6-chloro-4-hydroxy-2-oxo-1,2-dihydroquinolin-3-yl)-3-(3,4-dimethoxyphenyl)propanoate (9b)

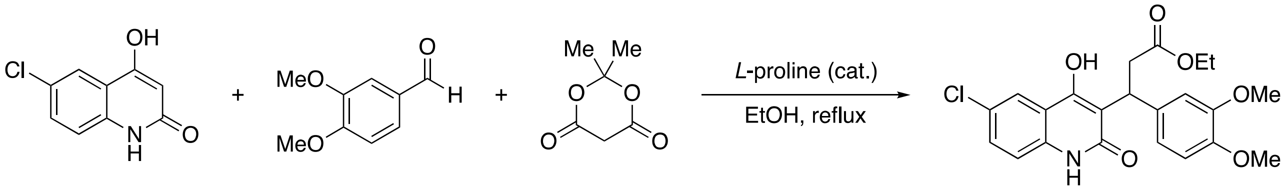

0.405 g (2 mmol, 1 equiv) of 6-chloro-4-hydroxyquinoline-2(1*H*)-one (**2b**) and ≈15 mL of ethanol were placed in a 100 mL round-bottomed flask equipped with a magnetic stirrer and a reflux condenser, and the resulting suspension was stirred vigorously and gradually heated. Then 0.337 g (2 mmol, 1 equiv) of 3,4-dimethoxybenzaldehyde, 0.315 g (2 mmol, 1 equiv) of Meldrum's acid **8** and 0.024 g (0.2 mmol, 0.1 equiv) of L-proline were added. The reaction mixture was stirred vigorously at boiling (oil bath temperature ≈100 °C) for a day. The extent of reaction product formation was monitored by TLC. The reaction mixture was spread on silica gel for subsequent dry column application. The individual substance was isolated by column chromatography (SiO_2, eluent CH_2Cl_2/MeOH (30:1), dry application). As a result, the pure product was obtained as a yellow colored powder weighing 0.431 g in 48% yield.

^{1}H NMR spectrum (400 MHz, DMSO-d_6) δ 11.42 (s, 1H, NH), 10.55 (s, 1H, OH), 7.96 (d, J = 2.3 Hz, 1H, H_{Ar}), 7.48 (dd, J = 8.7, 2.3 Hz, 1H, H_{Ar}), 7.24 (d, J = 8.8 Hz, 1H, H_{Ar}), 7.03 (d, J = 2.1 Hz, 1H, H_{Ar}), 6.88 (dd, J = 8.4, 2.1 Hz, 1H, H_{Ar}), 6.79 (d, J = 8.4 Hz, 1H, H_{Ar}), 4.91 (t, J = 7.9 Hz, 1H, CH), 4.00 – 3.88 (d, 2H, C**H**$_2$CH$_3$), 3.69 (s, 3H, OMe), 3.68 (c, 3H, OMe), 3.31 (d, J = 8.3 Hz, 1H, CHC**H**$_2$), 3.22 (dd, J = 15.6, 7.5 Hz, 1H, CHC**H**$_2$), 1.03 (t, J = 7.1 Hz, 3H, CH$_2$C**H**$_3$).

^{13}C NMR spectrum (100 MHz, DMSO-d_6) δ 172.1 (**C**O$_2$Et), 162.9 (C(O)NH), 156.7 (**C**(OH)=C), 148.2 (**C$_{Ar}$**OCH$_3$), 147.2 (**C$_{Ar}$**OCH$_3$), 136.4 (C_{Ar}), 135.5 (C_{Ar}), 130.0 (C_{Ar}), 125.1 (C_{Ar}), 122.0 (C_{Ar}), 119.7 (C_{Ar}), 116.8 (C_{Ar}), 116.5 (C_{Ar}), 115.1 (C(OH)=**C**), 111.9 (C_{Ar}), 111.5 (C_{Ar}), 59.6 (**C**H$_2$CH$_3$), 55.5 (OCH$_3$), 55.4 (OCH$_3$), 36.6 (CH**C**H$_2$), 36.0 (CH), 14.0 (CH$_2$**C**H$_3$).

ESI-HRMS (*m/z*): calc. for $[C_{22}H_{21}ClNO_6]^-$ 430.1057, found 430.1066 $[M-H]^-$.

ESI-LCMS (*m/z*): calc. for $[C_{22}H_{23}ClNO_6]^+$ 432.1, found 432.2 $[M+H]^+$ (98.321%).

Synthesis of ethyl 3-(6-bromo-4-hydroxy-2-oxo-1,2-dihydroquinolin-3-yl)-3-(3,4-dimethoxyphenyl)propanoate (9c)

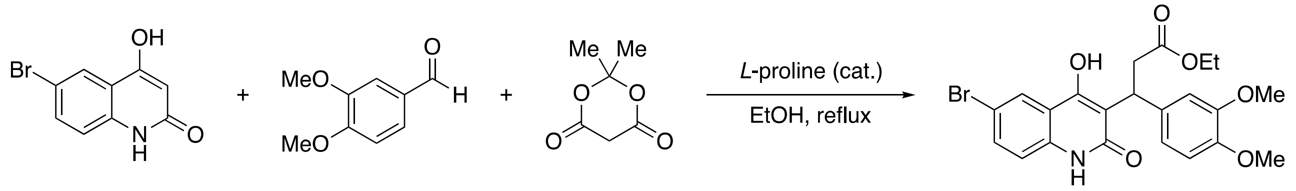

0.483 g (2 mmol, 1 equiv) of 6-bromo-4-hydroxyquinoline-2(1*H*)-one (**2c**) and ≈15 mL of ethanol were placed in a 100 mL round-bottomed flask equipped with a magnetic stirrer and a reflux condenser, and the resulting suspension was stirred vigorously and gradually heated. Then 0.333 g (2 mmol, 1 equiv) of 3,4-dimethoxybenzaldehyde, 0.288 g (2 mmol, 1 equiv) of Meldrum's acid **8** and 0.042 g (0.36 mmol, 0.2 equiv) of L-proline were added. The reaction mixture was stirred vigorously at boiling (oil bath temperature ≈100 °C) for a day. The extent of reaction product formation was monitored by TLC. The reaction mixture was spread on silica gel for subsequent dry column application. The individual substance was isolated by column chromatography (SiO_2, eluent CH_2Cl_2/MeOH (30:1), dry application). As a result, the pure product was obtained as a light yellow powder weighing 0.436 g in 46% yield.

^{1}H NMR spectrum (400 MHz, DMSO-*d*6) δ 11.42 (s, 1H, NH), 10.55 (s, 1H, OH), 8.09 (d, *J* = 2.2 Hz, 1H, H_{Ar}), 7.59 (dd, *J* = 8.7, 2.2 Hz, 1H, H_{Ar}), 7.17 (d, *J* = 8.8 Hz, 1H, H_{Ar}), 7.03 (d, *J* = 2.0 Hz, 1H, H_{Ar}), 6.87 (dd, *J* = 8.4, 2.0 Hz, 1H, H_{Ar}), 6.79 (d, *J* = 8.3 Hz, 1H, H_{Ar}), 4.90 (t, *J* = 7.9 Hz, 1H, CH), 3.94 (q, *J* = 7.0 Hz, 2H, C**H**$_2$CH$_3$), 3.68 (s, 3H, OMe), 3.68 (s, 3H, OMe)., 3.38 – 3.28 (m, 1H, CHC**H**$_2$), 3.22 (dd, *J* = 15.6, 7.5 Hz, 1H, CHC**H**$_2$), 1.03 (t, *J* = 7.1 Hz, 3H, CH$_2$C**H**$_3$).

^{13}C NMR spectrum (100 MHz, DMSO-*d*6) δ 172.1 (**C**O$_2$Et), 162.9 (C(O)NH), 156.6 (**C**(OH)=C), 148.2 (**C**$_{Ar}$OCH$_3$), 147.2 (**C**$_{Ar}$OCH$_3$), 136.7 (C_{Ar}), 135.5 (C_{Ar}), 132.7 (C_{Ar}), 125.0 (C_{Ar}), 119.7 (C_{Ar}), 117.1 (C_{Ar}), 117.0 (C_{Ar}), 115.0 (C_{Ar}), 112.8 (C(OH)=**C**), 111.8 (C_{Ar}), 111.5 (C_{Ar}), 59.6 (**C**H$_2$CH$_3$), 55.5 (OCH$_3$), 55.4 (OCH$_3$), 36.6 (CH**C**H$_2$), 36.0 (CH), 14.0 (CH$_2$**C**H$_3$).

ESI-HRMS (*m/z*): calc. for $[C_{22}H_{21}{}^{81}BrNO_6]^-$ 476.0532, found 476.0540 $[M{-}H]^-$.

ESI-LCMS (*m/z*): calc. for $[C_{22}H_{23}{}^{81}BrNO_6]^+$ 478.1, found 478.1 $[M{+}H]^+$ (99.059%).

Synthesis of target methyl esters

Synthesis of methyl 3-(3,4-dimethoxyphenyl)-3-(6-fluoro-4-hydroxy-2-oxo-1,2-dihydroquinolin-3-yl)propanoate (10a)

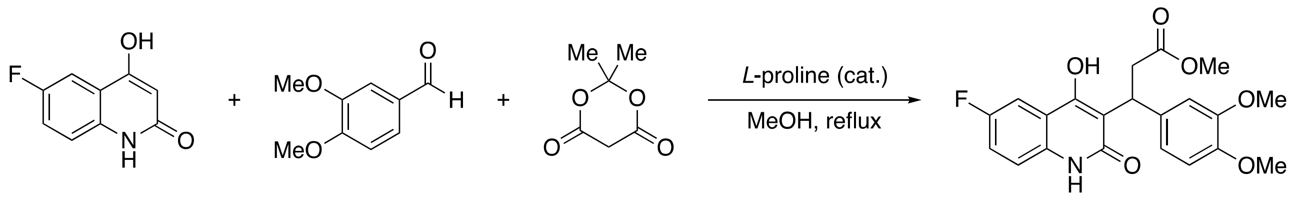

0.537 g (3 mmol, 1 equiv) of 6-fluoro-4-hydroxyquinoline-2(1*H*)-one (**2a**) and 50 mL of methanol were placed in a 100 mL round-bottomed flask equipped with a magnetic stirrer and a reflux condenser, and the resulting suspension was stirred vigorously and gradually heated. Then 0.499 g (3 mmol, 1 equiv) of 3,4-dimethoxybenzaldehyde, 0.432 g (3 mmol, 1 equiv) of Meldrum's acid **8** and 0.0345 g (0.3 mmol, 0.1 equiv) of L-proline were added. The reaction mixture was stirred vigorously at boiling (oil bath temperature ≈95 °C) for a day. The extent of reaction product formation was monitored by TLC. The reaction mixture was spread on silica gel for subsequent dry column application. The individual substance was isolated by column chromatography (SiO_2, eluent CH_2Cl_2/MeOH (30:1), dry application). As a result, the pure product was obtained as a yellow colored powder weighing 0.829 g in 69% yield.

^{1}H NMR spectrum (400 MHz, DMSO-d_6) δ 11.36 (s, 1H, NH), 10.49 (s, 1H, OH), 7.70 (dd, J = 10.2, 2.8 Hz, 1H, H_{Ar}), 7.35 (td, J = 8.7, 2.8 Hz, 1H, H_{Ar}), 7.25 (dd, J = 9.0, 4.9 Hz, 1H, H_{Ar}), 7.04 (d, J = 2.0 Hz, 1H, H_{Ar}), 6.89 (dd, J = 8.4, 2.0 Hz, 1H, H_{Ar}), 6.79 (d, J = 8.3 Hz, 1H, H_{Ar}), 4.92 (t, J = 7.8 Hz, 1H, CH), 3.68 (s, 3H, $ArOCH_3$), 3.68 (s, 3H, $ArOCH_3$), 3.49 (s, 3H, CO_2CH_3), 3.36 (dd, J = 16.0, 8.1 Hz, 1H, CH_2), 3.27 (dd, J = 16.0, 7.6 Hz, 1H, CH_2).

^{13}C NMR spectrum (100 MHz, DMSO-d_6) δ 172.7 (**C**O_2CH_3), 162.8 (C(O)NH), 157.0 (d, J = 2.6 Hz, **C**(OH)=C), 156.8 (d, J = 236.8 Hz, $C_{Ar}F$), 148.2 (**C_{Ar}**OCH_3), 147.2 (**C_{Ar}**OCH_3), 135.6 (C_{Ar}), 134.4 (C_{Ar}), 119.7 (C_{Ar}), 118.2 (d, J = 24.8 Hz, C_{Ar}), 116.8 (d, J = 6.8 Hz, C_{Ar}), 116.0 (d, J = 8.3 Hz, C_{Ar}), 115.0 (C(OH)=**C**), 111.8 (d, J = 3.1 Hz, C_{Ar}), 111.5 (C_{Ar}), 108.0 (d, J = 24.6 Hz, C_{Ar}), 55.4 ($C_{Ar}O$**C**H_3, $C_{Ar}O$**C**H_3), 51.2 (CO_2**C**H_3), 36.4 (CH_2), 36.0 (CH).

^{19}F NMR spectrum (376 MHz, DMSO-d_6) δ −121.24 (ddd, J = 10.0, 8.2, 5.0 Hz).

ESI-HRMS (*m/z*): calc. for $[C_{21}H_{19}FNO_6]^-$ 400.1202, found 400.1203 $[M-H]^-$.

ESI-LCMS (*m/z*): calc. for $[C_{21}H_{19}FNO_6]^-$ 400.12, found 400.25 $[M-H]^-$ (99.217%).

Synthesis of methyl 3-(6-chloro-4-hydroxy-2-oxo-1,2-dihydroquinolin-3-yl)-3-(3,4-dimethoxyphenyl)propanoate (10b)

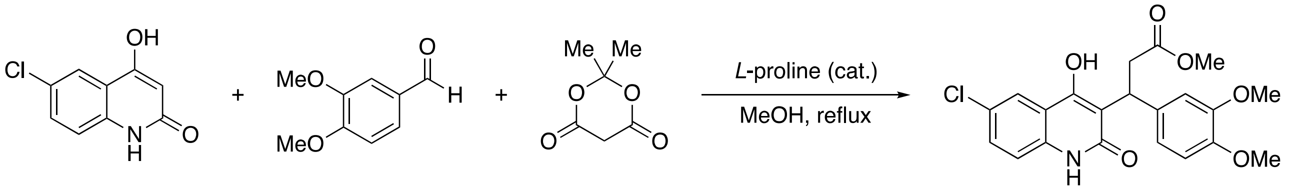

0.782 g (4 mmol, 1 equiv) of 6-chloro-4-hydroxyquinoline-2(1*H*)-one (**2b**) and 40 mL of methanol were placed in a 100 mL round-bottomed flask equipped with a magnetic stirrer and a reflux condenser, and the resulting suspension was stirred vigorously and gradually heated. Then 0.997 g (6 mmol, 1.5 equiv) of 3,4-dimethoxybenzaldehyde, 0.576 g (4 mmol, 1 equiv) of Meldrum's acid **8** and 0.046 g (0.4 mmol, 0.1 equiv) of L-proline were added. The reaction mixture was stirred vigorously at boiling (oil bath temperature ≈95 °C) for a day. The extent of reaction product formation was monitored by TLC. The reaction mixture was spread on silica gel for subsequent dry column application. The individual substance was isolated by column chromatography (SiO_2, eluent CH_2Cl_2/MeOH (30:1), dry application). As a result, the pure product was obtained as a yellow colored powder weighing 0.946 g in 57% yield.

^{1}H NMR spectrum (400 MHz, DMSO-*d*$_6$) δ 11.42 (s, 1H, NH), 10.56 (s, 1H, OH), 7.96 (d, *J* = 2.3 Hz, 1H, H_{Ar}), 7.48 (dd, *J* = 8.9, 2.3 Hz, 1H, H_{Ar}), 7.23 (d, *J* = 8.8 Hz, 1H, H_{Ar}), 7.03 (d, *J* = 2.0 Hz, 1H, H_{Ar}), 6.88 (dd, *J* = 8.3, 1.9 Hz, 1H, H_{Ar}), 6.79 (d, *J* = 8.4 Hz, 1H, H_{Ar}), 4.91 (t, *J* = 7.9 Hz, 1H, CH), 3.69 (s, 3H, $ArOCH_3$), 3.68 (s, 3H, $ArOCH_3$), 3.49 (s, 3H, CO_2CH_3), 3.38 – 3.22 (m, 2H, CH_2).

^{13}C NMR spectrum (100 MHz, DMSO-*d*$_6$) δ 172.6 (**C**O_2CH_3), 162.9 (C(O)NH), 156.7 (**C**(OH)=C), 148.2 (**C$_{Ar}$**OCH_3), 147.2 (**C$_{Ar}$**OCH_3), 136.4 (C_{Ar}), 135.5 (C_{Ar}), 130.0 (C_{Ar}), 125.2 (C_{Ar}), 122.1 (C_{Ar}), 119.6 (C_{Ar}), 116.9 (C_{Ar}), 116.5 (C_{Ar}), 115.1 (C(OH)=**C**), 111.8 (C_{Ar}), 111.5 (C_{Ar}), 55.5 (C_{Ar}O**CH$_3$**), 55.4 (C_{Ar}O**CH$_3$**), 51.2 (CO_2**CH$_3$**), 36.4 (CH_2), 36.0 (CH).

ESI-HRMS (*m/z*): calc. for $[C_{21}H_{19}ClNO_6]^-$ 416.0901, found 416.0913 $[M-H]^-$.

ESI-LCMS (*m/z*): calc. for $[C_{21}H_{21}ClNO_6]^+$ 418.11, found 418.15 $[M+H]^+$ (100%).

Synthesis of methyl 3-(6-bromo-4-hydroxy-2-oxo-1,2-dihydroquinolin-3-yl)-3-(3,4-dimethoxyphenyl)propanoate (10c)

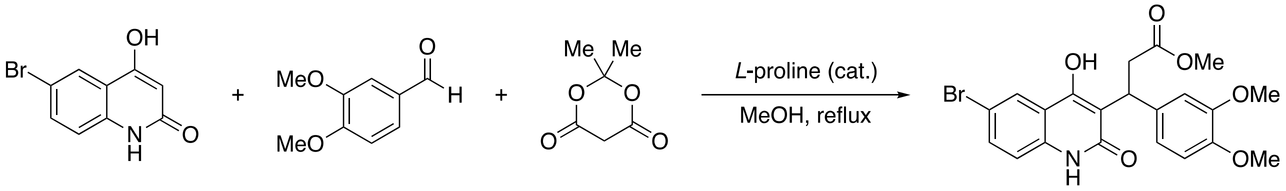

0.720 g (3 mmol, 1 equiv) of 6-bromo-4-hydroxyquinoline-2(1*H*)-one (**2c**) and 50 mL of methanol were placed in a 100 mL round-bottomed flask equipped with a magnetic stirrer and a reflux condenser, and the resulting suspension was stirred vigorously and gradually heated. Then 0.499 g (3 mmol, 1 equiv) of 3,4-dimethoxybenzaldehyde, 0.432 g (3 mmol, 1 equiv) of Meldrum's acid **8** and 0.035 g (0.3 mmol, 0.1 equiv) of L-proline were added. The reaction mixture was stirred vigorously at boiling (oil bath temperature ≈95 °C) for a day. The extent of reaction product formation was monitored by TLC. The reaction mixture was spread on silica gel for subsequent dry column application. The individual substance was isolated by column chromatography (SiO_2, eluent CH_2Cl_2/MeOH (30:1), dry application). As a result, the pure product was obtained as a yellow colored powder weighing 0.832 g in 60% yield.

^{1}H NMR spectrum (400 MHz, DMSO-*d*$_6$) δ 11.42 (s, 1H, NH), 10.56 (s, 1H, OH), 8.09 (d, *J* = 2.2 Hz, 1H, H$_{Ar}$), 7.59 (dd, *J* = 8.7, 2.2 Hz, 1H, H$_{Ar}$), 7.18 (d, *J* = 8.7 Hz, 1H, H$_{Ar}$), 7.03 (d, *J* = 2.0 Hz, 1H, H$_{Ar}$), 6.87 (dd, *J* = 8.4, 2.0 Hz, 1H, H$_{Ar}$), 6.79 (d, *J* = 8.4 Hz, 1H, H$_{Ar}$), 4.90 (t, *J* = 7.8 Hz, 1H, CH), 3.68 (s, 3H, ArOCH$_3$), 3.68 (s, 3H, ArOCH$_3$), 3.49 (s, 3H, CO$_2$CH$_3$), 3.38 – 3.30 (m, 1H, CH$_2$), 3.26 (dd, *J* = 15.9, 7.7 Hz, 1H, CH$_2$).

^{13}C NMR spectrum (100 MHz, DMSO-*d*$_6$) δ 172.6 (**C**O$_2$CH$_3$), 162.8 (C(O)NH), 156.6 (**C**(OH)=C), 148.2 (**C$_{Ar}$**OCH$_3$), 147.2 (**C$_{Ar}$**OCH$_3$), 136.7 (C$_{Ar}$), 135.5 (C$_{Ar}$), 132.7 (C$_{Ar}$), 125.0 (C$_{Ar}$), 119.7 (C$_{Ar}$), 117.1 (C$_{Ar}$), 117.0 (C$_{Ar}$), 115.1 (C$_{Ar}$), 112.8 (C(OH)=**C**), 111.9 (C$_{Ar}$), 111.5 (C$_{Ar}$), 55.5 (C$_{Ar}$O**C**H$_3$), 55,4 (C$_{Ar}$O**C**H$_3$), 51.2 (CO$_2$**C**H$_3$), 36.4 (CH$_2$), 36.0 (CH).

ESI-HRMS (*m/z*): calc. for $[C_{21}H_{19}{}^{79}BrNO_6]^-$ 460.0401, found 460.0408 $[M-H]^-$.

ESI-LCMS (*m/z*): calc. for $[C_{21}H_{19}{}^{79}BrNO_6]^-$ 460.0, found 460.1 $[M-H]^-$ (97.115%).

Synthesis of target acids

Synthesis of 3-(3,4-dimethoxyphenyl)-3-(6-fluoro-4-hydroxy-2-oxo-1,2-dihydroquinolin-3-yl)propanoic acid (11a)

Method 1:

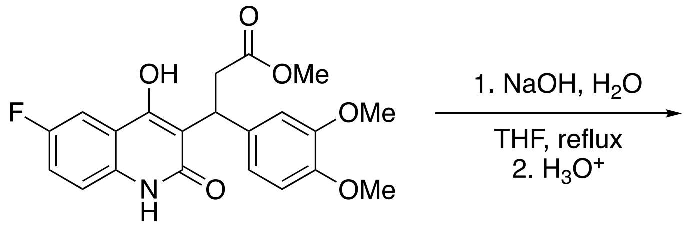

0.60 g (1.5 mmol) of the starting methyl ester **10a**, 25 mL THF and 25 mL 5 M NaOH solution were placed in a 100 mL round-bottomed flask equipped with a magnetic stirrer and a reflux condenser. The reaction mixture was stirred vigorously at boiling (oil bath temperature 100 °C) for several days. The extent of reaction product formation was monitored by TLC. The cooled reaction mixture was then extracted with saturated $NaHCO_3$ solution. The organic layer was separated and the aqueous layer was acidified with concentrated HCl to pH 1–2. The resulting suspension was extracted with EtOAc. The organic phase was dried over anhydrous Na_2SO_4 and concentrated under reduced pressure. As a result, the pure product was obtained as a powder weighing 0.55 g in 95% yield.

Method 2:

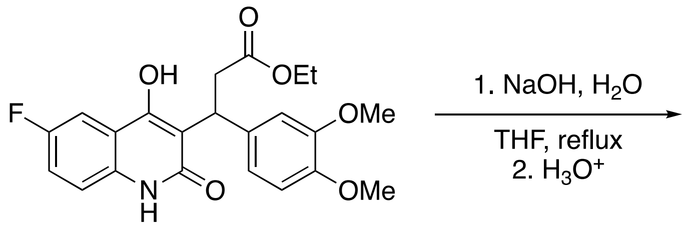

0.70 g (1.7 mmol) of the starting ethyl ether **9a**, 25 mL THF and 25 mL 5 M NaOH solution were placed in a 100 mL round-bottomed flask equipped with a magnetic stirrer and a reflux condenser. The reaction mixture was stirred vigorously at boiling (oil bath temperature 100 °C) for several days. The extent of reaction product formation was monitored by TLC. The cooled reaction mixture was then extracted with saturated $NaHCO_3$ solution. The organic layer was separated and the aqueous layer was acidified with concentrated HCl to pH 1–2. The resulting suspension was extracted with EtOAc. The organic phase was dried over anhydrous Na_2SO_4 and concentrated under reduced pressure. As a result, the pure product was obtained as a powder weighing 0.56 g in 85% yield.

^{1}H NMR spectrum (400 MHz, DMSO-d_6) δ 11.98 (s, 1H, CO_2H), 11.33 (s, 1H, NH), 10.43 (s, 1H, OH), 7.70 (dd, J = 10.2, 2.8 Hz, 1H, H_{Ar}), 7.34 (td, J = 8.7, 2.8 Hz, 1H, H_{Ar}), 7.25 (dd, J = 9.0, 4.9 Hz, 1H, H_{Ar}), 7.06 (d, J = 2.0 Hz, 1H, H_{Ar}), 6.90 (dd, J = 8.4, 2.1 Hz, 1H, H_{Ar}), 6.79 (d, J = 8.3 Hz,

1H, H_{Ar}), 4.90 (t, J = 7.8 Hz, 1H, CH), 3.69 (s, 3H, OMe), 3.68 (s, 3H, OMe), 3.21 (qd, J = 15.9, 7.8 Hz, 2H, CH_2).

^{13}C NMR spectrum (100 MHz, DMSO-d_6) δ 173.8 (CO_2H), 162.9 (C(O)NH), 157.0 (d, J = 2.2 Hz, **C**(OH)=C), 156.8 (d, J = 236.6 Hz, $C_{Ar}F$), 148.2 (**C_{Ar}**OCH_3), 147.1 (**C_{Ar}**OCH_3), 136.0 (C_{Ar}), 134.4 (C_{Ar}), 119.8 (C_{Ar}), 118.0 (d, J = 23.9 Hz, C_{Ar}), 116.8 (C_{Ar}), 116.2 (d, J = 8.2 Hz, C_{Ar}), 115.4 (C(OH)=**C**), 112.1 (d, J = 8.9 Hz, C_{Ar}), 111.6 (C_{Ar}), 108.1 (d, J = 25.0 Hz, C_{Ar}), 55.6 (OCH_3), 55.5 (OCH_3), 36.8 (CH_2), 36.1 (**C**H).

ESI-HRMS (*m/z*): calc. for $[C_{20}H_{17}FNO_6]^-$ 386.1045, found 386.1048 $[M-H]^-$.

ESI-LCMS (*m/z*): calc. for $[C_{20}H_{17}FNO_6]^-$ 386.1, found 386.2 $[M-H]^-$ (98.392%).

Synthesis of 3-(6-chloro-4-hydroxy-2-oxo-1,2-dihydroquinolin-3-yl)-3-(3,4-dimethoxyphenyl)propanoic acid (11b)

Method 1:

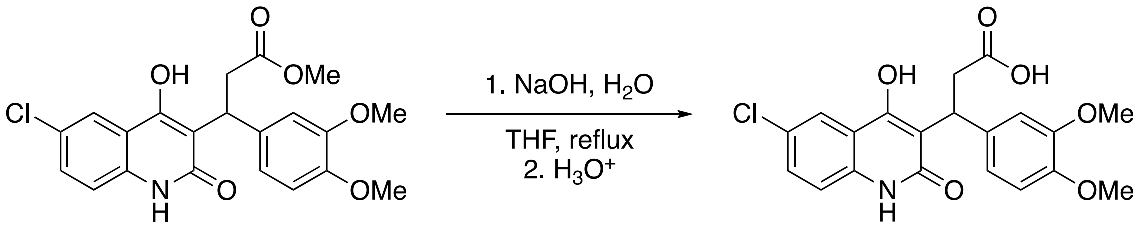

In a 100 mL round-bottomed flask equipped with a magnetic stirrer and a reflux condenser, 0.500 g (1.2 mmol) of the starting methyl ester **10b**, 25 mL THF and 25 mL 5 M NaOH solution were placed. The reaction mixture was stirred vigorously at boiling (oil bath temperature 100 °C) for several days. The extent of reaction product formation was monitored by TLC. The cooled reaction mixture was then extracted with saturated $NaHCO_3$ solution. The organic layer was separated and the aqueous layer was acidified with concentrated HCl to pH 1–2. The resulting suspension was extracted with EtOAc. The organic phase was dried over anhydrous Na_2SO_4 and concentrated under reduced pressure. As a result, the pure product was obtained as a powder weighing 0.435 g in 90% yield.

Method 2:

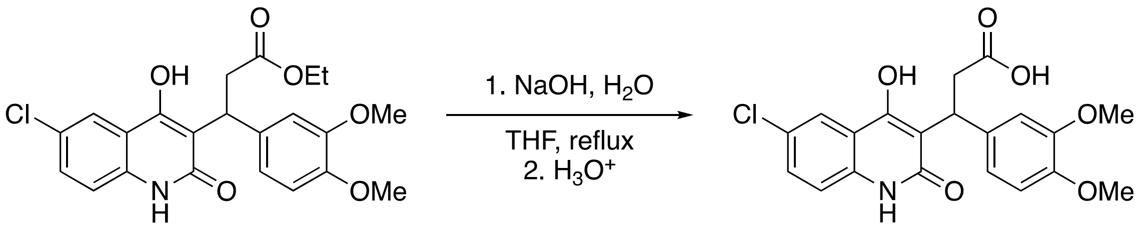

In a 100 mL round-bottomed flask equipped with a magnetic stirrer and a reflux condenser, 0.432 g (1 mmol) of the starting ethyl ether **9b**, 25 mL THF and 25 mL 5 M NaOH solution were placed. The

reaction mixture was stirred vigorously at boiling (oil bath temperature 90 °C) for several days. The extent of reaction product formation was monitored by TLC. The cooled reaction mixture was then extracted with saturated $NaHCO_3$ solution. The organic layer was separated and the aqueous layer was acidified with concentrated HCl to pH 1–2. The resulting suspension was extracted with EtOAc. The organic phase was dried over anhydrous Na_2SO_4 and concentrated under reduced pressure. As a result, a pure product was obtained weighing 0.390 g in 97% yield.

^{1}H NMR spectrum (400 MHz, DMSO-d_6) δ 11.91 (s, 1H, CO_2H), 11.39 (s, 1H, NH), 10.55 (s, 1H, OH), 7.95 (d, J = 2.3 Hz, 1H, H_{Ar}), 7.48 (dd, J = 8.8, 2.3 Hz, 1H, H_{Ar}), 7.24 (d, J = 8.8 Hz, 1H, H_{Ar}), 7.04 (d, J = 2.0 Hz, 1H, H_{Ar}), 6.89 (dd, J = 8.4, 2.0 Hz, 1H, H_{Ar}), 6.79 (d, J = 8.4 Hz, 1H, H_{Ar}), 4.89 (t, J = 7.7 Hz, 1H, CH), 3.68 (s, 3H, OMe), 3.68 (s, 3H, OMe), 3.29 – 3.11 (m, 2H, CH_2).

^{13}C NMR spectrum (100 MHz, DMSO-d_6) δ 173.8 (CO_2H), 162.9 (C(O)NH), 156.6 (**C**(OH)=C), 148.2 (**C_{Ar}**OCH_3), 147.1 (**C_{Ar}**OCH_3), 136.4 (C_{Ar}), 135.9 (C_{Ar}), 129.9 (C_{Ar}), 125.1 (C_{Ar}), 122.0 (C_{Ar}), 119.7 (C_{Ar}), 116.8 (C_{Ar}), 116.6 (C_{Ar}), 115.6 (C(OH)=**C**), 112.0 (C_{Ar}), 111.5 (C_{Ar}), 55.5 (OCH_3), 55.4 (OCH_3), 36.8 (CH_2), 36.0 (**C**HCH_2).

ESI-HRMS (*m/z*): calc. for $[C_{20}H_{19}ClNO_6]^+$ 404.0895, found 404.0899 $[M+H]^+$.

ESI-LCMS (*m/z*): calc. for $[C_{20}H_{19}ClNO_6]^+$ 404.09, found 404.15 $[M+H]^+$ (100%).

Synthesis of 3-(6-bromo-4-hydroxy-2-oxo-1,2-dihydroquinolin-3-yl)-3-(3,4-dimethoxyphenyl)propanoic acid (11c)

Method 1:

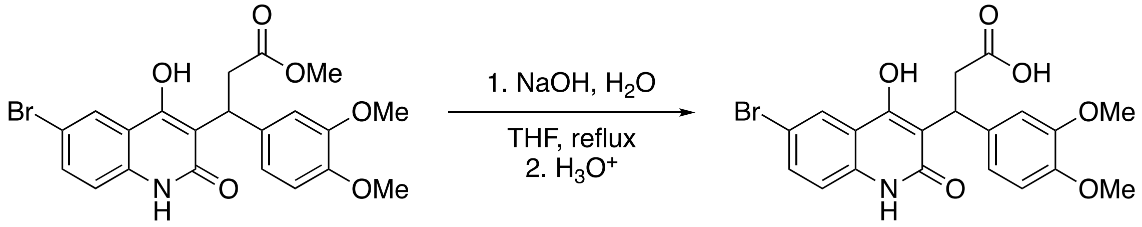

0.70 g (1.5 mmol) of the starting methyl ester **10c**, 25 mL THF and 25 mL 5 M NaOH solution were placed in a 100 mL round-bottomed flask equipped with a magnetic stirrer and a reflux condenser. The reaction mixture was stirred vigorously at boiling (oil bath temperature 100 °C) for several days. The extent of reaction product formation was monitored by TLC. The cooled reaction mixture was then extracted with saturated $NaHCO_3$ solution. The organic layer was separated and the aqueous layer was acidified with concentrated HCl to pH 1–2. The resulting suspension was extracted with EtOAc. The organic phase was dried over anhydrous Na_2SO_4 and concentrated under reduced pressure. As a result, the pure product was obtained as a powder weighing 0.65 g in 96% yield.

Method 2:

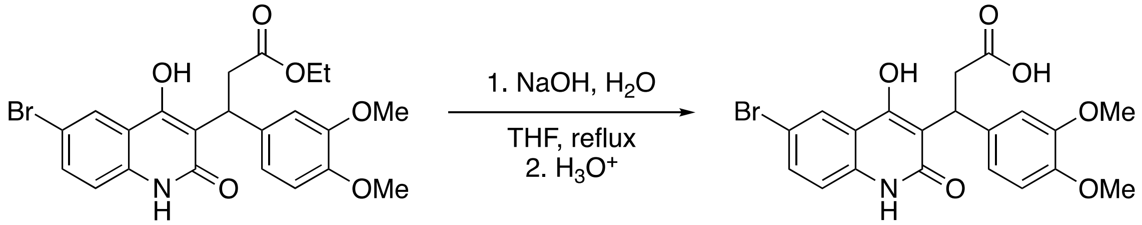

0.800 g (1.7 mmol) of the starting ethyl ether **9c**, 25 mL THF and 25 mL 5 M NaOH solution were placed in a 100 mL round-bottomed flask equipped with a magnetic stirrer and a reflux condenser. The reaction mixture was stirred vigorously at boiling (oil bath temperature 90 °C) for several days. The extent of reaction product formation was monitored by TLC. The cooled reaction mixture was then extracted with saturated $NaHCO_3$ solution. The organic layer was separated and the aqueous layer was acidified with concentrated HCl to pH 1–2. The resulting suspension was extracted with EtOAc. The organic phase was dried over anhydrous Na_2SO_4 and concentrated under reduced pressure. As a result, the pure product was obtained as a powder weighing 0.678 g in 90% yield.

^{1}H NMR spectrum (400 MHz, DMSO-d_6) δ 11.38 (s, 1H, NH), 8.09 (d, J = 2.2 Hz, 1H, H_{Ar}), 7.58 (dd, J = 8.7, 2.2 Hz, 1H, H_{Ar}), 7.18 (d, J = 8.7 Hz, 1H, H_{Ar}), 7.04 (d, J = 2.1 Hz, 1H, H_{Ar}), 6.88 (dd, J = 8.4, 2.0 Hz, 1H, H_{Ar}), 6.79 (d, J = 8.4 Hz, 1H, H_{Ar}), 4.89 (t, J = 7.7 Hz, 1H, CH), 3.68 (s, 3H, OMe), 3.68 (s, 3H, OMe), 3.20 (qd, J = 15.9, 7.7 Hz, 2H, CH_2).

^{13}C NMR spectrum (100 MHz, DMSO-d_6) δ 173.8 (CO_2H), 162.9 (C(O)NH), 156.7 (**C**(OH)=C), 148.2 (**C$_{Ar}$**OCH_3), 147.1 (**C$_{Ar}$**OCH_3), 136.7 (C_{Ar}), 135.9 (C_{Ar}), 132.6 (C_{Ar}), 125.0 (C_{Ar}), 119.7 (C_{Ar}), 117.2 (C_{Ar}), 117.1 (C_{Ar}), 115.4 (C_{Ar}), 112.8 (C(OH)=**C**), 112.0 (C_{Ar}), 111.5 (C_{Ar}), 55.6 (OCH_3), 55.4 (OCH_3), 36.9 (CH_2), 36.0 (CH).

ESI-HRMS (*m/z*): calc. for $[C_{20}H_{17}{}^{79}BrNO_6]^-$ 446.0245, found 446.0250 $[M-H]^-$.

ESI-LCMS (*m/z*): calc. for $[C_{20}H_{17}{}^{81}BrNO_6]^-$ 448.0, found 448.1 $[M-H]^-$ (98.916%).

IR ν_{max} (cm^{-1}, KBr): 2826,55 (COO−H, st, H-bonded group); 1668,48 (C=O, st, H-bonded, intramolecular H-bond); 1600,48 (COO^-, st as); 1253,22, 1140 (C−O, st, C(OH)=C).

Synthesis of target isopropyl esters

Synthesis of isopropyl 3-(3,4-dimethoxyphenyl)-3-(6-fluoro-4-hydroxy-2-oxo-1,2-dihydroquinolin-3-yl)propanoate (12a)

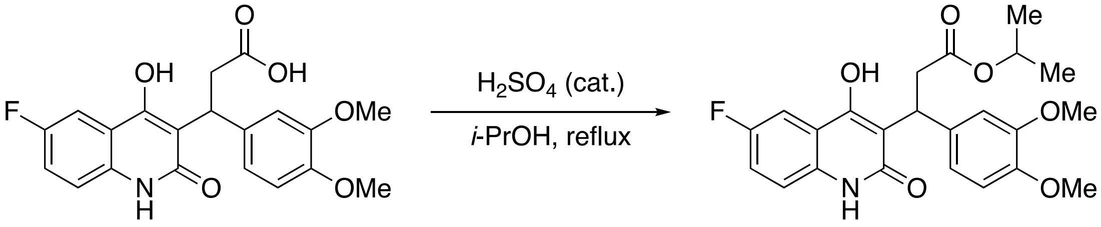

In a 100 mL round-bottomed flask equipped with a magnetic stirrer and a reflux condenser, 0.533 g (1.4 mmol) of the starting acid **11a** and 30 mL of isopropanol were placed. The resulting mixture was stirred vigorously, gradually increasing the temperature. 500 µL of concentrated H_2SO_4 was added to the resulting solution, after which instantaneous dissolution of the starting substance was observed. The reaction mixture was stirred vigorously at boiling (temperature in the oil bath 120 °C). The degree of formation of the reaction product was monitored by TLC. After a few hours the synthesis was stopped. The reaction mixture was then dissolved in EtOAc. The resulting mixture was washed with saturated $NaHCO_3$ solution, 1 M HCl solution and saturated NaCl solution. The organic phase was dried over anhydrous Na_2SO_4, concentrated under reduced pressure, and dried. As a result, the pure product was obtained as a grey powder weighing 0.429 g in 71% yield.

^{1}H NMR spectrum (400 MHz, DMSO-*d*$_6$) δ 11.35 (s, 1H, NH), 10.46 (s, 1H, OH), 7.70 (dd, *J* = 10.2, 2.8 Hz, 1H, H_{Ar}), 7.34 (td, *J* = 8.7, 2.8 Hz, 1H, H_{Ar}), 7.25 (dd, *J* = 9.0, 4.9 Hz, 1H, H_{Ar}), 7.04 (d, *J* = 2.0 Hz, 1H, H_{Ar}), 6.89 (dd, *J* = 8.4, 2.0 Hz, 1H, H_{Ar}), 6.79 (d, *J* = 8.4 Hz, 1H, H_{Ar}), 4.90 (t, *J* = 8.0 Hz, 1H, $CHCH_2$), 4.75 (sept, *J* = 6.3 Hz, 1H, $CH(CH_3)_2$), 3.69 (s, 3H, OMe), 3.68 (s, 3H, OMe), 3.31 (dd, *J* = 15.2, 8.7 Hz, 1H, CH_2), 3.17 (dd, *J* = 15.2, 7.5 Hz, 1H, CH_2), 1.02 (t, *J* = 6.1 Hz, 6H, $CH(CH_3)_2$).

^{13}C NMR spectrum (100 MHz, DMSO-*d*$_6$) δ 171.6 (**C**O_2Pr^i), 162.8 (C(O)NH), 157.0 (d, *J* = 2.8 Hz, **C**(OH)=C), 156.8 (d, *J* = 236.8 Hz, $C_{Ar}F$), 148.2 (**C$_{Ar}$**OCH_3), 147.2 (**C$_{Ar}$**OCH_3), 135.6 (C_{Ar}), 134.4 (C_{Ar}), 119.8 (C_{Ar}), 118.1 (d, *J* = 24.1 Hz, C_{Ar}), 116.8 (d, *J* = 7.9 Hz, C_{Ar}), 116.0 (d, *J* = 8.2 Hz, C_{Ar}), 114.9 (C(OH)=**C**), 111.9 (C_{Ar}), 111.5 (C_{Ar}), 107.9 (d, *J* = 24.8 Hz, C_{Ar}), 66.7 (**C**H$(CH_3)_2$), 55.5 (OCH_3), 55.4 (OCH_3), 36.9 (CH_2), 36.2 (**C**HCH_2), 21.5 (**C**H$_3$CH**C**H$_3$).

ESI-HRMS (*m/z*): calc. for $[C_{23}H_{23}FNO_6]^-$ 428.1515, found 428.1518 $[M-H]^-$.

ESI-LCMS (*m/z*): calc. for $[C_{23}H_{25}FNO_6]^+$ 430.2, found 430.2 $[M+H]^+$ (95.469%).

Synthesis of isopropyl 3-(6-chloro-4-hydroxy-2-oxo-1,2-dihydroquinolin-3-yl)-3-(3,4-dimethoxyphenyl)propanoate (12b)

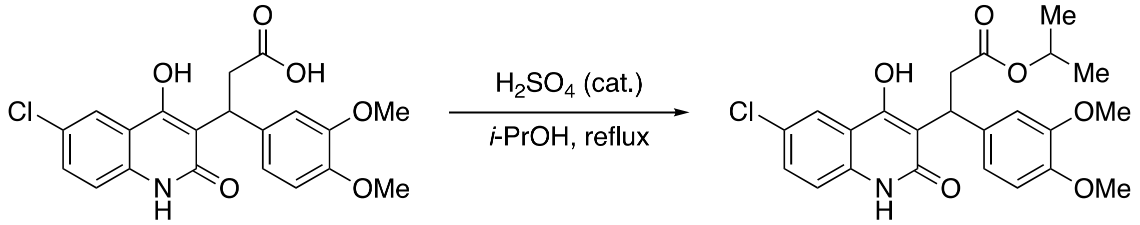

0.101 g (0.25 mmol) of the starting acid **11b** and 30 mL of isopropanol were placed in a 100 mL round-bottomed flask equipped with a magnetic stirrer and a reflux condenser. The resulting mixture was stirred vigorously, gradually increasing the temperature, until complete dissolution of the starting acid. 300 μL of concentrated H_2SO_4 was added to the resulting solution. The reaction mixture was stirred vigorously at boiling (temperature in the oil bath 130 °C). The extent of reaction product formation was monitored by TLC. After a few hours the synthesis was stopped. The reaction mixture was then dissolved in EtOAc, washed with saturated $NaHCO_3$ solution, 1 M HCl solution and saturated NaCl solution. The resulting organic phase was dried over anhydrous Na_2SO_4, concentrated under reduced pressure, and dried. As a result, the pure product was obtained as a powder weighing 0.102 g in 92% yield.

^{1}H NMR spectrum (400 MHz, DMSO-*d*$_6$) δ 11.44 (s, 1H, NH), 10.55 (s, 1H, OH), 7.97 (d, *J* = 2.2 Hz, 1H, H_{Ar}), 7.49 (dd, *J* = 8.8, 2.2 Hz, 1H, H_{Ar}), 7.25 (d, *J* = 8.8 Hz, 1H, H_{Ar}), 7.05 (d, *J* = 1.7 Hz, 1H, H_{Ar}), 6.89 (dd, *J* = 8.4, 1.7 Hz, 1H, H_{Ar}), 6.80 (d, *J* = 8.4 Hz, 1H, H_{Ar}), 4.91 (t, *J* = 8.0 Hz, 1H, C**H**CH_2), 4.76 (sept, *J* = 6.2 Hz, 1H), 3.69 (s, 3H, OMe), 3.68 (s, 3H, OMe), 3.33 – 3.28 (m, 1H, CH_2), 3.18 (dd, *J* = 15.2, 7.4 Hz, 1H, CH_2), 1.03 (t, *J* = 5.8 Hz, 6H, CH(C**H**$_3$)$_2$).

^{13}C NMR spectrum (100 MHz, DMSO-*d*$_6$) δ 171.6 (**C**O_2Pr^i), 162.9 (C(O)NH), 156.7 (**C**(OH)=C), 148.2 (**C$_{Ar}$**OCH_3), 147.2 (**C$_{Ar}$**OCH_3), 136.4 (C_{Ar}), 135.5 (C_{Ar}), 130.0 (C_{Ar}), 125.1 (C_{Ar}), 122.1 (C_{Ar}), 119.8 (C_{Ar}), 116.8 (C_{Ar}), 116.6 (C_{Ar}), 115.0 (C(OH)=**C**), 111.9 (C_{Ar}), 111.5 (C_{Ar}), 66.7 (**C**H(CH_3)$_2$), 55.7 – 55.3 (2×OCH_3), 36.8 (CH_2), 36.2 (**C**HCH_2), 21.5 – 21.4 (**C**H_3CH**C**H_3).

ESI-HRMS (*m/z*): calc. for $[C_{23}H_{23}ClNO_6]^-$ 444.1219, found 444.1226 $[M-H]^-$.

ESI-LCMS (*m/z*): calc. for $[C_{23}H_{23}ClNO_6]^-$ 444.12, found 444.25 $[M-H]^-$ (100%).

Synthesis of isopropyl 3-(6-bromo-4-hydroxy-2-oxo-1,2-dihydroquinolin-3-yl)-3-(3,4-dimethoxyphenyl)propanoate (12c)

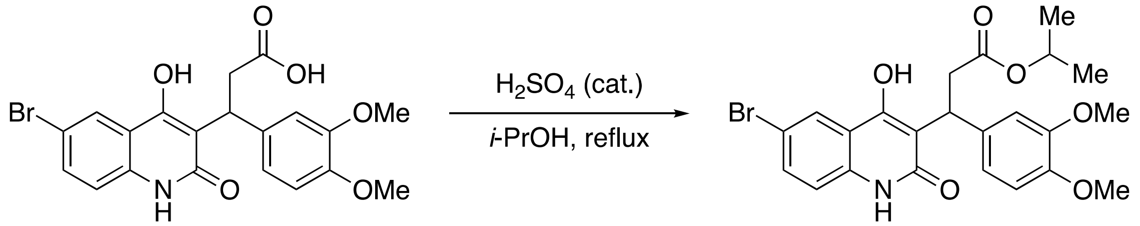

In a 100 mL round-bottomed flask equipped with a magnetic stirrer and a reflux condenser, 0.189 g (0.4 mmol) of the starting acid **11c** and 30 mL of isopropanol were placed. The resulting mixture was stirred vigorously, gradually increasing the temperature, until complete dissolution of the starting acid. 300 µL of concentrated H_2SO_4 was added to the resulting solution. The reaction mixture was stirred vigorously at boiling (temperature in the oil bath 130 °C). The extent of reaction product formation was monitored by TLC. After a few hours the synthesis was stopped. The reaction mixture was then dissolved in EtOAc, washed with saturated $NaHCO_3$ solution, 1 M HCl solution and saturated NaCl solution. The resulting organic phase was dried over anhydrous Na_2SO_4, concentrated under reduced pressure, and dried. As a result, the pure product was obtained as a powder weighing 0.203 g in 98% yield.

^{1}H NMR spectrum (400 MHz, DMSO-*d*$_6$) δ 11.42 (s, 1H, NH), 10.53 (s, 1H, OH), 8.10 (d, *J* = 2.2 Hz, 1H, H_{Ar}), 7.58 (dd, *J* = 8.7, 2.1 Hz, 1H, H_{Ar}), 7.18 (d, *J* = 8.7 Hz, 1H, H_{Ar}), 7.03 (d, *J* = 2.1 Hz, 1H, H_{Ar}), 6.88 (dd, *J* = 8.3, 2.0 Hz, 1H, H_{Ar}), 6.79 (d, *J* = 8.4 Hz, 1H, H_{Ar}), 4.89 (t, *J* = 8.0 Hz, 1H, **C**HCH_2), 4.75 (sept, *J* = 6.4 Hz, 1H, C**H**$(CH_3)_2$), 3.69 (s, 3H, OMe), 3.68 (s, 3H, OMe), 3.30 (dd, *J* = 15.1, 8.6 Hz, 1H, CH_2), 3.17 (dd, *J* = 15.2, 7.4 Hz, 1H, CH_2), 1.02 (dd, *J* = 6.2, 5.1 Hz, 6H, CH(C**H**$_3$)$_2$).

^{13}C NMR spectrum (100 MHz, DMSO-*d*$_6$) δ 171.6 (**C**O_2Pr^i), 162.8 (C(O)NH), 156.6 (**C**(OH)=C), 148.2 (**C$_{Ar}$**OCH_3), 147.2 (**C$_{Ar}$**OCH_3), 136.7 (C_{Ar}), 135.5 (C_{Ar}), 132.7 (C_{Ar}), 125.0 (C_{Ar}), 119.7 (C_{Ar}), 117.1 (C_{Ar}), 117.0 (C_{Ar}), 115.0 (C_{Ar}), 112.8 (C(OH)=**C**), 111.9 (C_{Ar}), 111.5 (C_{Ar}), 66.7 (**C**H$(CH_3)_2$), 55.5 (OCH_3), 55.4 (OCH_3), 36.8 (CH_2), 36.1 (**C**HCH_2), 21.5 (**C**H_3CH**C**H_3).

ESI-HRMS (*m/z*): calc. for $[C_{23}H_{23}{}^{79}BrNO_6]^-$ 488.0714, found 488.0720 $[M-H]^-$.

ESI-LCMS (*m/z*): calc. for $[C_{23}H_{23}{}^{79}BrNO_6]^-$ 488.07, found 488.15 $[M-H]^-$ (93.84%).

Synthesis of target cyclohexyl esters

Synthesis of cyclohexyl 3-(3,4-dimethoxyphenyl)-3-(6-fluoro-4-hydroxy-2-oxo-1,2-dihydroquinolin-3-yl)propanoate (13a)

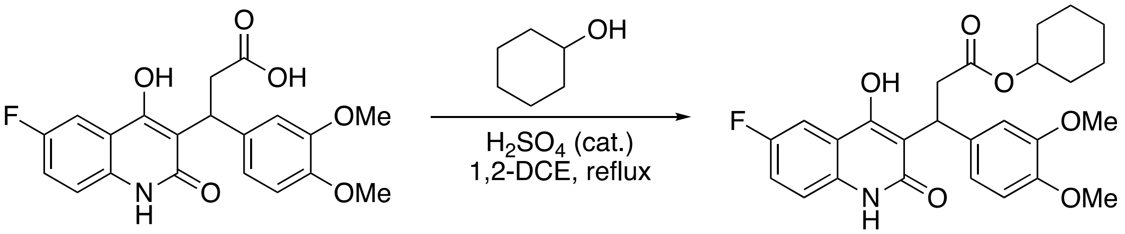

0.156 g (0.4 mmol) of the starting acid **11a**, 25 mL of 1,2-dichloroethane, 5 mL of cyclohexanol and 50 μL of concentrated H_2SO_4 were placed in a 100 mL round-bottomed flask equipped with a magnetic stirrer and a reflux condenser. The resulting mixture, stirring vigorously, was gradually heated to boiling (oil bath temperature ≈100 °C). The extent of reaction product formation was monitored by TLC (CH_2Cl_2/MeOH (20:1)). After a few hours, the synthesis was stopped. The solvent was distilled off at reduced pressure on a rotary evaporator. The residue was dissolved in EtOAc, the resulting solution was washed with saturated $NaHCO_3$ solution and water. Then at reduced pressure at a rotary evaporator with a heated water bath the organic phase was repeatedly re-evaporated first in a mixture with water and then with EtOAc, thus distilling off cyclohexanol. The mixture remaining in the flask was extracted with EtOAc, the resulting organic solution was washed with water and saturated NaCl solution. The obtained organic phase was dried over anhydrous Na_2SO_4, concentrated under reduced pressure, dried. As a result, a pure product with mass 0.123 g in 65% yield was obtained.

^{1}H NMR spectrum (400 MHz, DMSO-d_6) δ 11.38 (s, 1H), 10.51 (s, 1H, OH), 7.72 (dd, J = 10.2, 2.7 Hz, 1H, H_{Ar}), 7.35 (td, J = 8.7, 2.7 Hz, 1H, H_{Ar}), 7.26 (dd, J = 9.0, 5.0 Hz, 1H, H_{Ar}), 7.06 (d, J = 1.8 Hz, 1H, H_{Ar}), 6.91 (dd, J = 8.4, 1.7 Hz, 1H, H_{Ar}), 6.80 (d, J = 8.4 Hz, 1H, H_{Ar}), 4.93 (t, J = 8.0 Hz, 1H, C**H**CH_2), 4.55 (sept, J = 3.5 Hz, 1H, CO_2CH), 3.69 (s, 3H, OMe), 3.68 (s, 3H, OMe), 3.34 (dd, J = 15.2, 8.6 Hz, 1H, CH_2), 3.21 (dd, J = 15.2, 7.5 Hz, 1H, CH_2), 1.68 – 1.06 (m, 10H, H_{Cy}).

^{13}C NMR spectrum (100 MHz, DMSO-d_6) δ 171.5 (**C**O_2Cy), 162.8 (C(O)NH), 157.0 (d, J = 2.7 Hz, **C**(OH)=C), 156.7 (d, J = 236.6 Hz, C_{Ar}F), 148.2 (**C_{Ar}**OCH_3), 147.2 (**C_{Ar}**OCH_3), 135.6 (C_{Ar}), 134.4 (C_{Ar}), 119.7 (C_{Ar}), 118.1 (d, J = 24.5 Hz, C_{Ar}), 116.7 (d, J = 7.7 Hz, C_{Ar}), 116.1 (d, J = 8.2 Hz, C_{Ar}), 114.9 (C(OH)=**C**), 111.9 (C_{Ar}), 111.5 (C_{Ar}), 107.9 (d, J = 24.3 Hz, C_{Ar}), 71.3 (CO_2**C**H), 55.5 (OCH_3), 55.4 (OCH_3), 36.9 (CH**C**H_2), 36.2 (**C**HCH_2), 30.9 (2×$CH_{2,\,Cy}$), 24.9 ($CH_{2,\,Cy}$), 22.9 (2×$CH_{2,\,Cy}$).

^{19}F NMR spectrum (376 MHz, DMSO-d_6) δ −121.32 (td, J = 9.4, 5.2 Hz).

ESI-HRMS (*m/z*): calc. for $[C_{26}H_{27}FNO_6]^-$ 468.1828, found 468.1831 $[M-H]^-$.

ESI-LCMS (*m/z*): calc. for $[C_{26}H_{27}FNO_6]^-$ 468.18, found 468.25 $[M-H]^-$ (97.602%).

Synthesis of cyclohexyl 3-(6-chloro-4-hydroxy-2-oxo-1,2-dihydroquinolin-3-yl)-3-(3,4-dimethoxyphenyl)propanoate (13b)

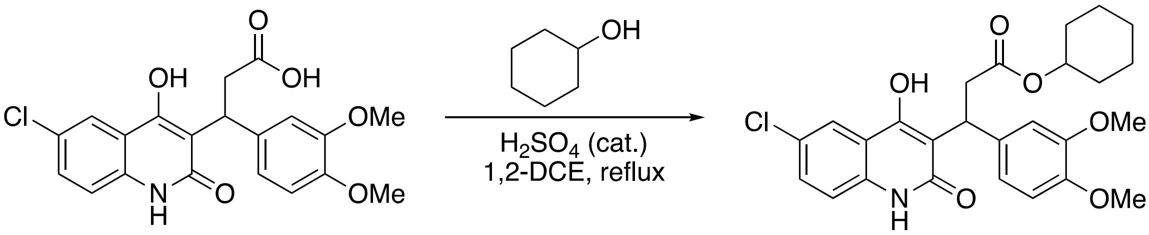

0.212 g (0.52 mmol) of the starting acid **11b**, 25 mL of 1,2-dichloroethane, 5 mL of cyclohexanol and 50 μL of concentrated H_2SO_4 were placed in a 100 mL round-bottomed flask equipped with a magnetic stirrer and a reflux condenser. The resulting mixture, stirring vigorously, was gradually heated to boiling (oil bath temperature ≈100 °C). The extent of reaction product formation was monitored by TLC (CH_2Cl_2/MeOH (20:1)). After a few hours, the synthesis was stopped. The solvent was distilled off at reduced pressure on a rotary evaporator. The residue was dissolved in EtOAc, the resulting solution was washed with saturated $NaHCO_3$ solution and water. Then at reduced pressure at a rotary evaporator with a heated water bath the organic phase was repeatedly re-evaporated first in a mixture with water and then with EtOAc, thus distilling off cyclohexanol. The mixture remaining in the flask was extracted with EtOAc, the resulting organic solution was washed with water and saturated NaCl solution. The obtained organic phase was dried over anhydrous Na_2SO_4, concentrated under reduced pressure, dried. As a result, a pure product was obtained with a mass of 0.205 g in 81% yield.

^{1}H NMR spectrum (400 MHz, DMSO-d_6) δ 11.44 (s, 1H, NH), 10.57 (s, 1H, OH), 7.98 (d, J = 2.3 Hz, 1H, H_{Ar}), 7.48 (dd, J = 8.8, 2.3 Hz, 1H, H_{Ar}), 7.25 (d, J = 8.7 Hz, 1H, H_{Ar}), 7.05 (d, J = 2.1 Hz, 1H, H_{Ar}), 6.90 (dd, J = 8.4, 2.0 Hz, 1H, H_{Ar}), 6.80 (d, J = 8.4 Hz, 1H, H_{Ar}), 4.92 (t, J = 8.0 Hz, 1H, C**H**CH_2), 4.55 (dt, J = 8.7, 4.5 Hz, 1H, CO_2CH), 3.69 (s, 3H, OMe), 3.68 (s, 3H, OMe), 3.33 (dd, J = 15.2, 8.5 Hz, 1H, CH_2), 3.21 (dd, J = 15.2, 7.5 Hz, 1H, CH_2), 1.64 – 1.08 (m, 10H, H_{Cy}).

^{13}C NMR spectrum (100 MHz, DMSO-d_6) δ 171.5 (**C**O_2Cy), 162.9 (C(O)NH), 156.7 (**C**(OH)=C), 148.2 (**C$_{Ar}$**OCH_3), 147.2 (**C$_{Ar}$**OCH_3), 136.4 (C_{Ar}), 135.5 (C_{Ar}), 130.0 (C_{Ar}), 125.1 (C_{Ar}), 122.0 (C_{Ar}), 119.7 (C_{Ar}), 116.8 (C_{Ar}), 116.6 (C_{Ar}), 115.0 (C(OH)=**C**), 111.9 (C_{Ar}), 111.5 (C_{Ar}), 71.3 (CO_2**C**H), 55.5 (OCH_3), 55.4 (OCH_3), 36.9 (CH**C**H_2), 36.2 (**C**HCH_2), 30.9 (2×$CH_{2,Cy}$), 24.9 ($CH_{2,Cy}$), 22.9 (2×$CH_{2,Cy}$).

ESI-HRMS (*m/z*): calc. for $[C_{26}H_{27}ClNO_6]^-$ 484.1532, found 484.1540 $[M-H]^-$.

ESI-LCMS (*m/z*): calc. for $[C_{26}H_{27}ClNO_6]^-$ 484.15, found 484.25 $[M-H]^-$ (81.113%).

Synthesis of cyclohexyl 3-(6-bromo-4-hydroxy-2-oxo-1,2-dihydroquinolin-3-yl)-3-(3,4-dimethoxyphenyl)propanoate (13c)

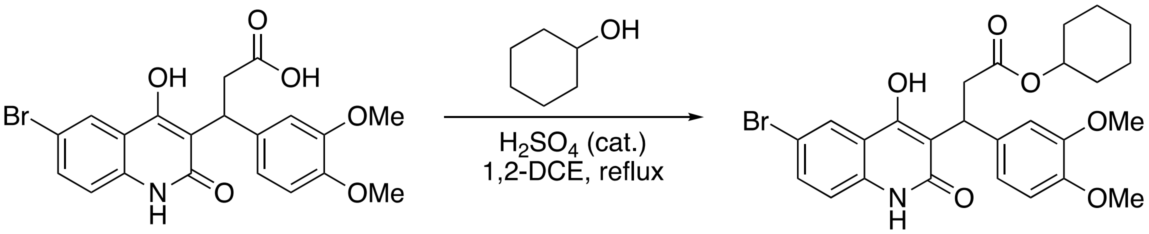

0.224 g (0.5 mmol) of the starting acid **11c**, 25 mL of 1,2-dichloroethane, 5 mL of cyclohexanol and 50 µL of concentrated H_2SO_4 were placed in a 100 mL round-bottomed flask equipped with a magnetic stirrer and a reflux condenser. The resulting mixture, stirring vigorously, was gradually heated to boiling (oil bath temperature ≈100 °C). The extent of reaction product formation was monitored by TLC (CH_2Cl_2/MeOH (20:1)). After a few hours, the synthesis was stopped. The solvent was distilled off at reduced pressure on a rotary evaporator. The residue was dissolved in EtOAc, the resulting solution was washed with saturated $NaHCO_3$ solution and water. Then at reduced pressure at a rotary evaporator with a heated water bath the organic phase was repeatedly re-evaporated first in a mixture with water and then with EtOAc, thus distilling off cyclohexanol. The mixture remaining in the flask was extracted with EtOAc, the resulting organic solution was washed with water and saturated NaCl solution. The obtained organic phase was dried over anhydrous Na_2SO_4, concentrated under reduced pressure, dried. As a result, a pure product was obtained with mass 0.191 g in 72% yield.

^{1}H NMR spectrum (400 MHz, DMSO-d_6) δ 11.47 (s, 1H, NH), 10.72 (s, 1H, OH), 8.25 (d, J = 2.2 Hz, 1H, H_{Ar}), 7.58 (dd, J = 8.7, 2.2 Hz, 1H, H_{Ar}), 7.22 (d, J = 8.8 Hz, 1H, H_{Ar}), 7.05 (d, J = 2.0 Hz, 1H, H_{Ar}), 6.89 (dd, J = 8.3, 2.1 Hz, 1H, H_{Ar}), 6.79 (d, J = 8.4 Hz, 1H, H_{Ar}), 4.91 (t, J = 8.0 Hz, 1H, C**H**CH_2), 4.54 (tt, J = 8.2, 3.3 Hz, 1H, CO_2CH), 3.69 (s, 3H, OMe), 3.68 (s, 3H, OMe), 3.25 (qd, J = 15.3, 8.1 Hz, 2H, CH_2CO_2), 1.66 – 1.07 (m, 10H, H_{Cy}).

^{13}C NMR spectrum (100 MHz, DMSO-d_6) δ 171.5 (**C**O_2Cy), 162.9 (C(O)NH), 156.6 (**C**(OH)=C), 148.2 (**C**$_{\mathbf{Ar}}$$OCH_3$), 147.2 (**C**$_{\mathbf{Ar}}$$OCH_3$), 136.7 ($C_{Ar}$), 135.5 ($C_{Ar}$), 132.6 ($C_{Ar}$), 125.0 ($C_{Ar}$), 119.7 ($C_{Ar}$), 117.1 ($C_{Ar}$), 117.0 ($C_{Ar}$), 115.0 ($C_{Ar}$), 112.8 (C(OH)=**C**), 111.9 (C_{Ar}), 111.5 (C_{Ar}), 71.4 (CO_2**C**H), 55.5 (OCH_3), 55.4 (OCH_3), 36.9 (CH**C**H_2), 36.2 (**C**HCH_2), 30.9 (2×$CH_{2,\,Cy}$), 24.9 ($CH_{2,\,Cy}$), 23.0 (2×$CH_{2,\,Cy}$).

ESI-HRMS (*m/z*): calc. for $[C_{26}H_{27}{}^{81}BrNO_6]^-$ 530.1006, found 530.1014 $[M{-}H]^-$.

ESI-LCMS (*m/z*): calc. for $[C_{26}H_{27}{}^{79}BrNO_6]^-$ 528.1, found 528.2 $[M{-}H]^-$ (96.787%).

Synthesis of target pyranoquinolines

Synthesis of 4-(3,4-dimethoxyphenyl)-9-fluoro-4,6-dihydro-2*H*-pyrano[3,2-*c*]quinoline-2,5(3*H*)-dione (14a)

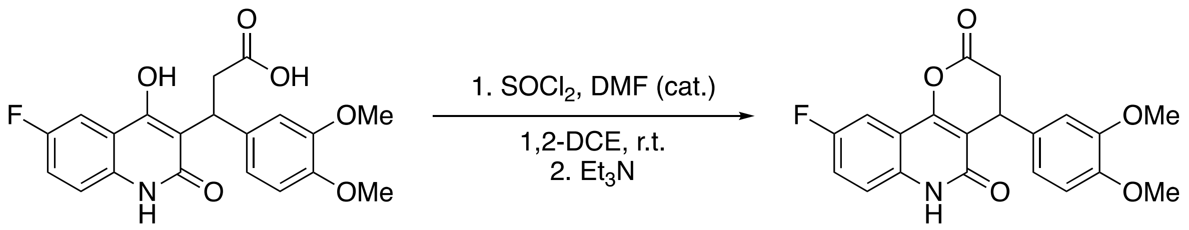

In a 100 mL round-bottomed flask equipped with a magnetic stirrer 100 mg (0.258 mmol, 1 equiv) of the starting acid **11a**, 25 mL of DCE were added. The flask was filled with argon. 103 µL (1.42 mmol, 5.5 equiv) of thionyl chloride and a drop of DMF were added. After 2 hours, 72 µL (0.516 mmol, 2 equiv) of triethylamine was added to the reaction mixture. The reaction mixture was stirred at room temperature. The extent of reaction product formation was monitored by TLC. The reaction mixture was then concentrated under reduced pressure. The residue was dissolved in ethyl acetate. The mixture was washed with water, saturated $NaHCO_3$ solution, 1 M HCl solution and saturated NaCl solution. The organic phase was dried over anhydrous sodium sulfate, concentrated and dried under reduced pressure. As a result, the product was obtained with a mass of 87 mg in 91% yield.

^{1}H NMR spectrum (400 MHz, DMSO-*d*$_6$) δ 12.08 (s, 1H, NH), 7.52 (ddt, *J* = 9.9, 5.8, 2.9 Hz, 2H, H_{Ar}), 7.42 (dd, *J* = 9.9, 4.7 Hz, 1H, H_{Ar}), 6.94 (d, *J* = 2.2 Hz, 1H, H_{Ar}), 6.82 (d, *J* = 8.4 Hz, 1H, H_{Ar}), 6.51 (dd, *J* = 8.4, 2.1 Hz, 1H, H_{Ar}), 4.46 (d, *J* = 6.9 Hz, 1H, CH), 3.73 (s, 3H, OMe), 3.68 (s, 3H, OMe), 3.43 (dd, *J* = 16.0, 7.4 Hz, 1H, CH_2), 2.97 (dd, *J* = 15.9, 1.3 Hz, 1H, CH_2).

^{13}C NMR spectrum (100 MHz, DMSO-*d*$_6$) δ 166.2 (COO), 160.7 (C(O)NH), 157.2 (d, *J* = 239.3 Hz, C_{Ar}F) 153.8 (d, *J* = 3.2 Hz, **C**(OOC)=C), 149.1 (**C_{Ar}**OCH_3), 148.1 (**C_{Ar}**OCH_3), 135.0 (C_{Ar}), 132.9 (C_{Ar}), 119.6 (d, *J* = 24.5 Hz, C_{Ar}), 117.7 (C_{Ar}), 117.6 (C_{Ar}), 113.2 (C_{Ar}), 113.1 (C(OOC)=**C**), 111.9 (C_{Ar}), 111.2 (C_{Ar}), 106.9 (d, *J* = 24.7 Hz, C_{Ar}), 55.5 (2×OCH_3), 36.3 (CH), 34.1 (CH_2).

^{19}F NMR spectrum (376 MHz, DMSO-*d*$_6$) δ −119.60 (td, *J* = 8.8, 4.8 Hz).

ESI-HRMS (*m/z*): calc. for $[C_{20}H_{15}FNO_5]^-$ 368.0940, found 368.0942 $[M-H]^-$.

ESI-LCMS (*m/z*): calc. for $[C_{20}H_{15}FNO_5]^-$ 368.1, found 368.2 $[M-H]^-$ (95.43%).

Synthesis of 9-chloro-4-(3,4-dimethoxyphenyl)-4,6-dihydro-2*H*-pyrano[3,2-*c*]quinoline-2,5(3*H*)-dione (14b)

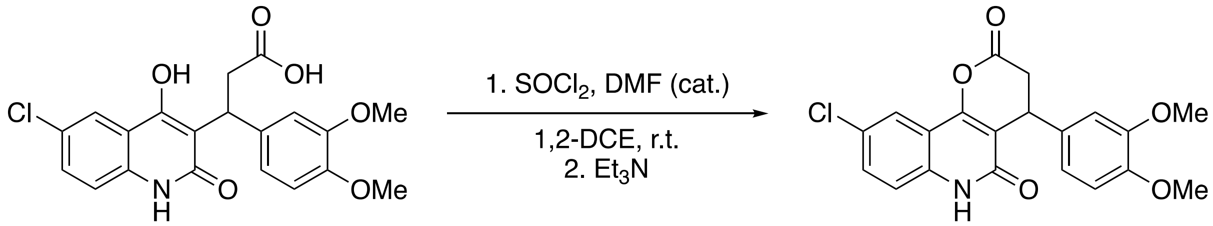

In a 100 mL round-bottomed flask equipped with a magnetic stirrer 100 mg (0.248 mmol, 1 equiv) of the starting acid **11b**, 25 mL of DCE were added. The flask was filled with argon. 100 µL (1.36 mmol, 5.5 equiv) of thionyl chloride and a drop of DMF were added. After 2 hours, 70 µL (0.5 mmol, 2 equiv) of triethylamine was added to the reaction mixture. The reaction mixture was stirred at room temperature. The extent of reaction product formation was monitored by TLC. The reaction mixture was then concentrated under reduced pressure. The residue was dissolved in ethyl acetate. The mixture was washed with water, saturated $NaHCO_3$ solution, 1 M HCl solution and saturated NaCl solution. The organic phase was dried over anhydrous sodium sulfate, concentrated and dried under reduced pressure. As a result, the product was obtained with a mass of 72 mg in 75% yield.

^{1}H NMR spectrum (400 MHz, DMSO-d_6) δ 12.12 (s, 1H, NH), 7.74 (d, J = 2.4 Hz, 1H, H_{Ar}), 7.63 (dd, J = 8.8, 2.4 Hz, 1H, H_{Ar}), 7.39 (d, J = 8.8 Hz, 1H, H_{Ar}), 6.93 (d, J = 2.2 Hz, 1H, H_{Ar}), 6.81 (d, J = 8.3 Hz, 1H, H_{Ar}), 6.52 (dd, J = 8.3, 2.1 Hz, 1H, H_{Ar}), 4.46 (d, J = 7.0 Hz, 1H, CH), 3.73 (s, 3H, OCH_3), 3.68 (s, 3H, OCH_3), 3.43 (dd, J = 16.0, 7.5 Hz, 1H, CH_2), 2.98 (dd, J = 16.0, 1.6 Hz, 1H, CH_2).

^{13}C NMR spectrum (100 MHz, DMSO-d_6) δ 166.1 (COO), 160.8 (C(O)NH), 153.5 (**C**(OOC)=C), 149.1 (**C$_{Ar}$**OCH_3), 148.1 (**C$_{Ar}$**OCH_3), 136.9 (C_{Ar}), 132.8 (C_{Ar}), 131.3 (C_{Ar}), 126.3 (C_{Ar}), 120.7 (C_{Ar}), 117.7 (C_{Ar}), 117.5 (C_{Ar}), 113.6 (C_{Ar}), 113.1 (C(OOC)=**C**), 111.9 (C_{Ar}), 111.2 (C_{Ar}), 55.5 (OCH_3), 36.3 (CH), 34.2 (CH_2).

ESI-HRMS (*m/z*): calc. for $[C_{20}H_{15}ClNO_5]^-$ 384.0644, found 384.0648 $[M-H]^-$.

ESI-LCMS (*m/z*): calc. for $[C_{20}H_{15}ClNO_5]^-$ 384.06, found 384.15 $[M-H]^-$ (85.248%).

Synthesis of 9-bromo-4-(3,4-dimethoxyphenyl)-4,6-dihydro-2*H*-pyrano[3,2-*c*]quinoline-2,5(3*H*)-dione (14c)

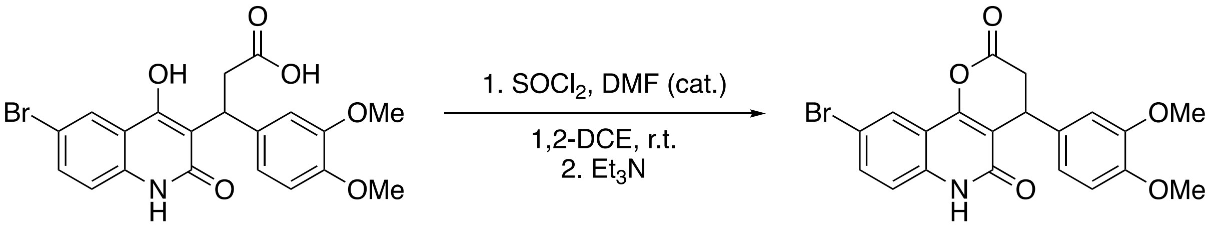

In a 100 mL round-bottomed flask equipped with a magnetic stirrer 112 mg (0.25 mmol, 1 equiv) of the starting acid **11c**, 25 mL of DCE were added. The flask was filled with argon. 100 µL (1.36 mmol, 5.5 equiv) of thionyl chloride and a drop of DMF were added. After 2 hours, 70 µL (0.5 mmol, 2 equiv) of triethylamine was added to the reaction mixture. The reaction mixture was stirred at room temperature. The extent of reaction product formation was monitored by TLC. The reaction mixture was then concentrated under reduced pressure. The residue was dissolved in ethyl acetate. The mixture was washed with water, saturated $NaHCO_3$ solution, 1 M HCl solution and saturated NaCl solution. The organic phase was dried over anhydrous sodium sulfate, concentrated and dried under reduced pressure. As a result, the product was obtained in a mass of 110 mg in quantitative yield.

^{1}H NMR spectrum (400 MHz, DMSO-d_6) δ 12.13 (s, 1H, NH), 7.89 (d, *J* = 2.2 Hz, 1H, H_{Ar}), 7.78 (dd, *J* = 8.8, 2.2 Hz, 1H, H_{Ar}), 7.34 (d, *J* = 8.8 Hz, 1H, H_{Ar}), 6.92 (d, *J* = 2.0 Hz, 1H, H_{Ar}), 6.81 (d, *J* = 8.4 Hz, 1H, H_{Ar}), 6.51 (dd, *J* = 8.3, 1.9 Hz, 1H, H_{Ar}), 4.45 (d, *J* = 7.1 Hz, 1H, CH), 3.72 (s, 3H, OCH_3), 3.69 (s, 3H, OCH_3), 3.42 (dd, *J* = 16.0, 7.5 Hz, 1H, CH_2), 2.96 (d, *J* = 15.8 Hz, 1H, CH_2).

^{13}C NMR spectrum (100 MHz, DMSO-d_6) δ 166.1 (CH_2**C**OO), 160.8 (C(O)NH), 153.4 (CH_2CO_2**C**), 149.1 (**C_{Ar}**OCH_3), 148.1 (**C_{Ar}**OCH_3), 137.2 (C_{Ar}), 133.9 (C_{Ar}), 132.8 (C_{Ar}), 123.7 (C_{Ar}), 117.73 (C_{Ar}), 117.66 (C_{Ar}), 114.1 (C_{Ar}), 114.0 (C_{Ar}), 113.0 (**C**=CO(O)C), 111.9 (C_{Ar}), 111.2 (C_{Ar}), 55.5 (2×OMe), 36.3 (CH), 34.2 (CH_2).

ESI-HRMS (*m/z*): calc. for $[C_{20}H_{15}{}^{79}BrNO_5]^-$ 428.0139, found 428.0142 $[M{-}H]^-$.

ESI-LCMS (*m/z*): calc. for $[C_{20}H_{17}{}^{79}BrNO_5]^+$ 430.0, found 430.1 $[M+H]^+$ (90.253%).

NMR spectra of synthesized novel compounds

^{1}H NMR spectrum of compound 10a (400 MHz, DMSO-d_6)

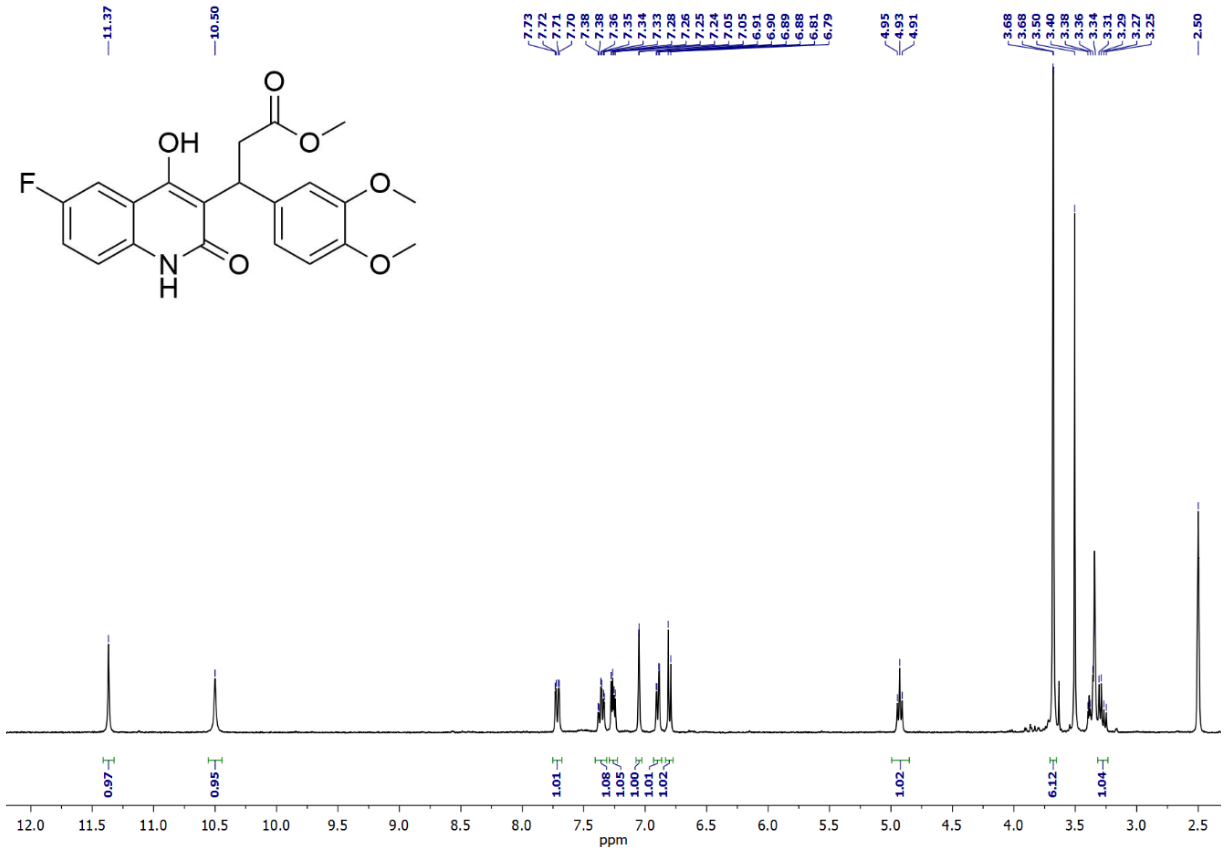

^{13}C NMR spectrum of compound 10a (100 MHz, DMSO-d_6)

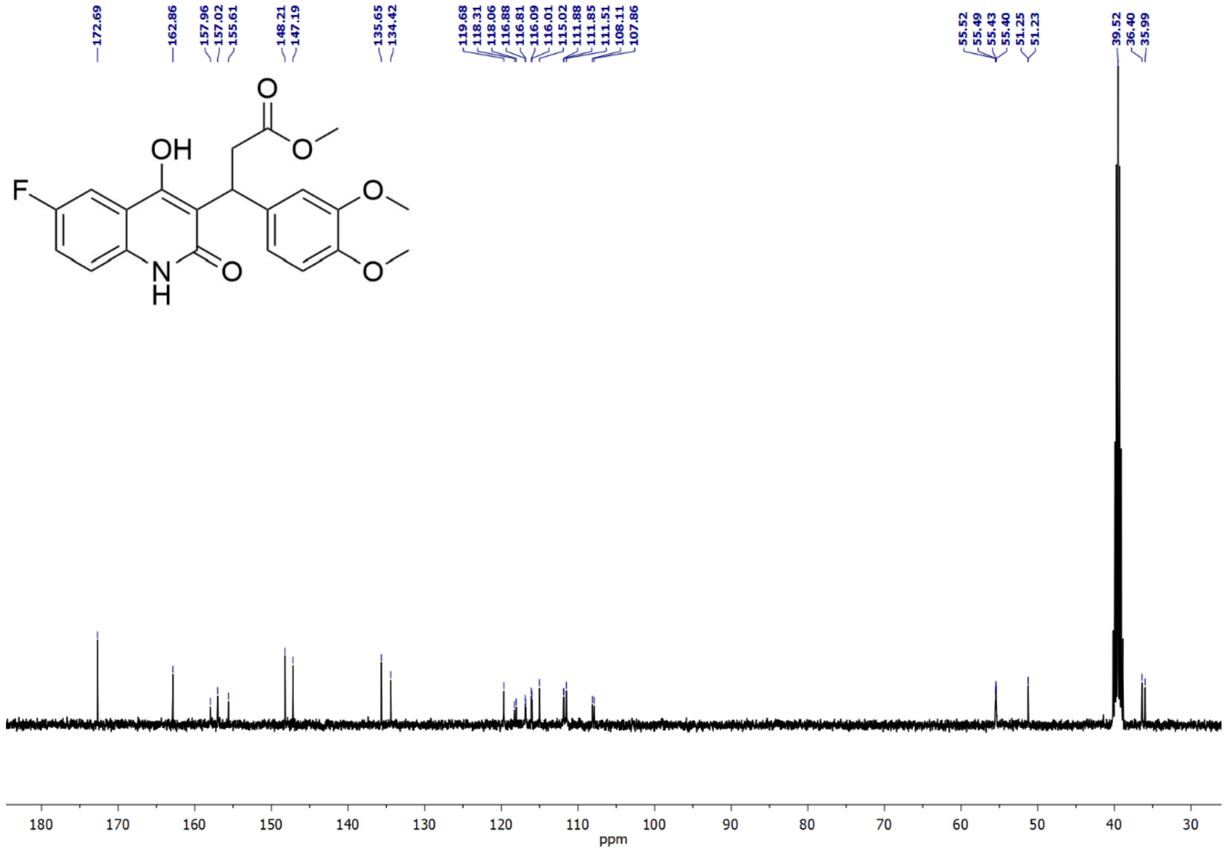

^{1}H NMR spectrum of compound 10b (400 MHz, DMSO-d_6)

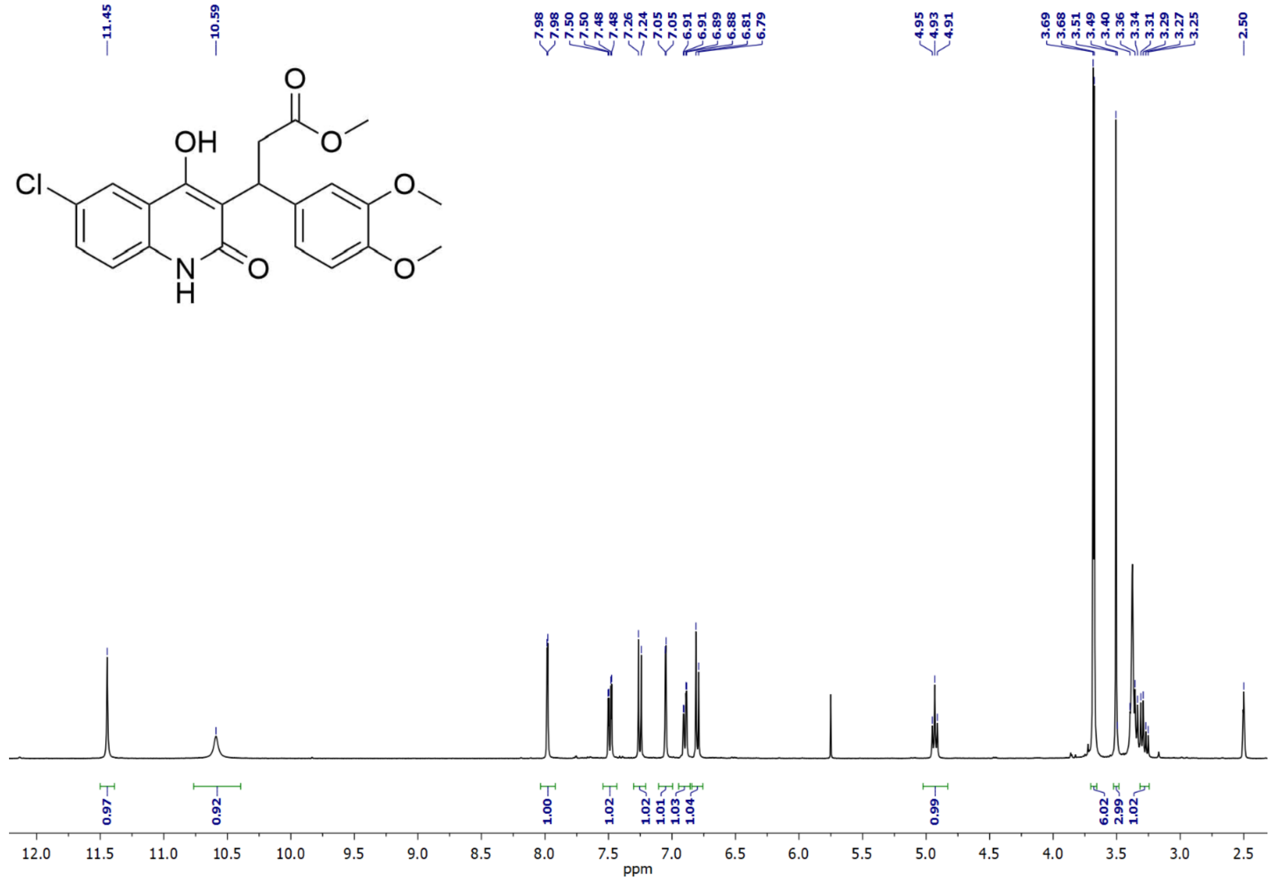

^{13}C NMR spectrum of compound 10b (100 MHz, DMSO-d_6)

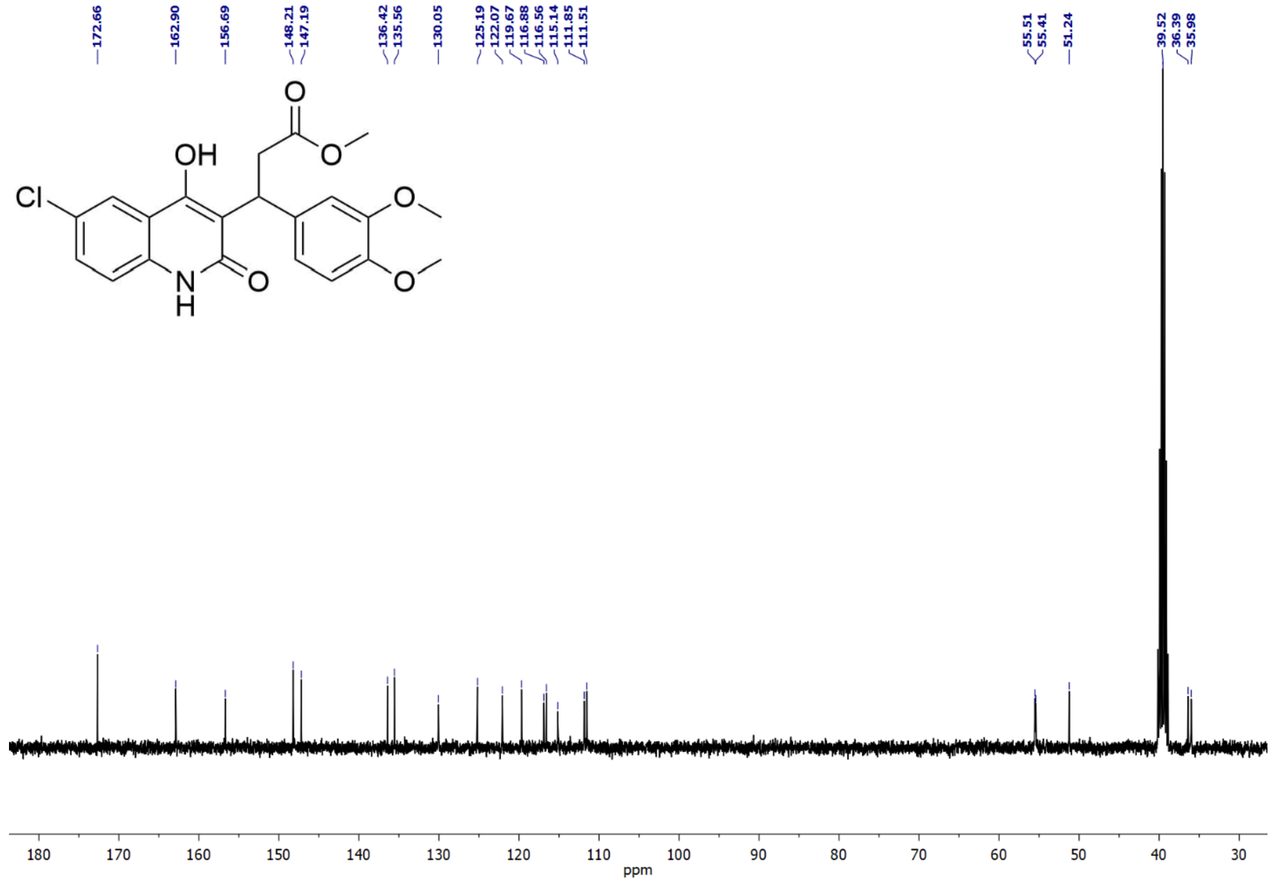

^{1}H NMR spectrum of compound 10c (400 MHz, DMSO-d_6)

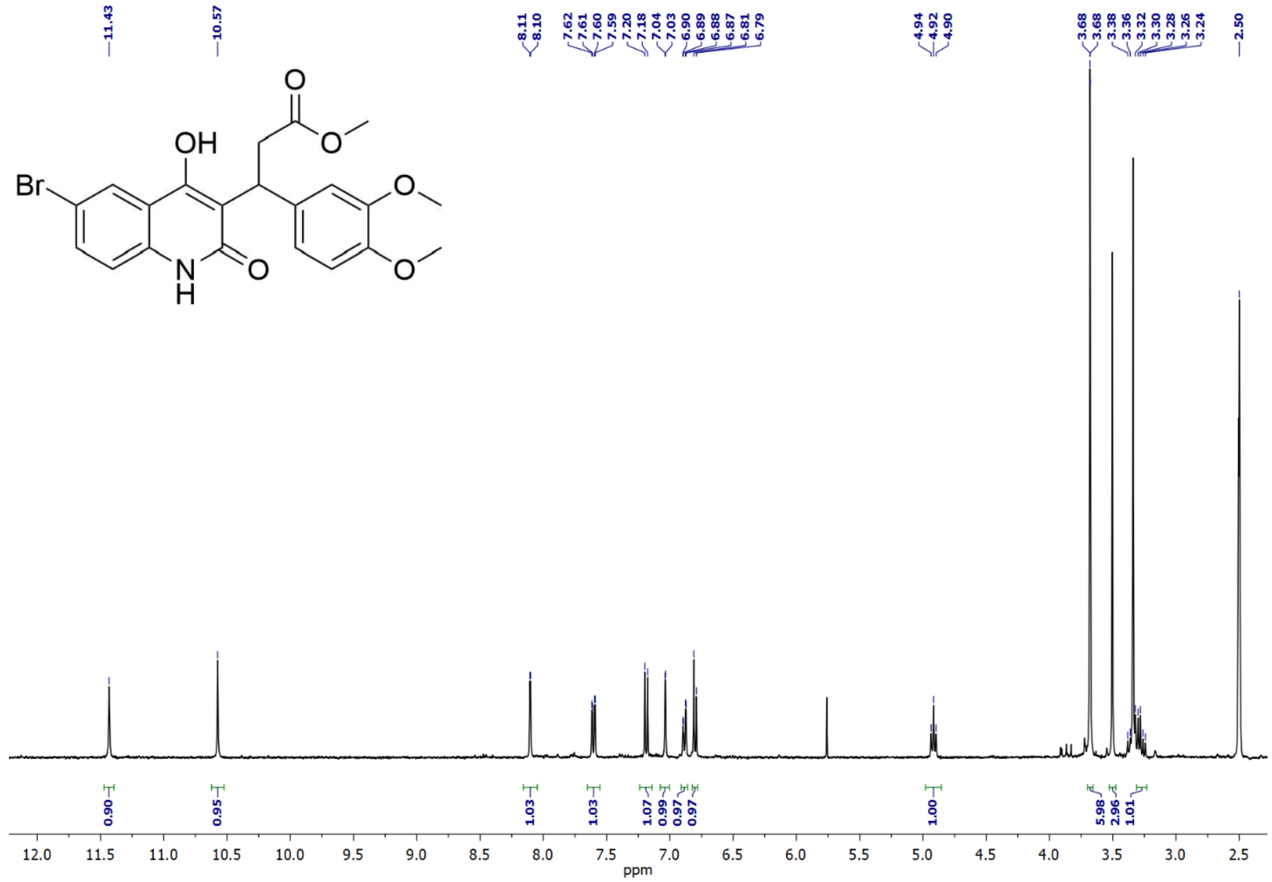

^{13}C NMR spectrum of compound 10c (100 MHz, DMSO-d_6)

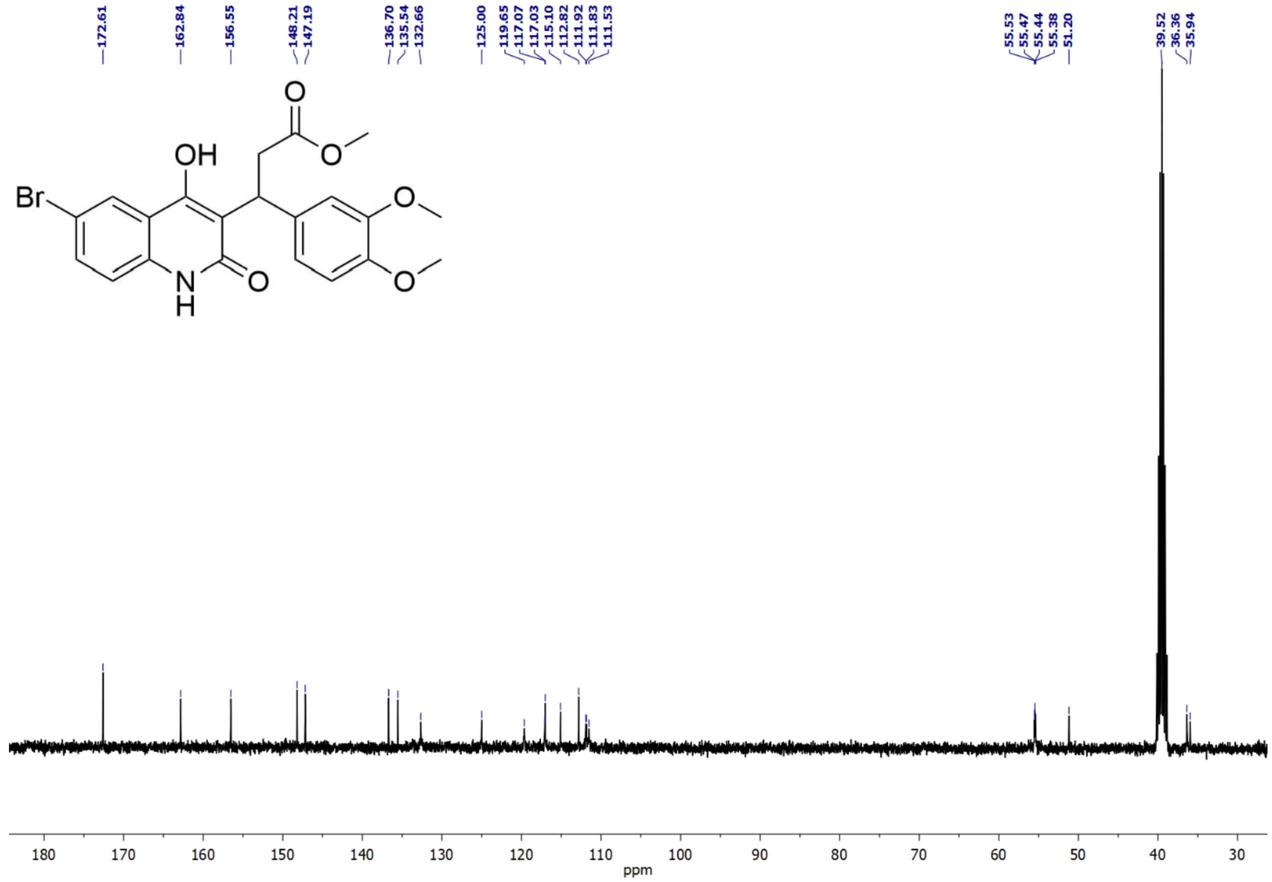

^{1}H NMR spectrum of compound 9a (400 MHz, DMSO-d_6)

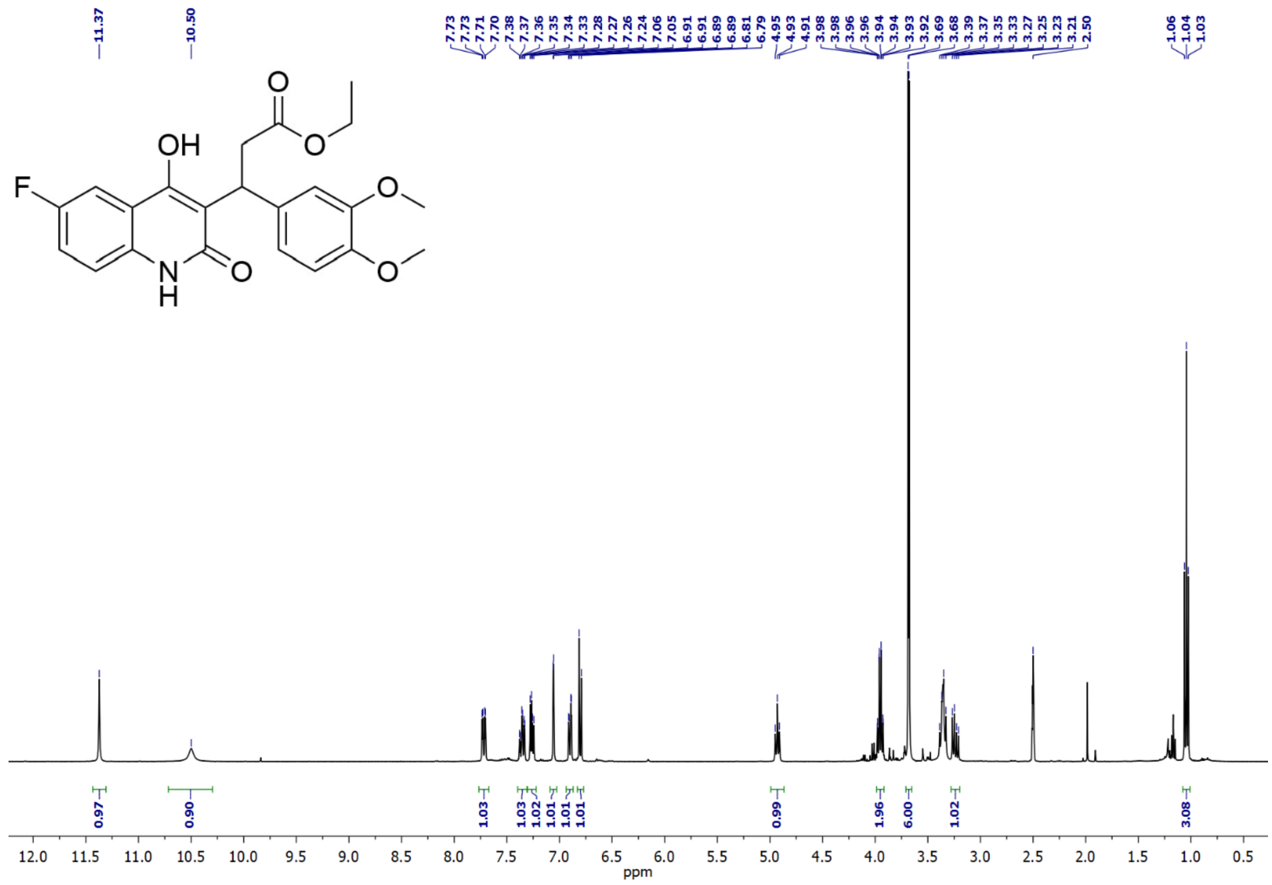

^{13}C NMR spectrum of compound 9a (100 MHz, DMSO-d_6)

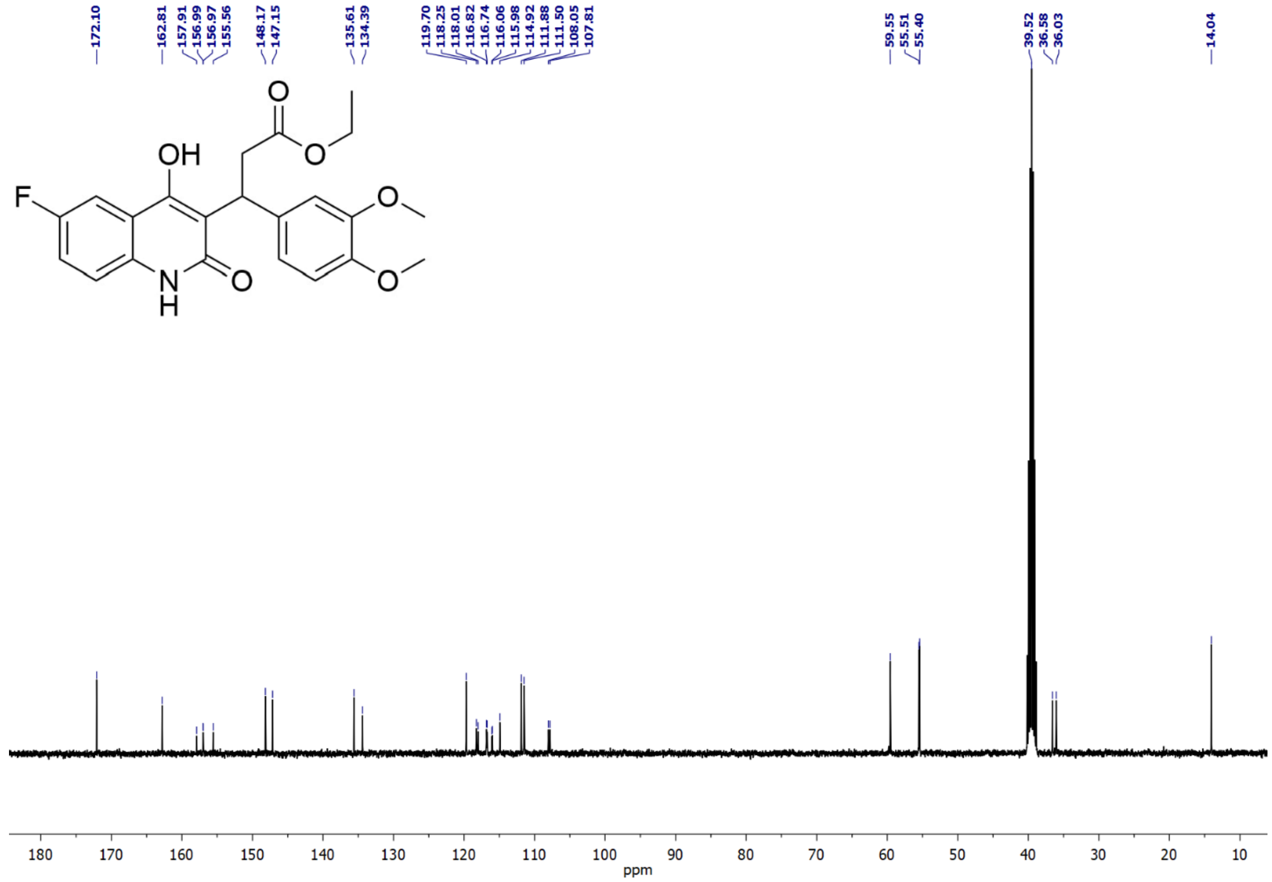

^{1}H NMR spectrum of compound 9b (400 MHz, DMSO-d_6)

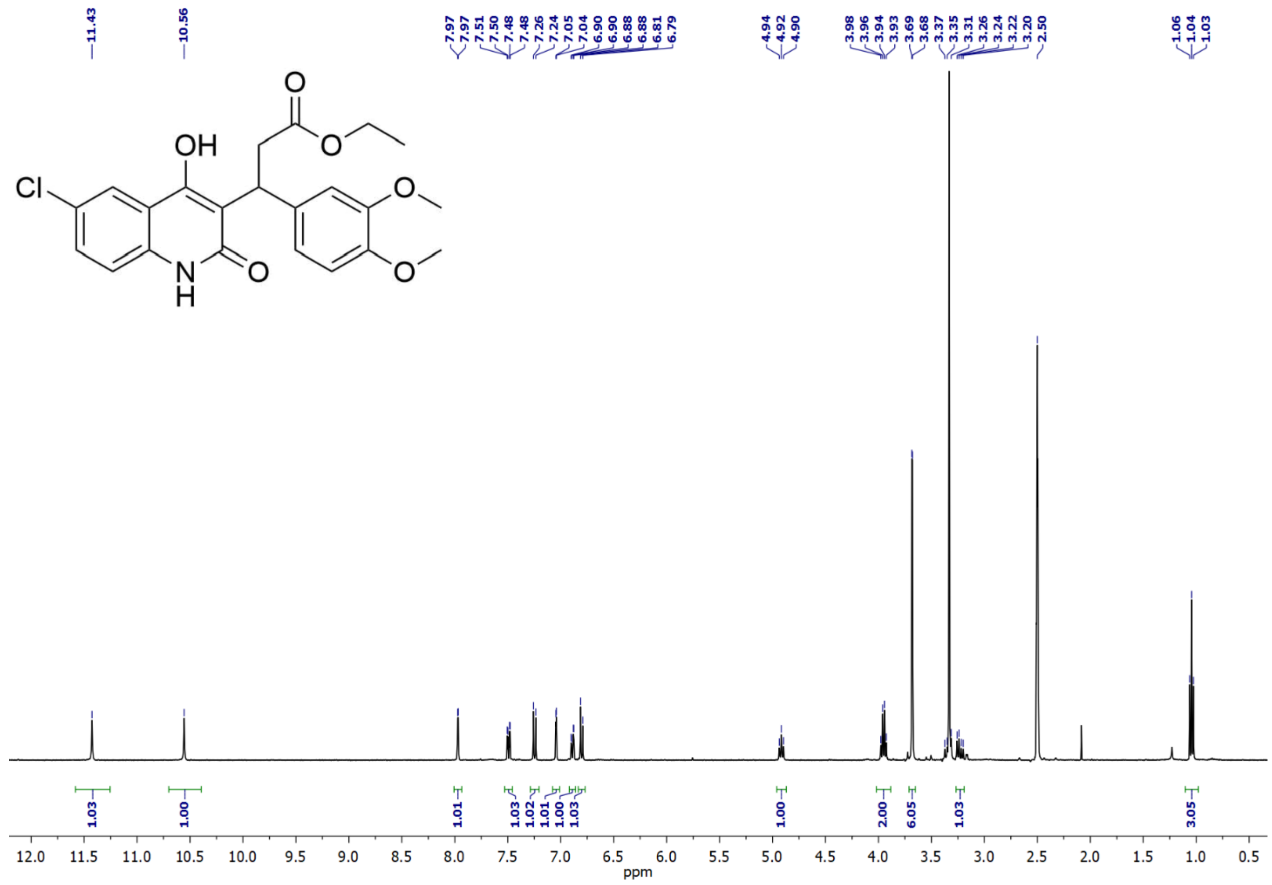

^{13}C NMR spectrum of compound 9b (100 MHz, DMSO-d_6)

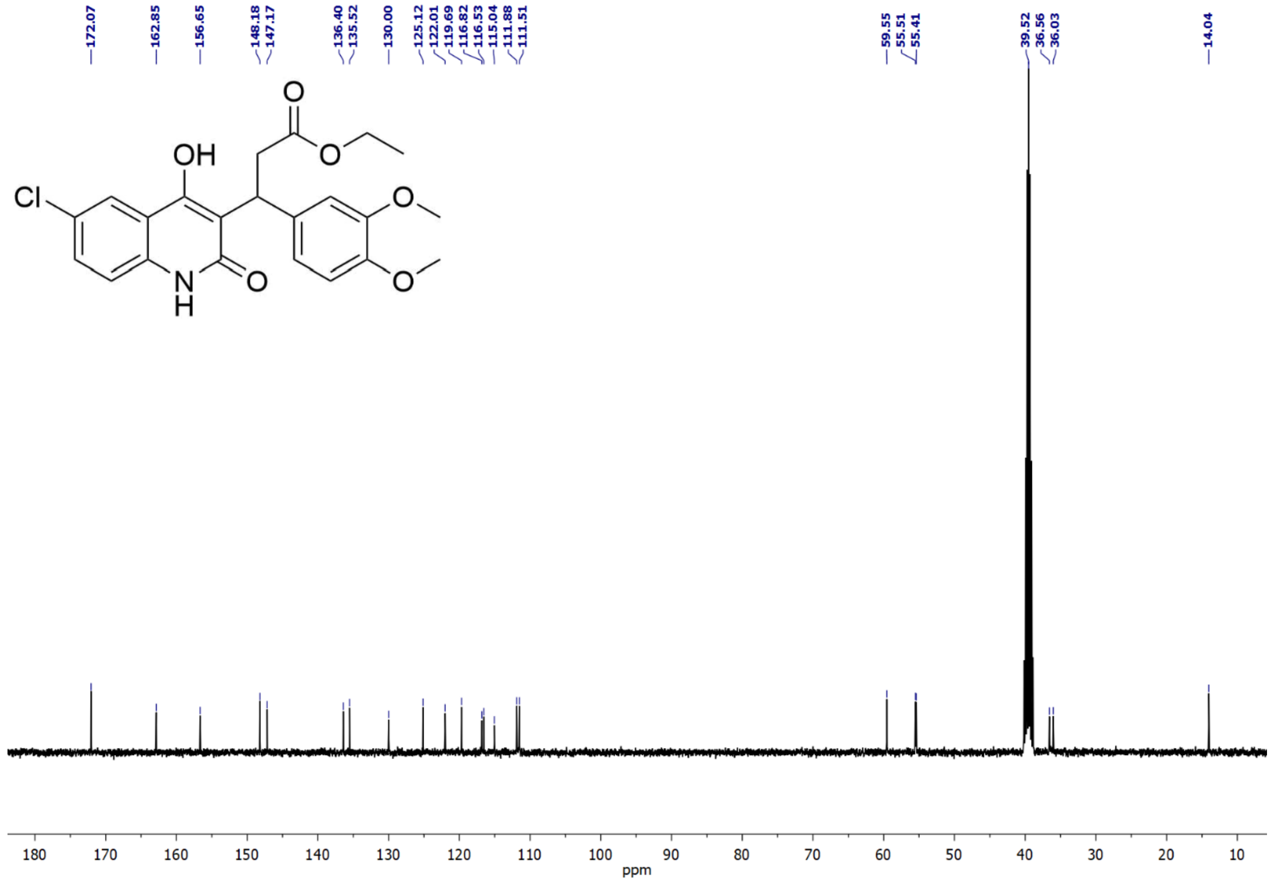

^{1}H NMR spectrum of compound 9c (400 MHz, DMSO-d_6)

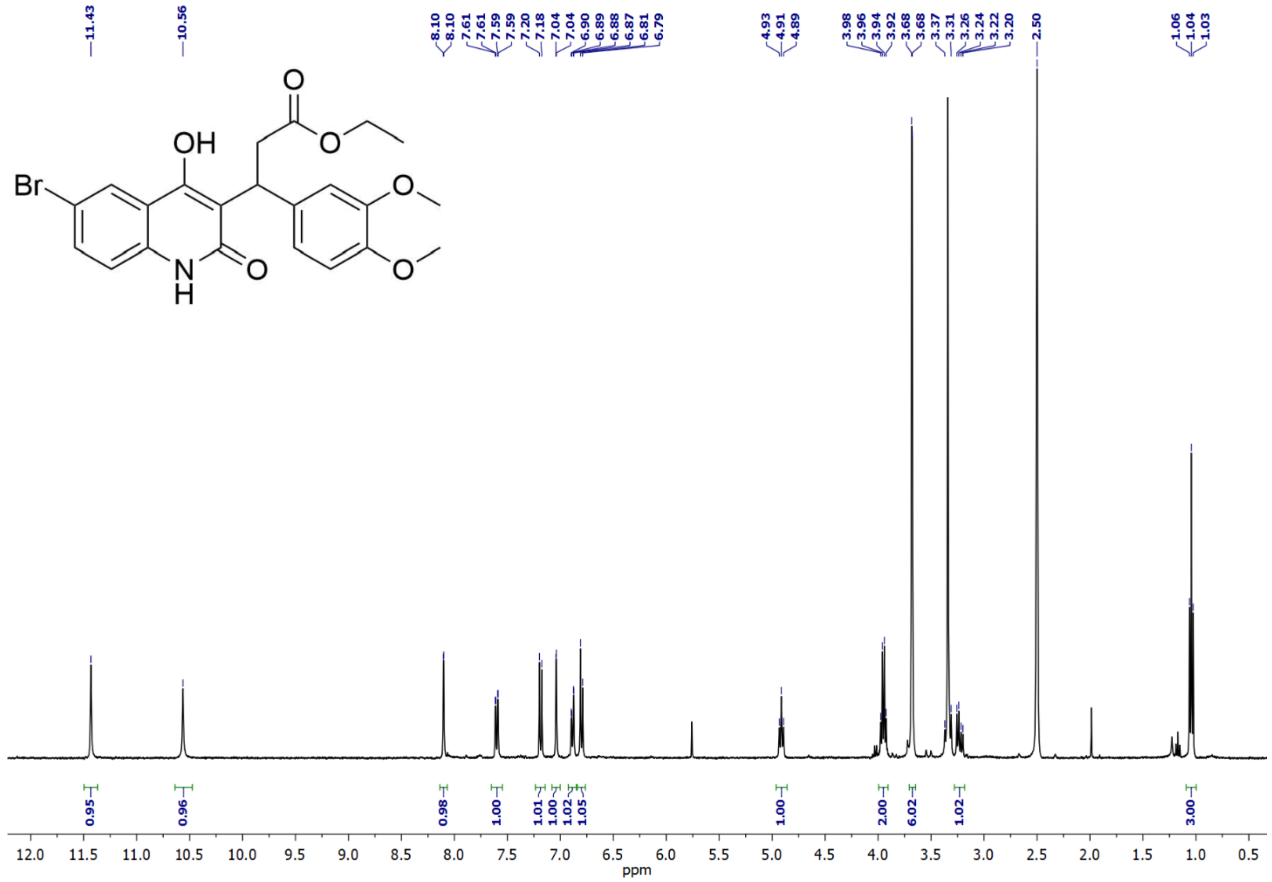

^{13}C NMR spectrum of compound 9c (100 MHz, DMSO-d_6)

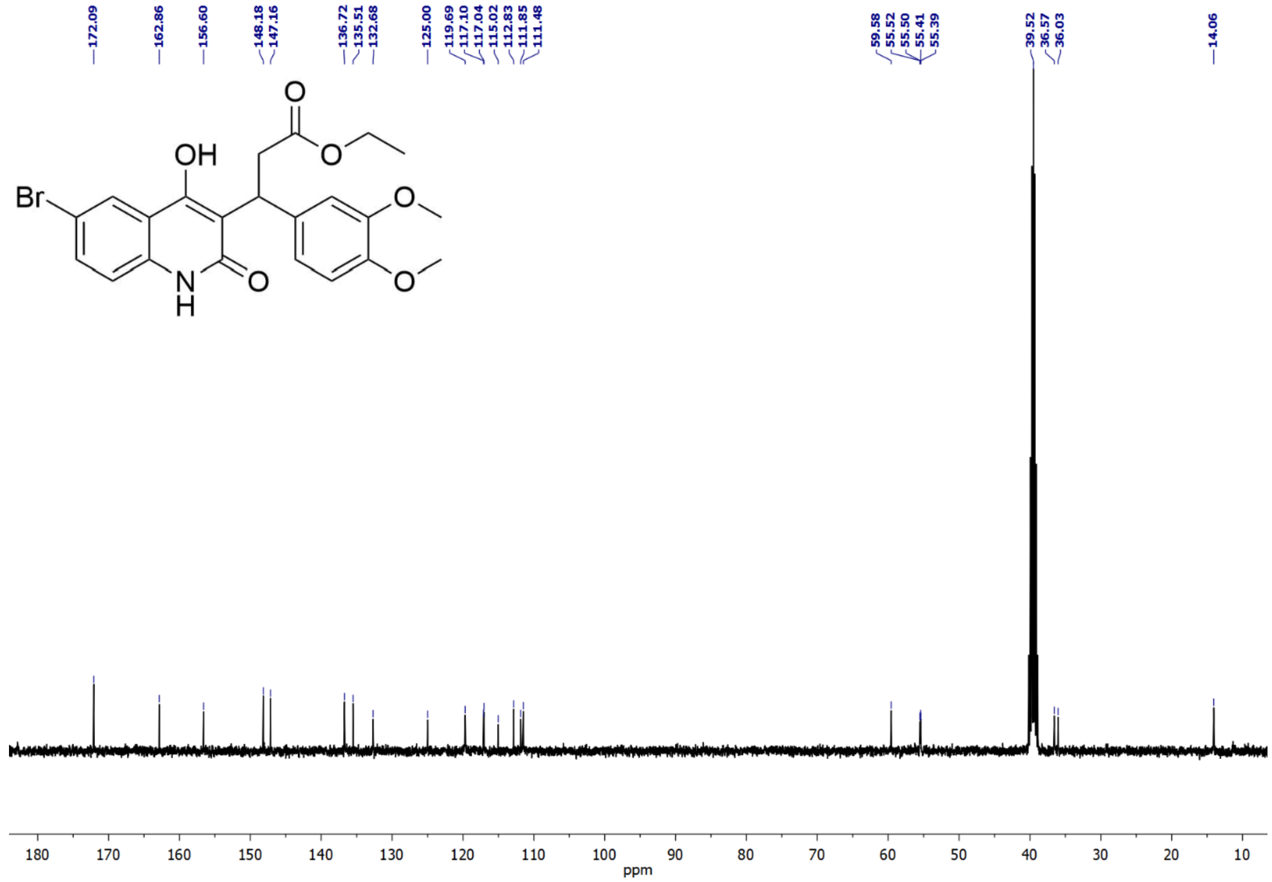

^{1}H NMR spectrum of compound 11a (400 MHz, DMSO-d_6)

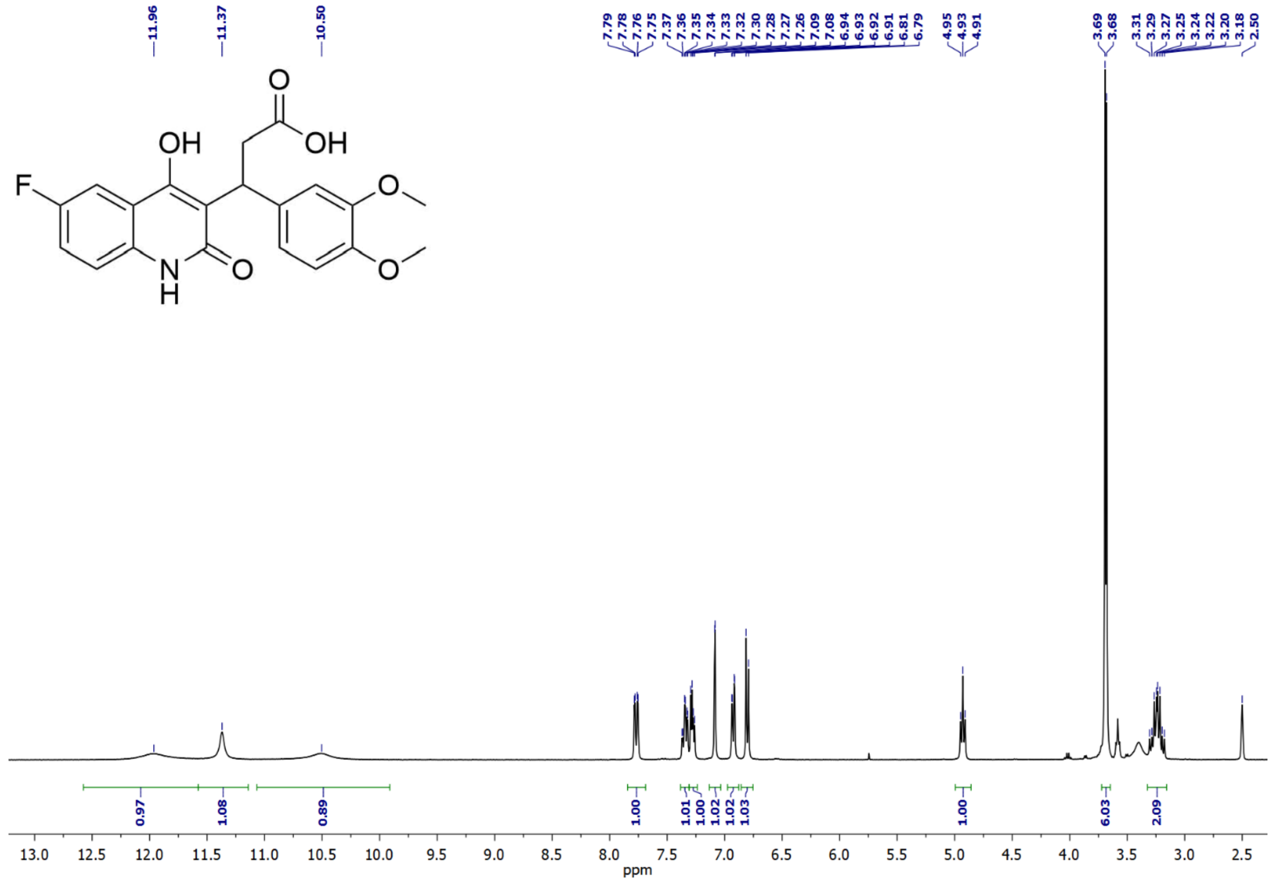

^{13}C NMR spectrum of compound 11a (100 MHz, DMSO-d_6)

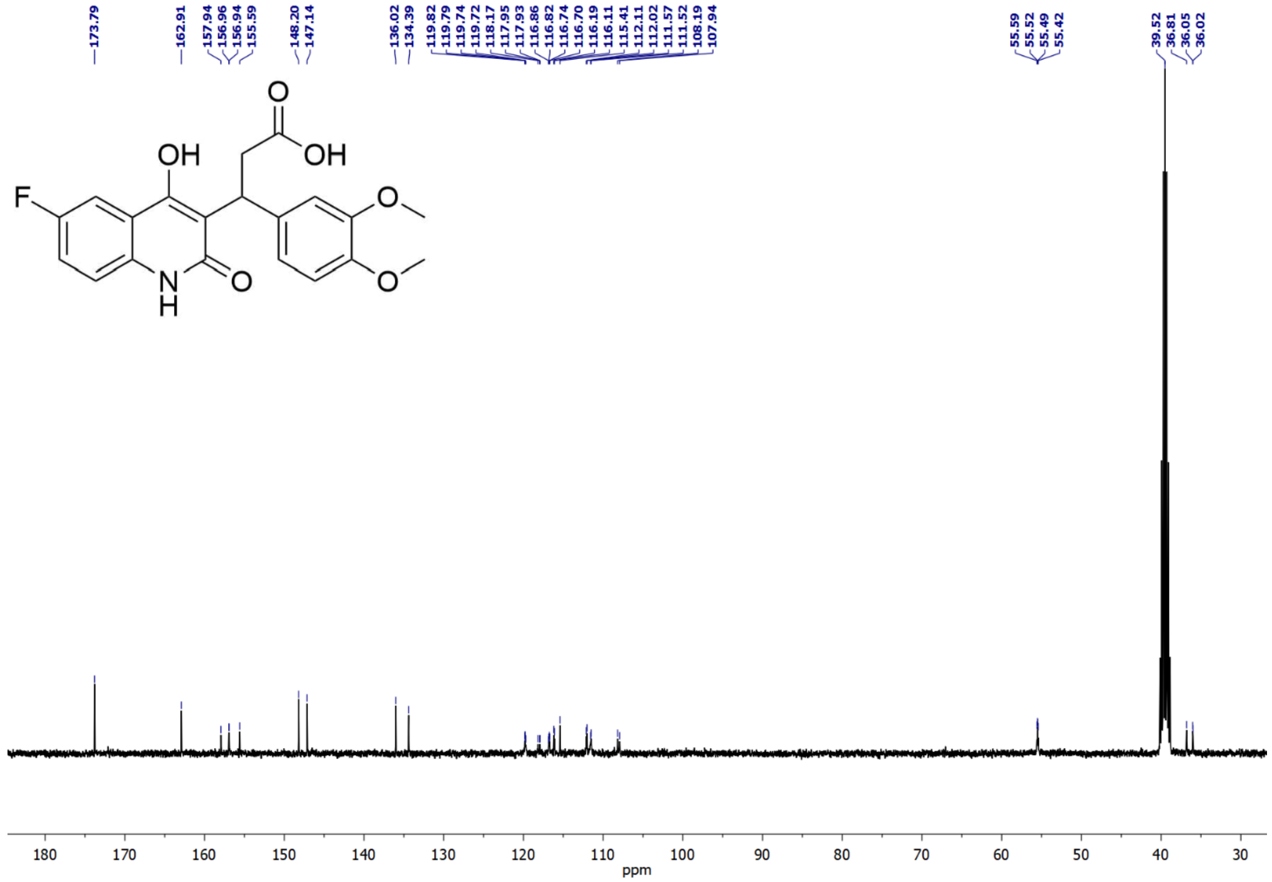

^{1}H NMR spectrum of compound 11b (400 MHz, DMSO-d_6)

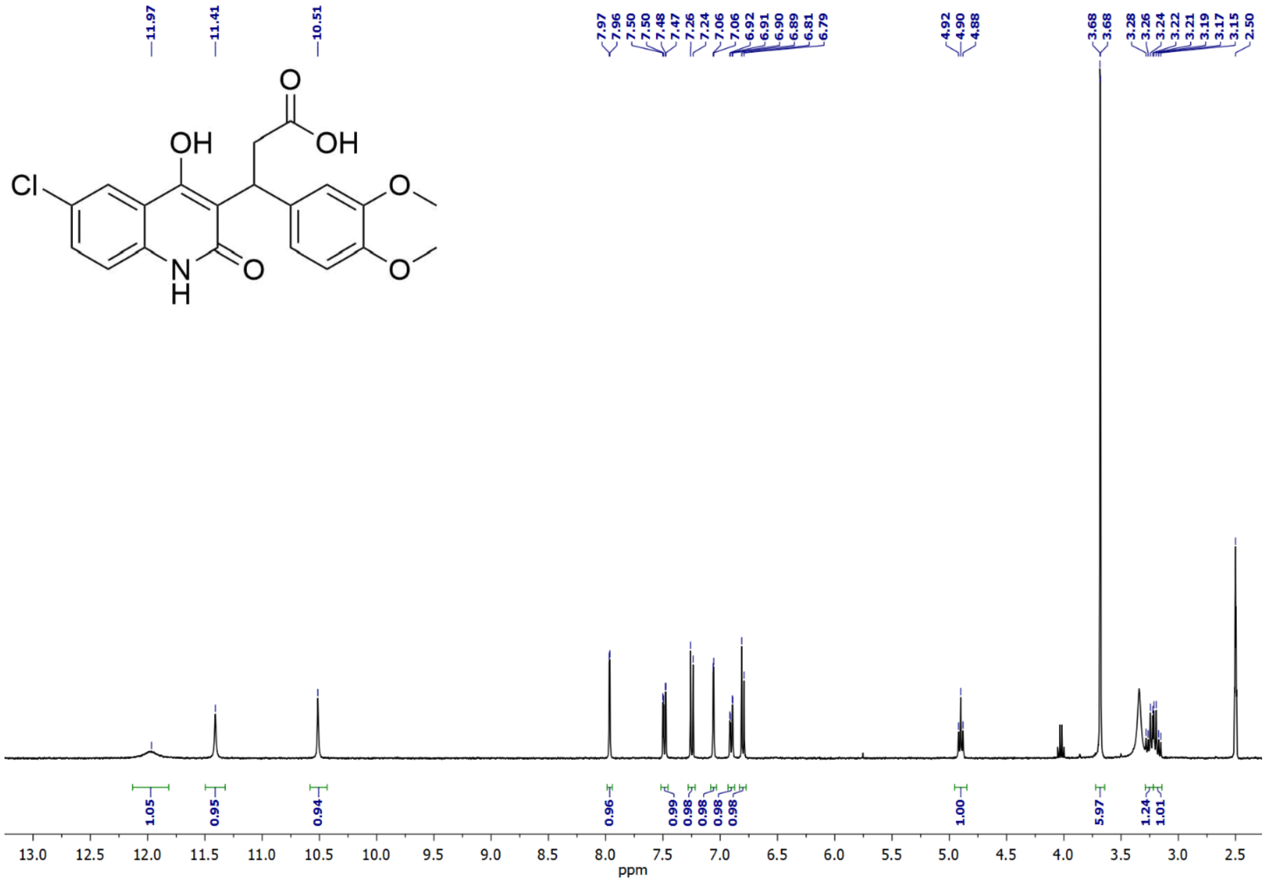

^{13}C NMR spectrum of compound 11b (100 MHz, DMSO-d_6)

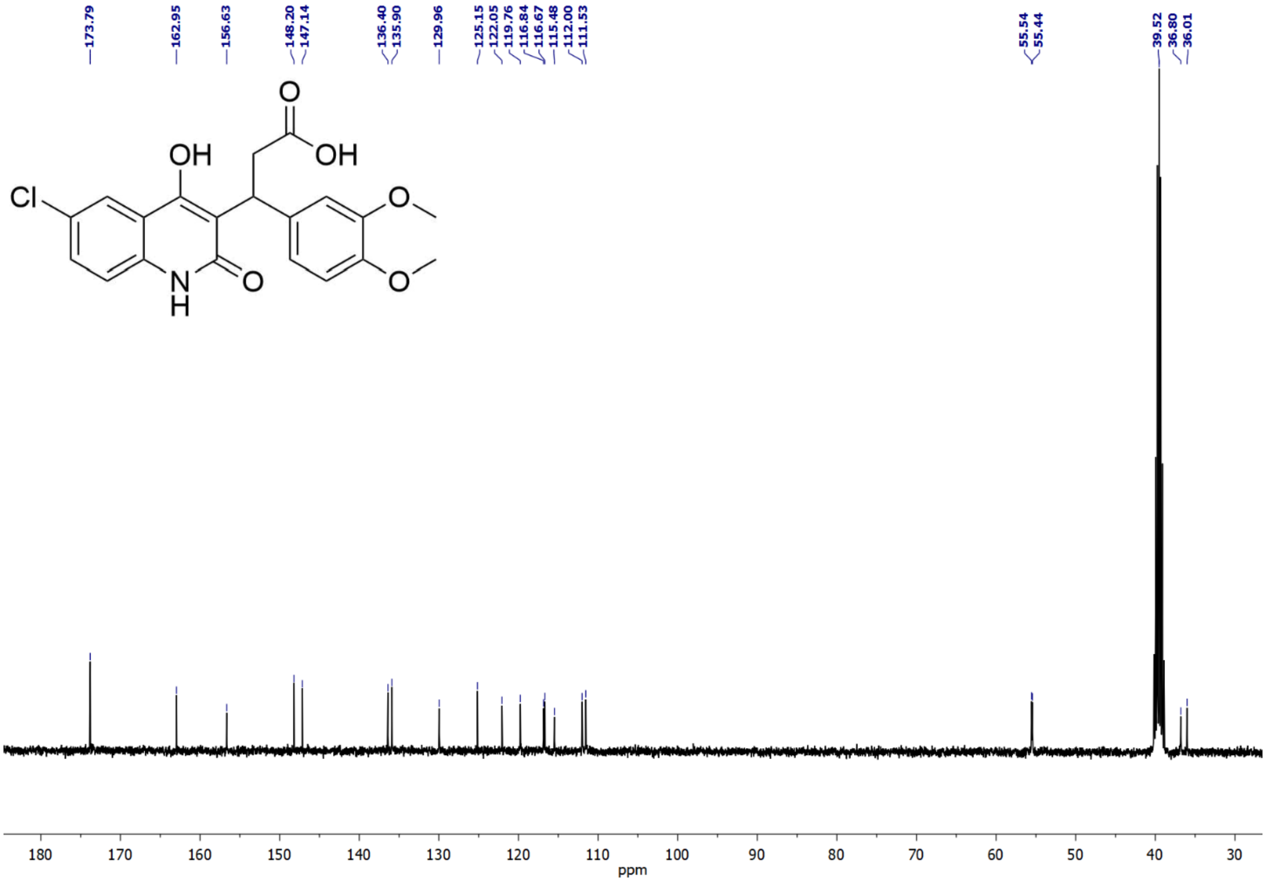

^{1}H NMR spectrum of compound 11c (400 MHz, DMSO-d_6)

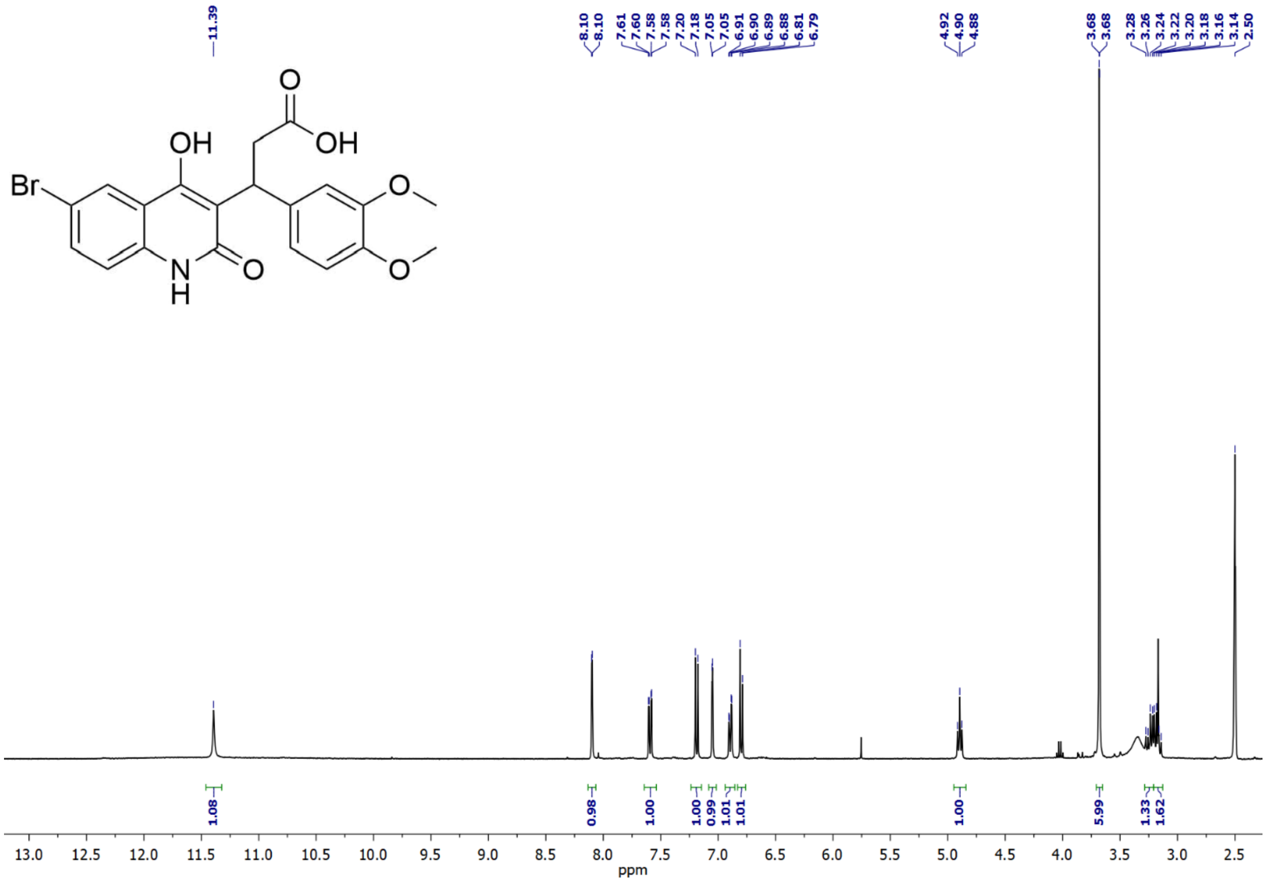

^{13}C NMR spectrum of compound 11c (100 MHz, DMSO-d_6)

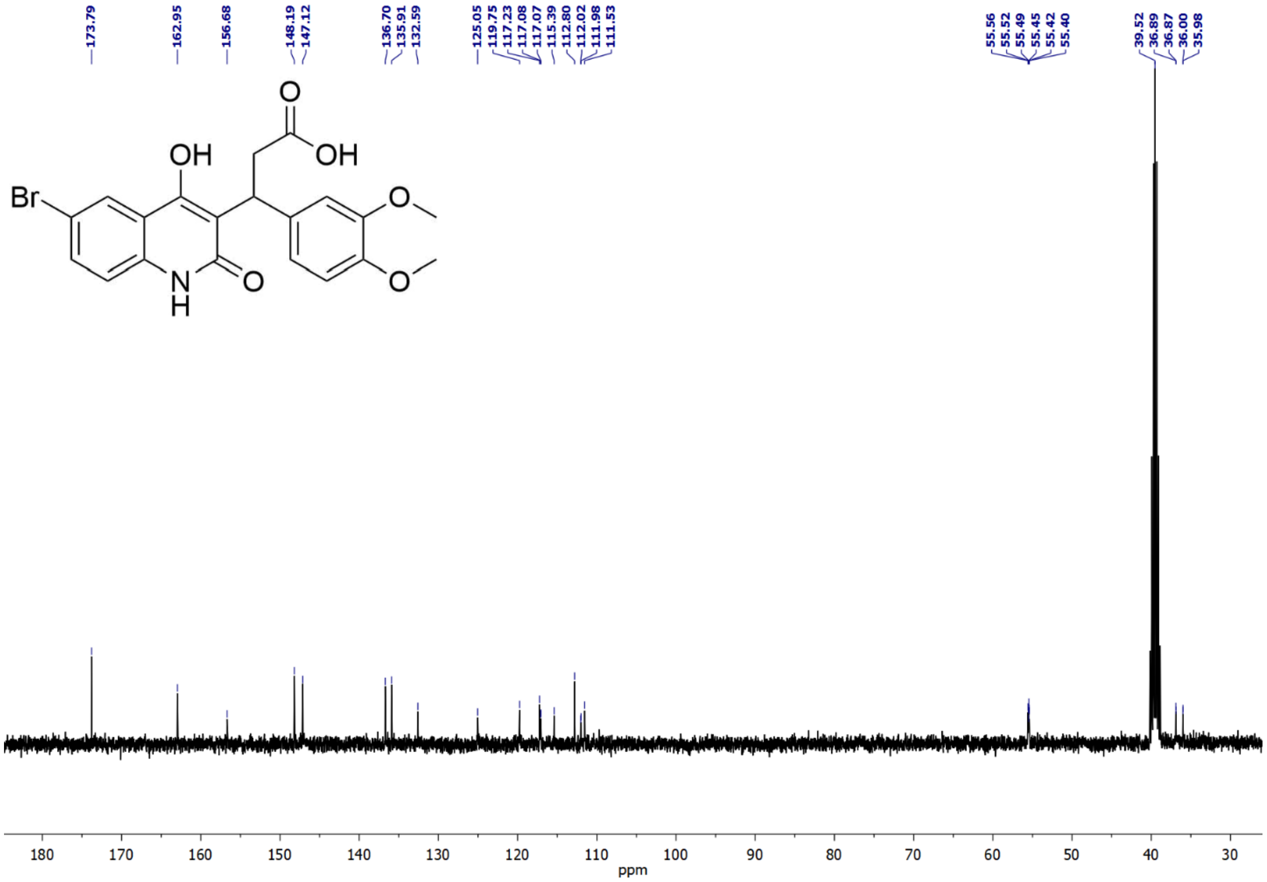

^{1}H NMR spectrum of compound 12a (400 MHz, DMSO-d_6)

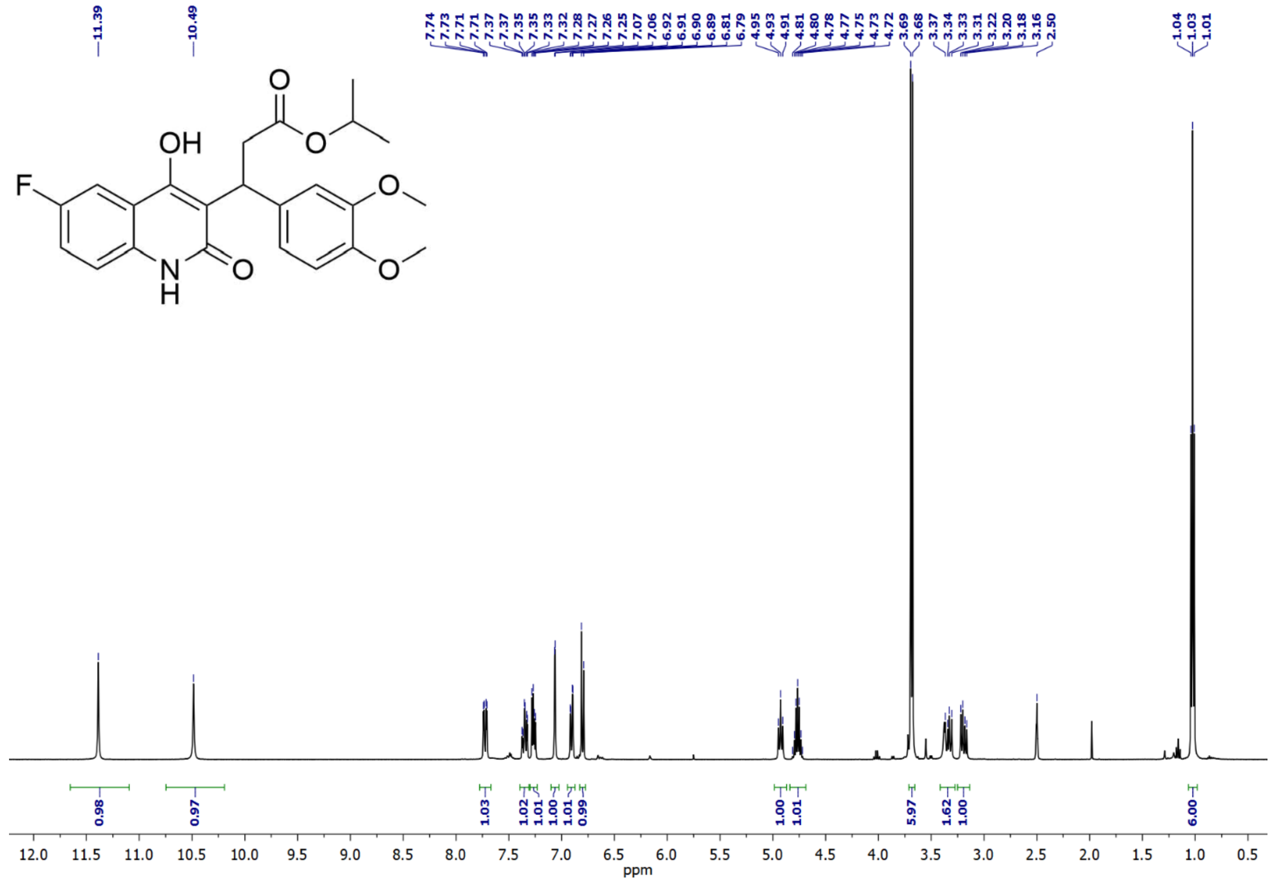

^{13}C NMR spectrum of compound 12a (100 MHz, DMSO-d_6)

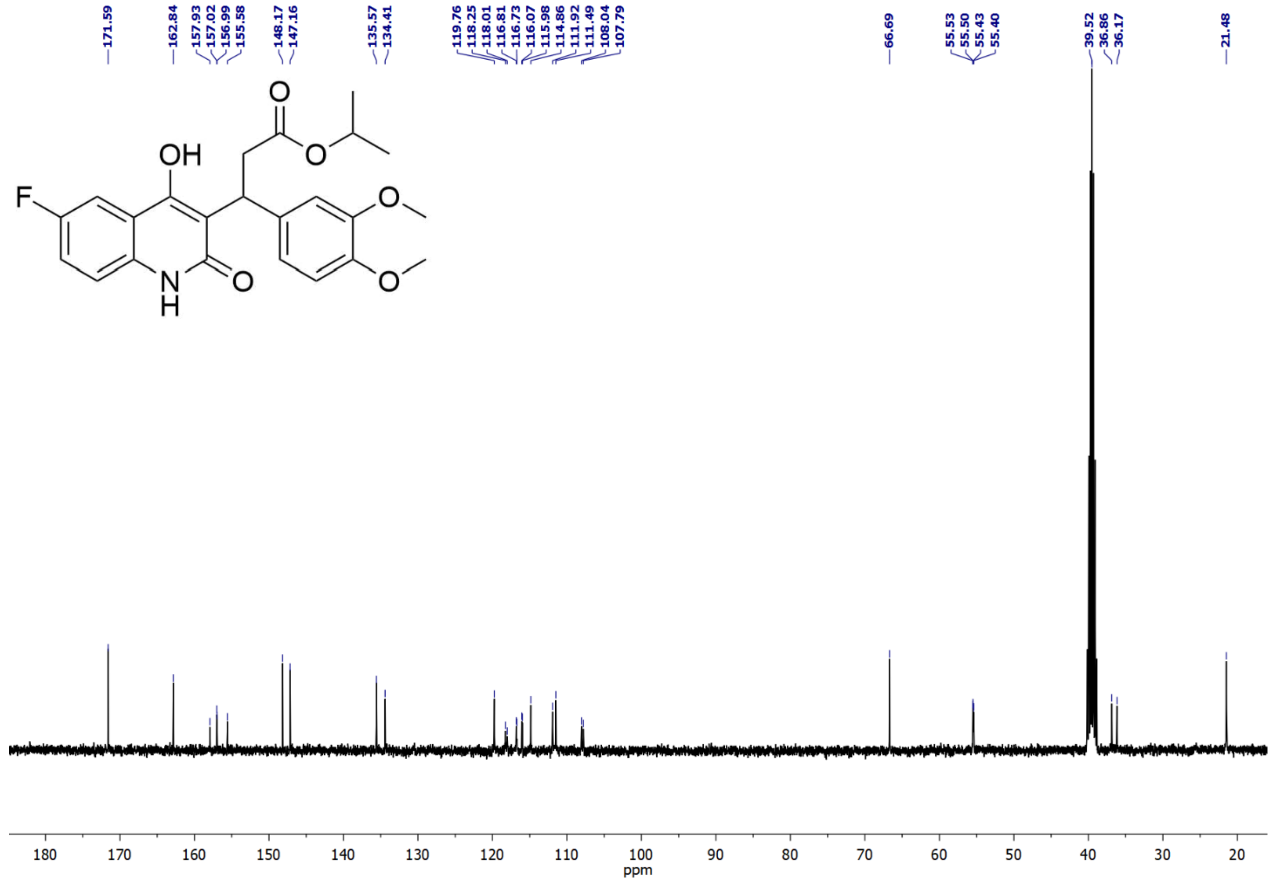

^{1}H NMR spectrum of compound 12b (400 MHz, DMSO-d_6)

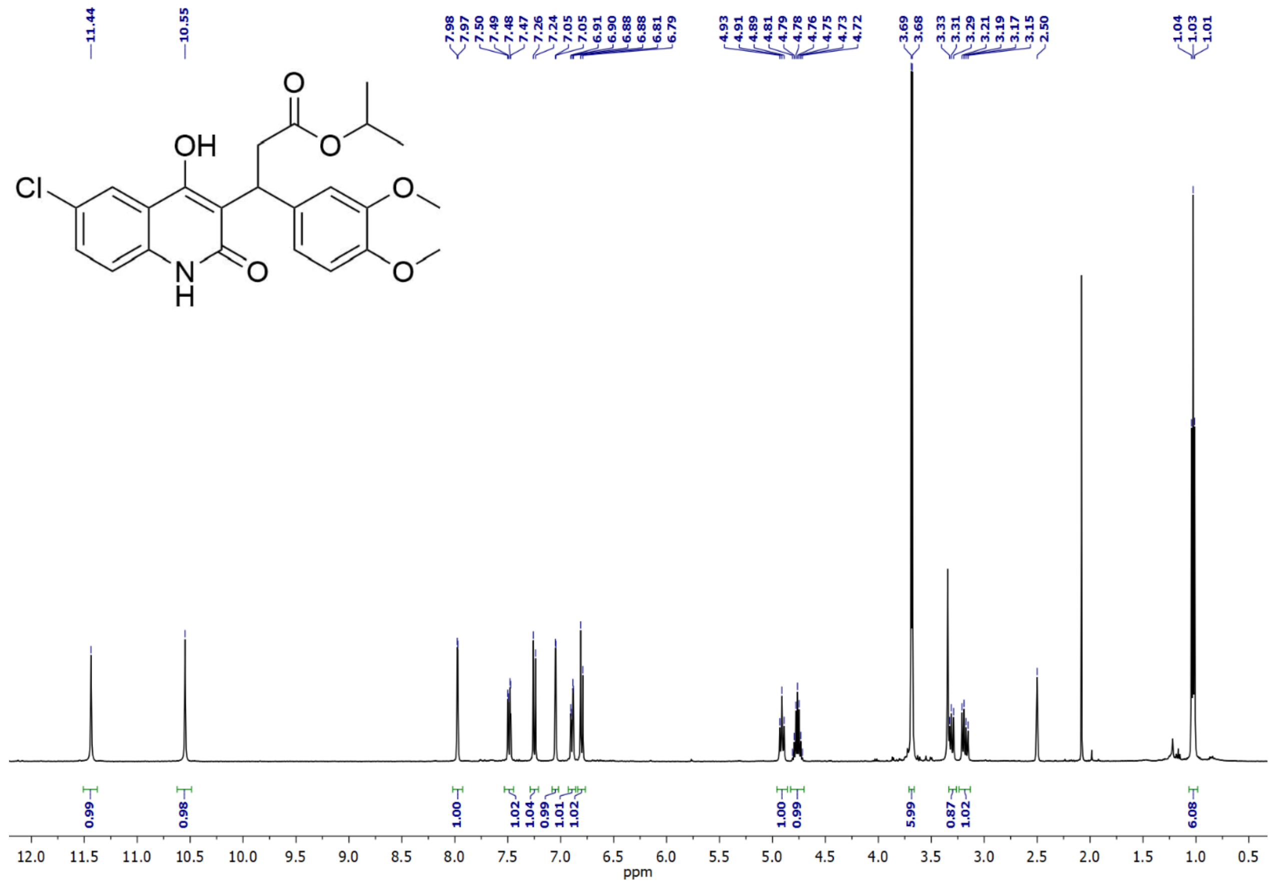

^{13}C NMR spectrum of compound 12b (100 MHz, DMSO-d_6)

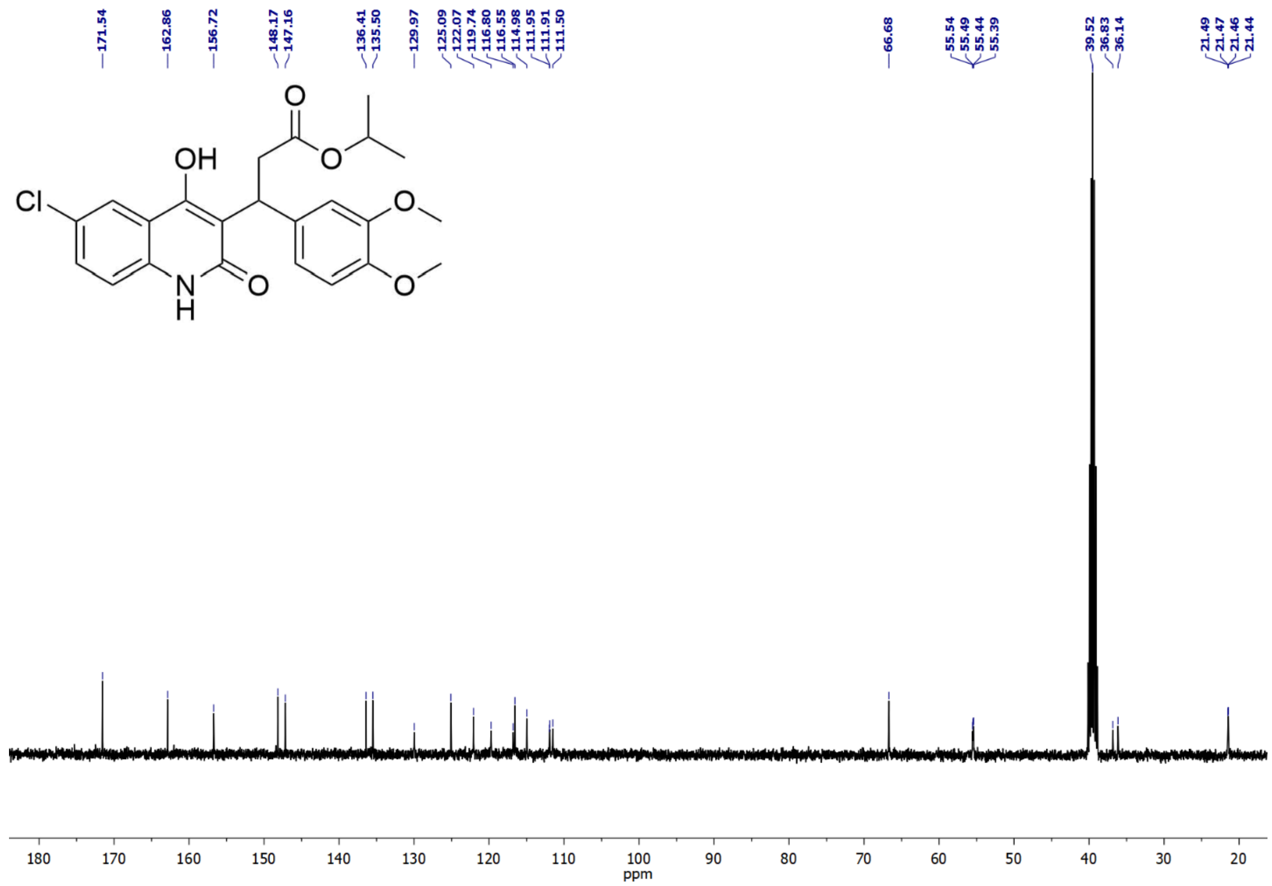

^{1}H NMR spectrum of compound 12c (400 MHz, DMSO-d_6)

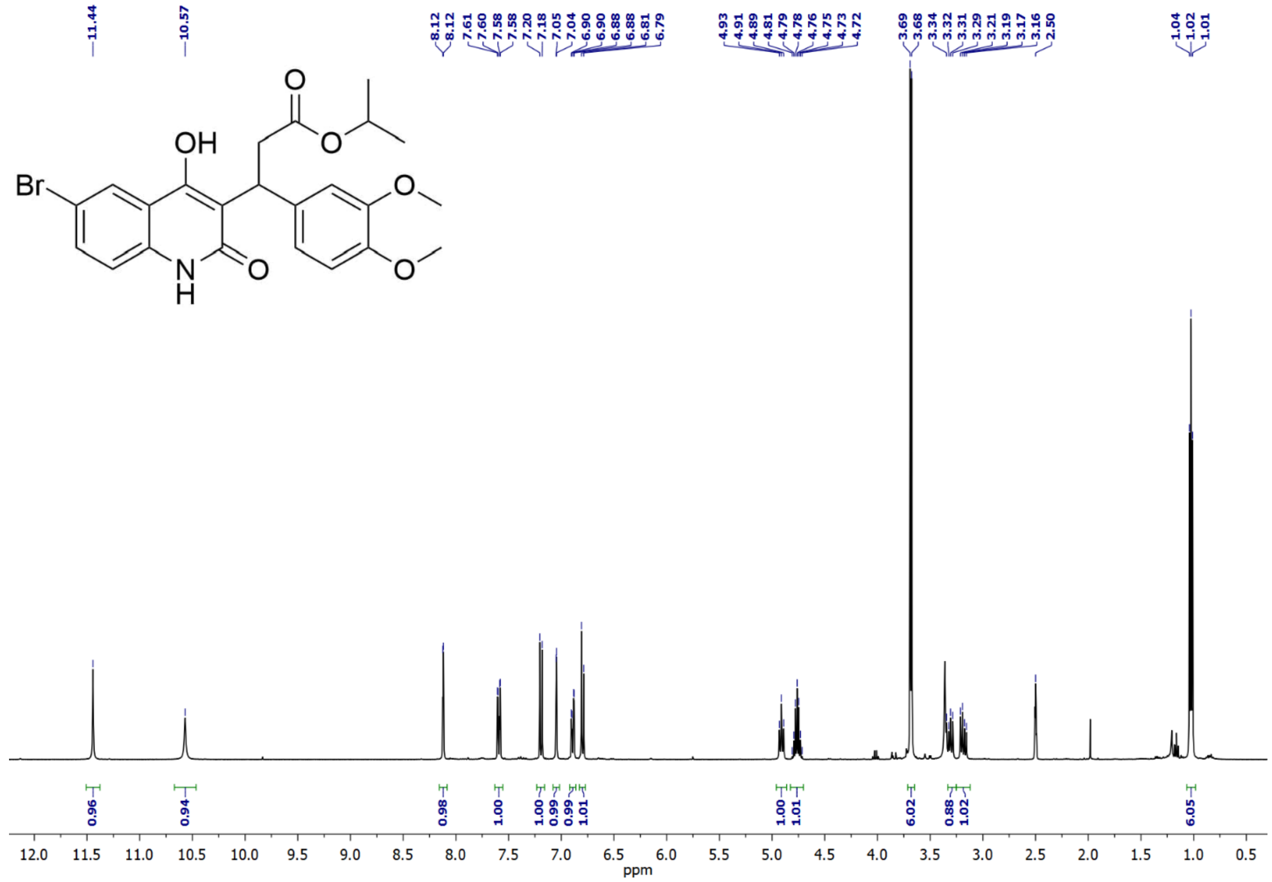

^{13}C NMR spectrum of compound 12c (100 MHz, DMSO-d_6)

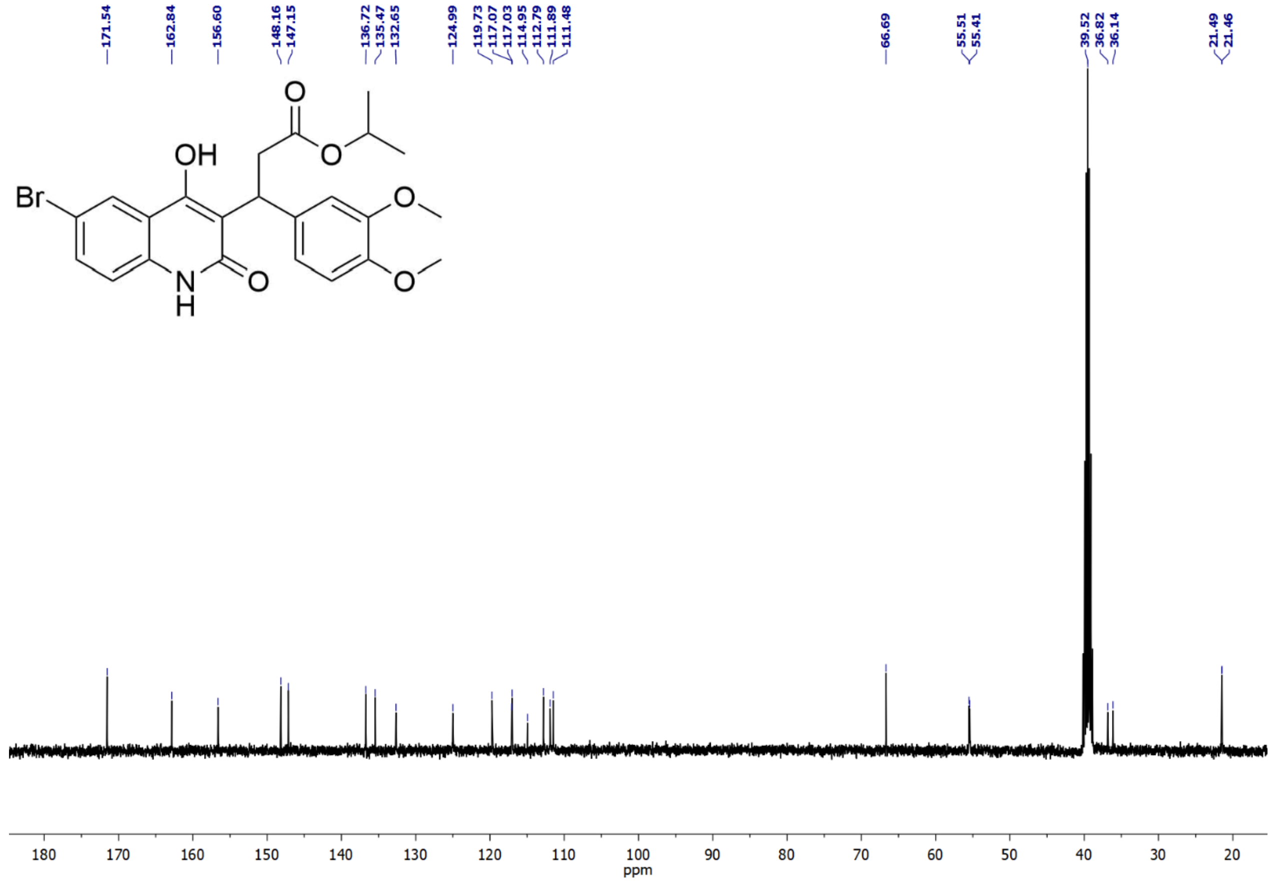

^{1}H NMR spectrum of compound 13a (400 MHz, DMSO-d_6)

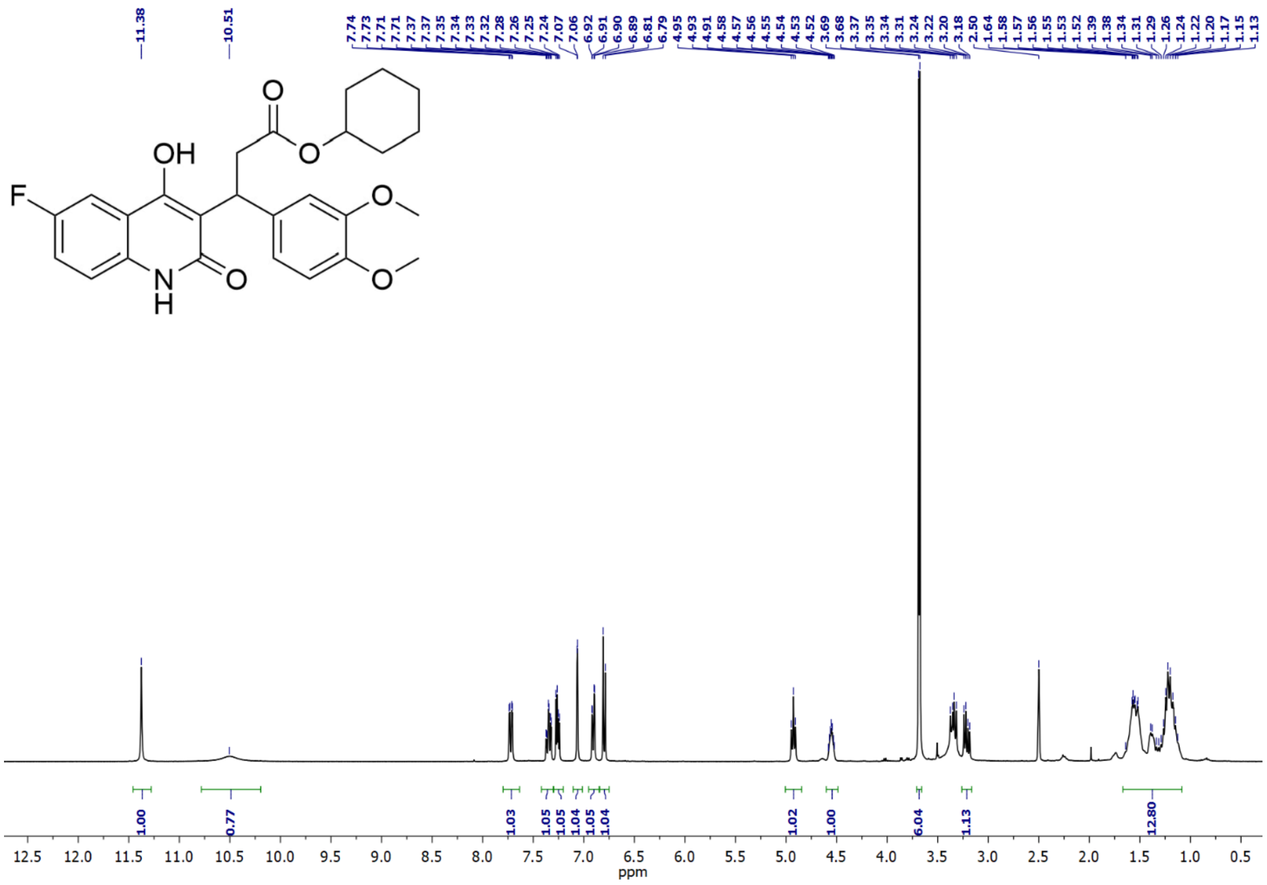

^{13}C NMR spectrum of compound 13a (100 MHz, DMSO-d_6)

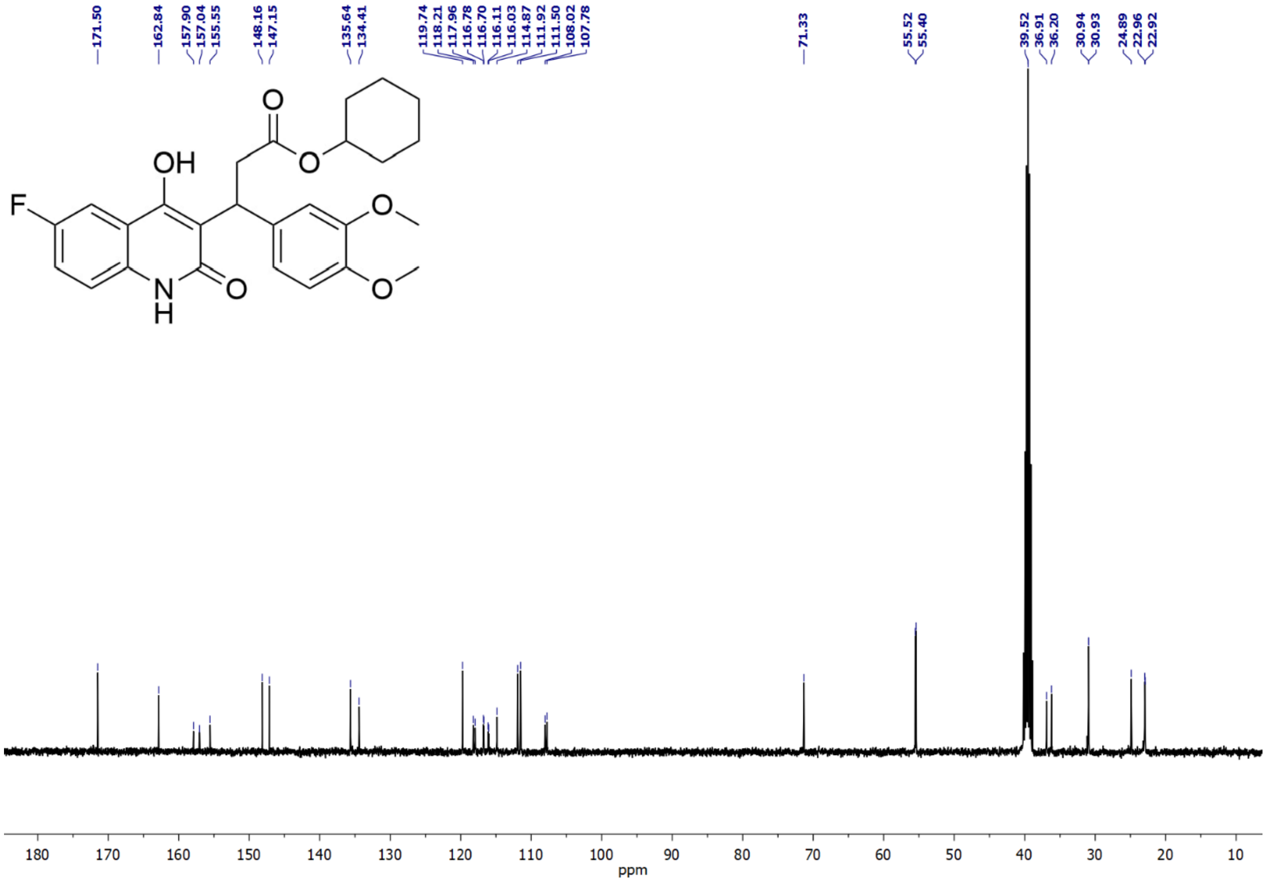

^{1}H NMR spectrum of compound 13b (400 MHz, DMSO-d_6)

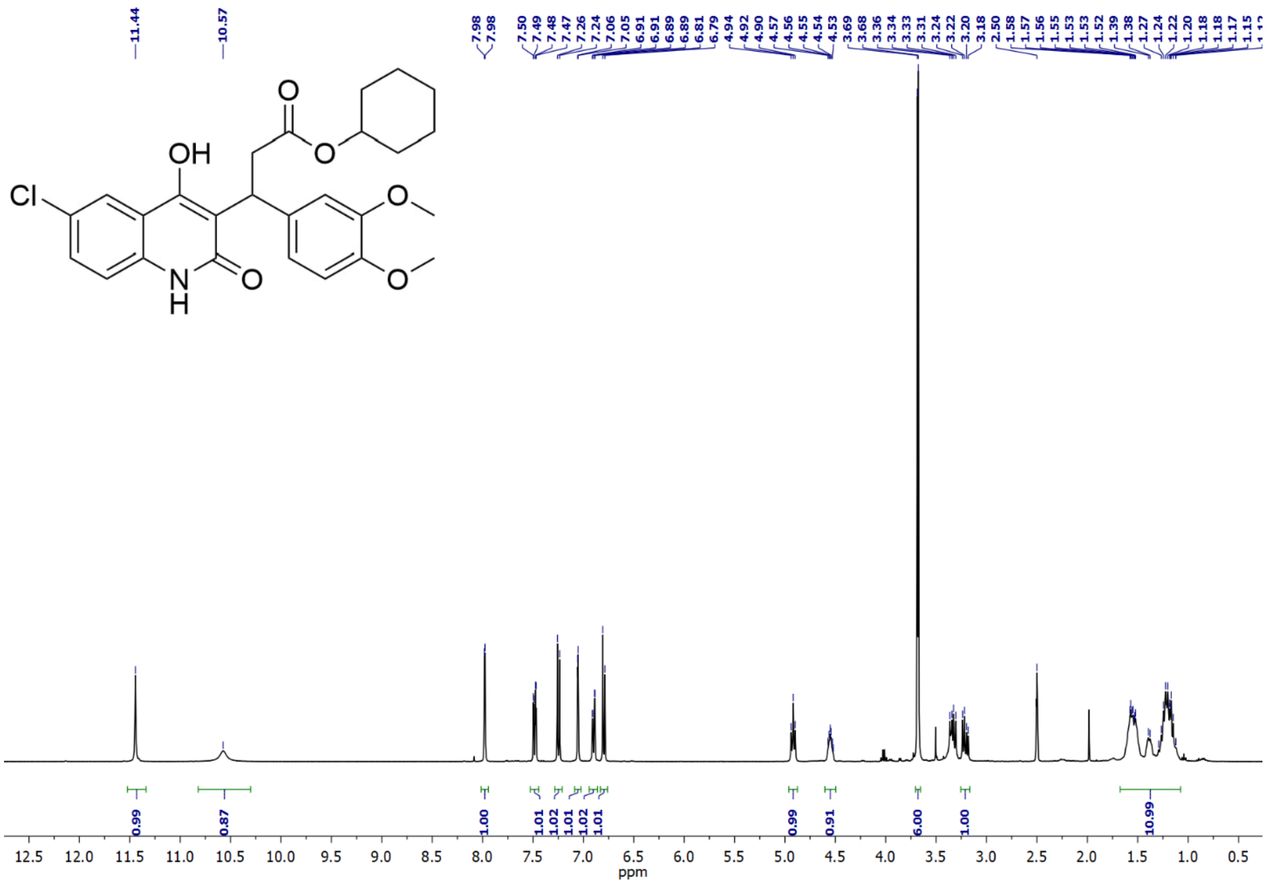

^{13}C NMR spectrum of compound 13b (100 MHz, DMSO-d_6)

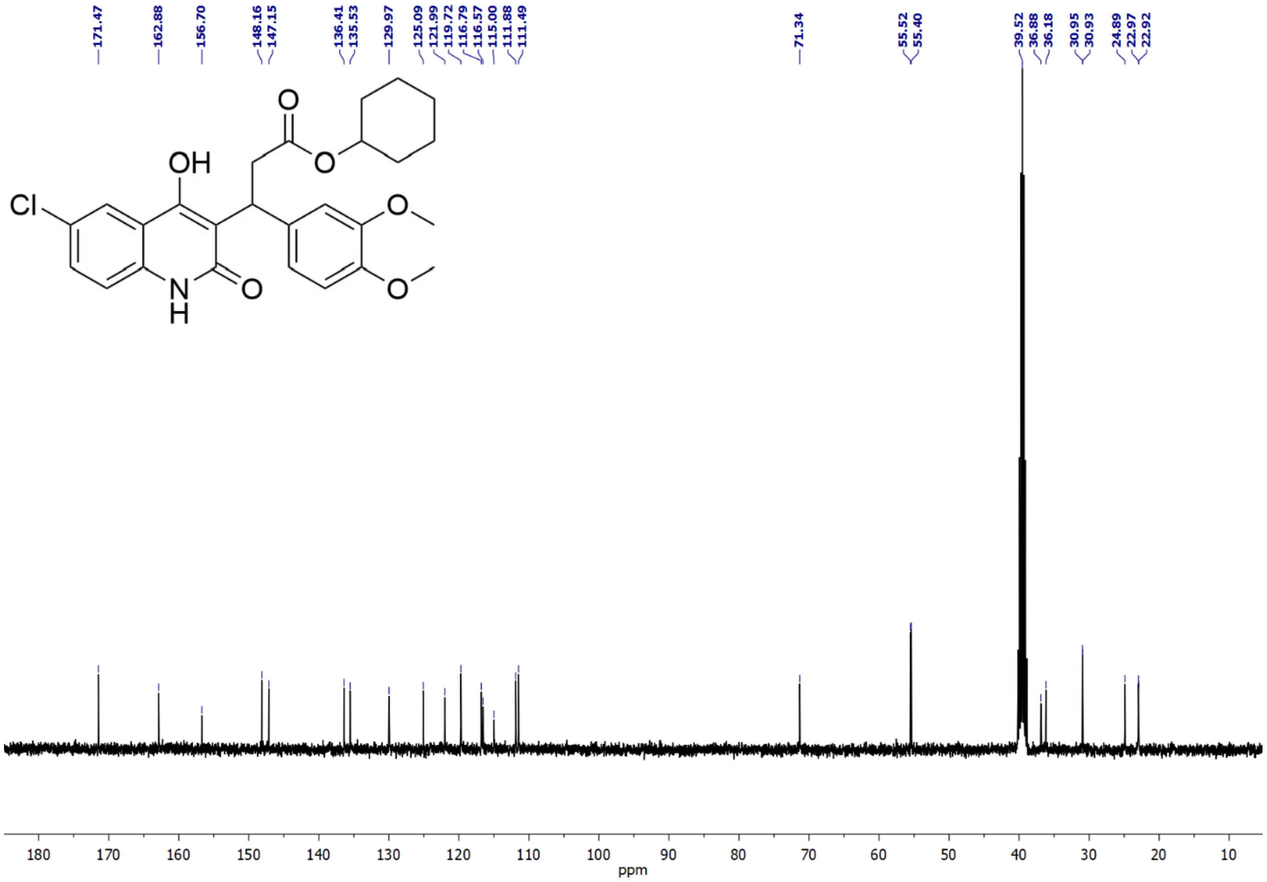

^{1}H NMR spectrum of compound 13c (400 MHz, DMSO-d_6)

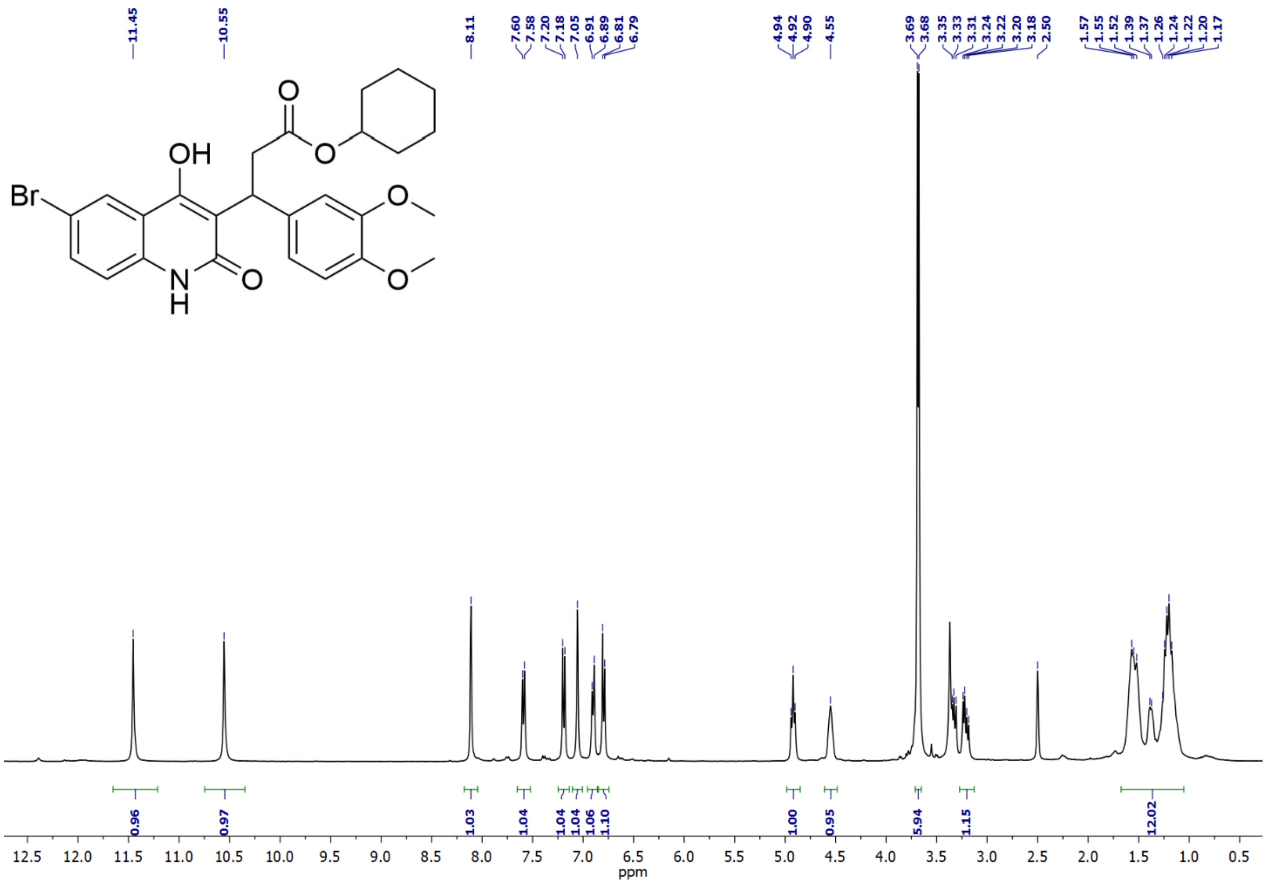

^{13}C NMR spectrum of compound 13c (100 MHz, DMSO-d_6)

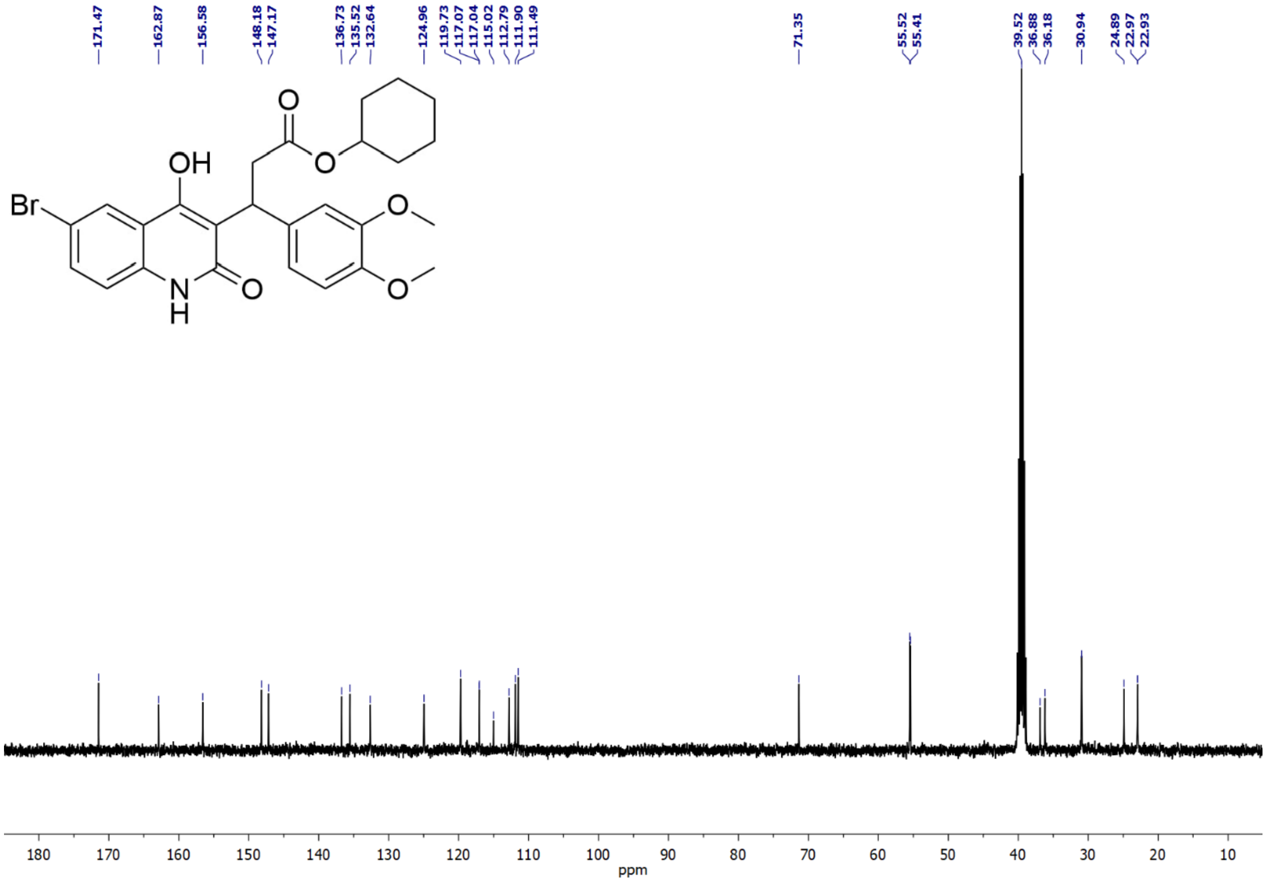

^{1}H NMR spectrum of compound 14a (400 MHz, DMSO-d_6)

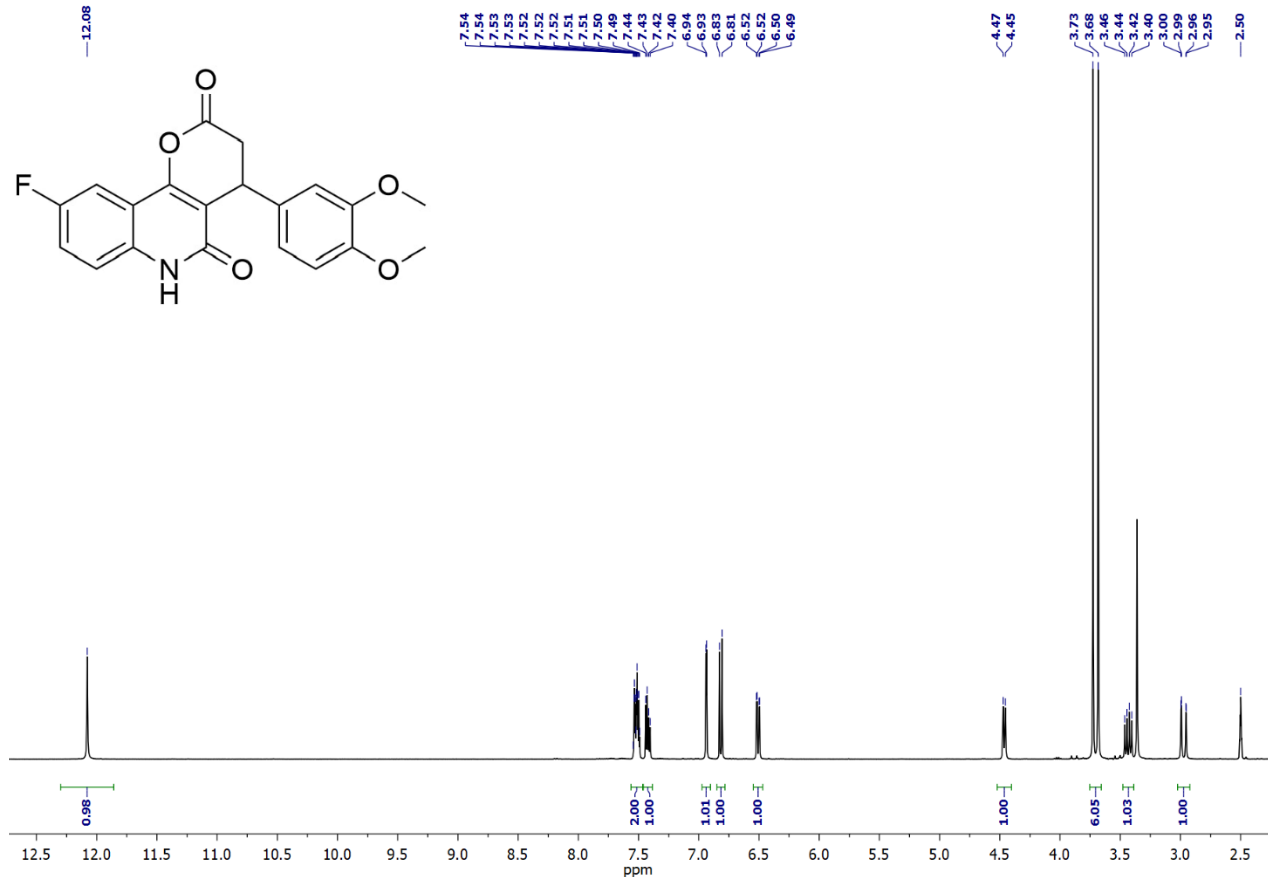

^{13}C NMR spectrum of compound 14a (100 MHz, DMSO-d_6)

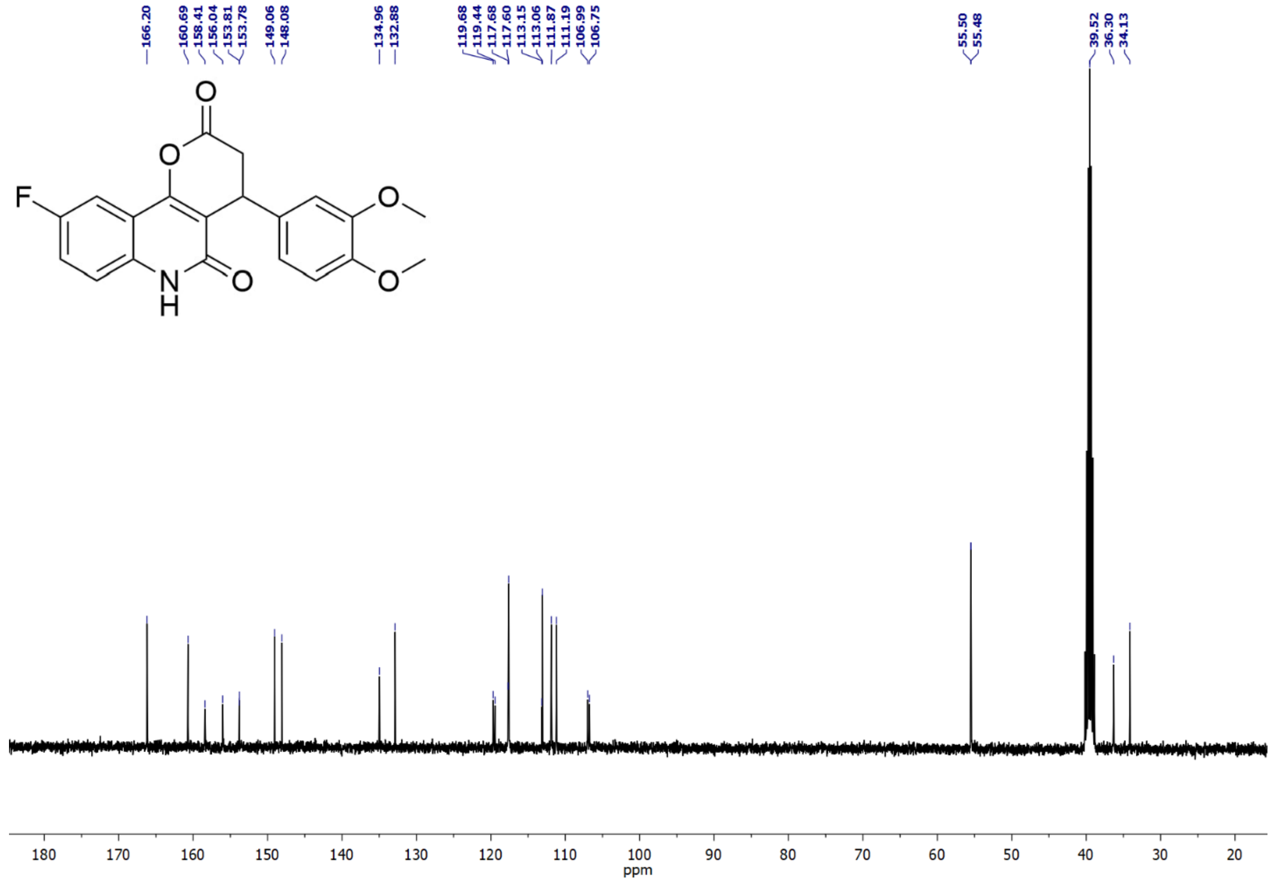

^{1}H NMR spectrum of compound 14b (400 MHz, DMSO-d_6)

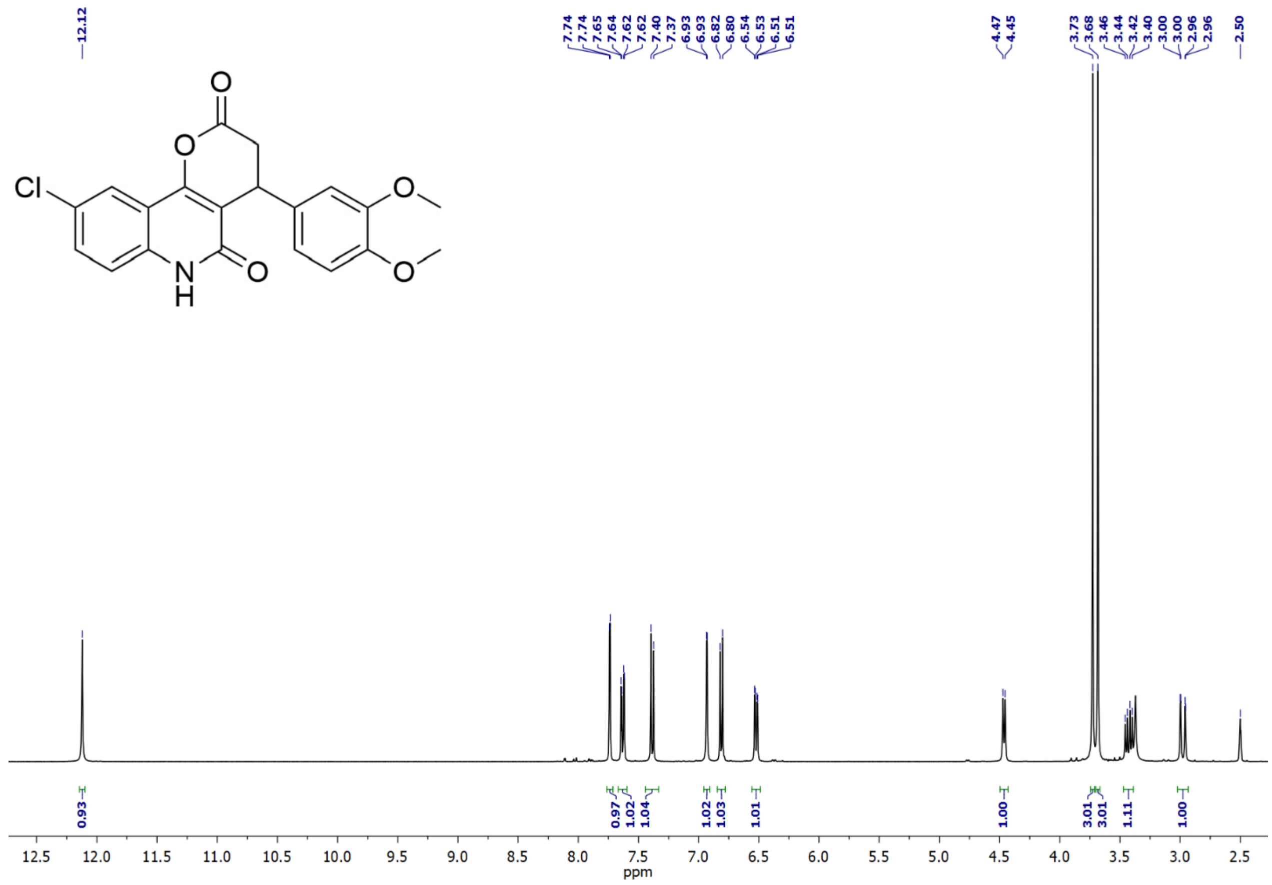

^{13}C NMR spectrum of compound 14b (100 MHz, DMSO-d_6)

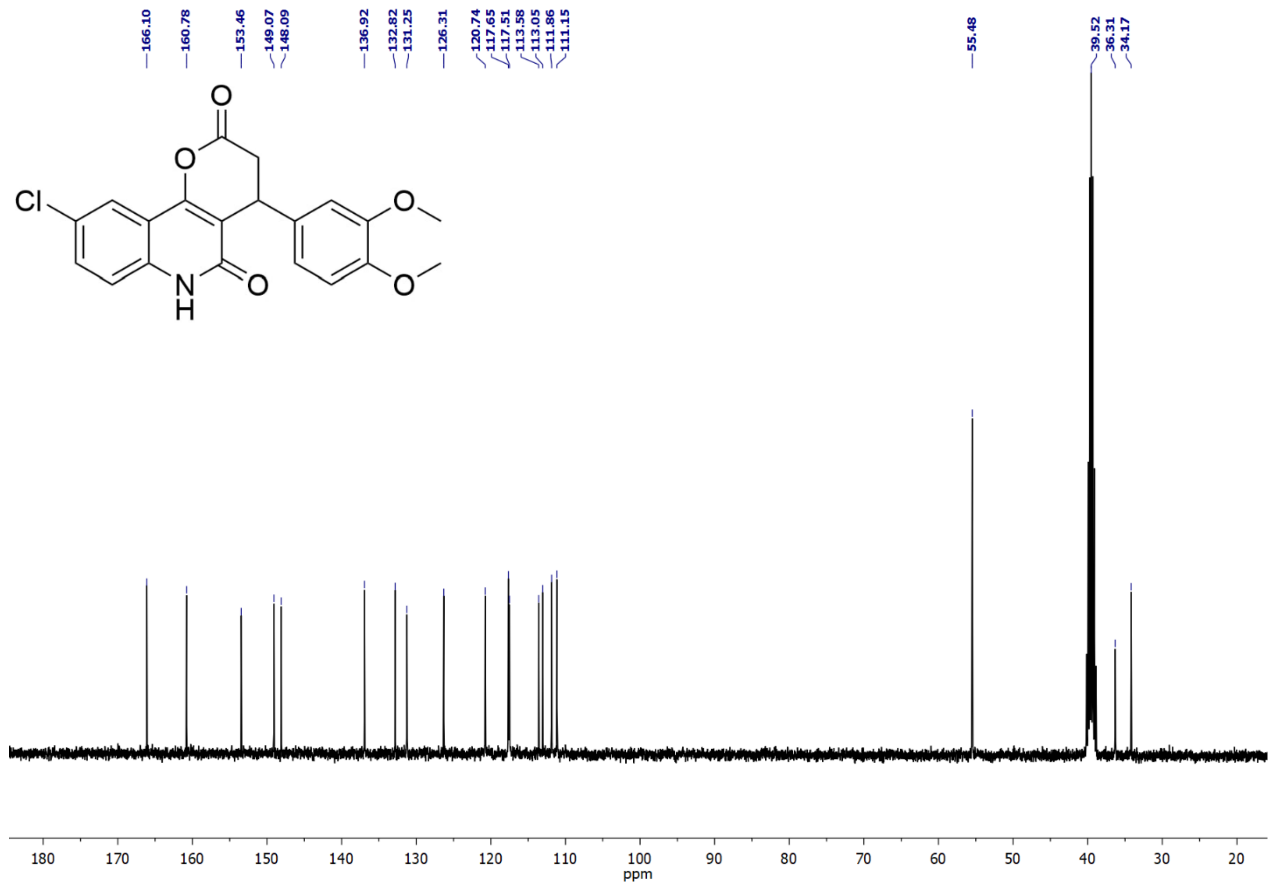

^{1}H NMR spectrum of compound 14c (400 MHz, DMSO-d_6)

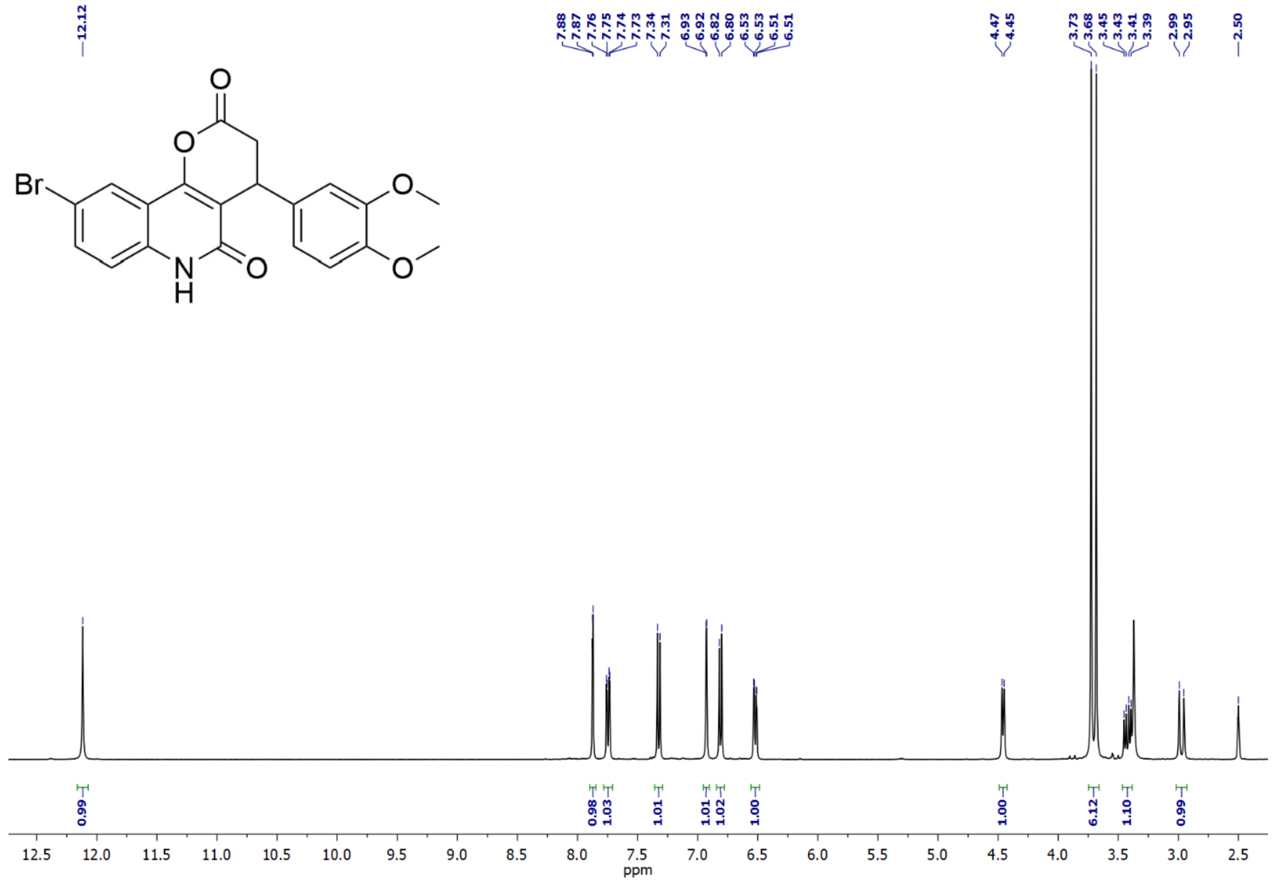

^{13}C NMR spectrum of compound 14c (100 MHz, DMSO-d_6)

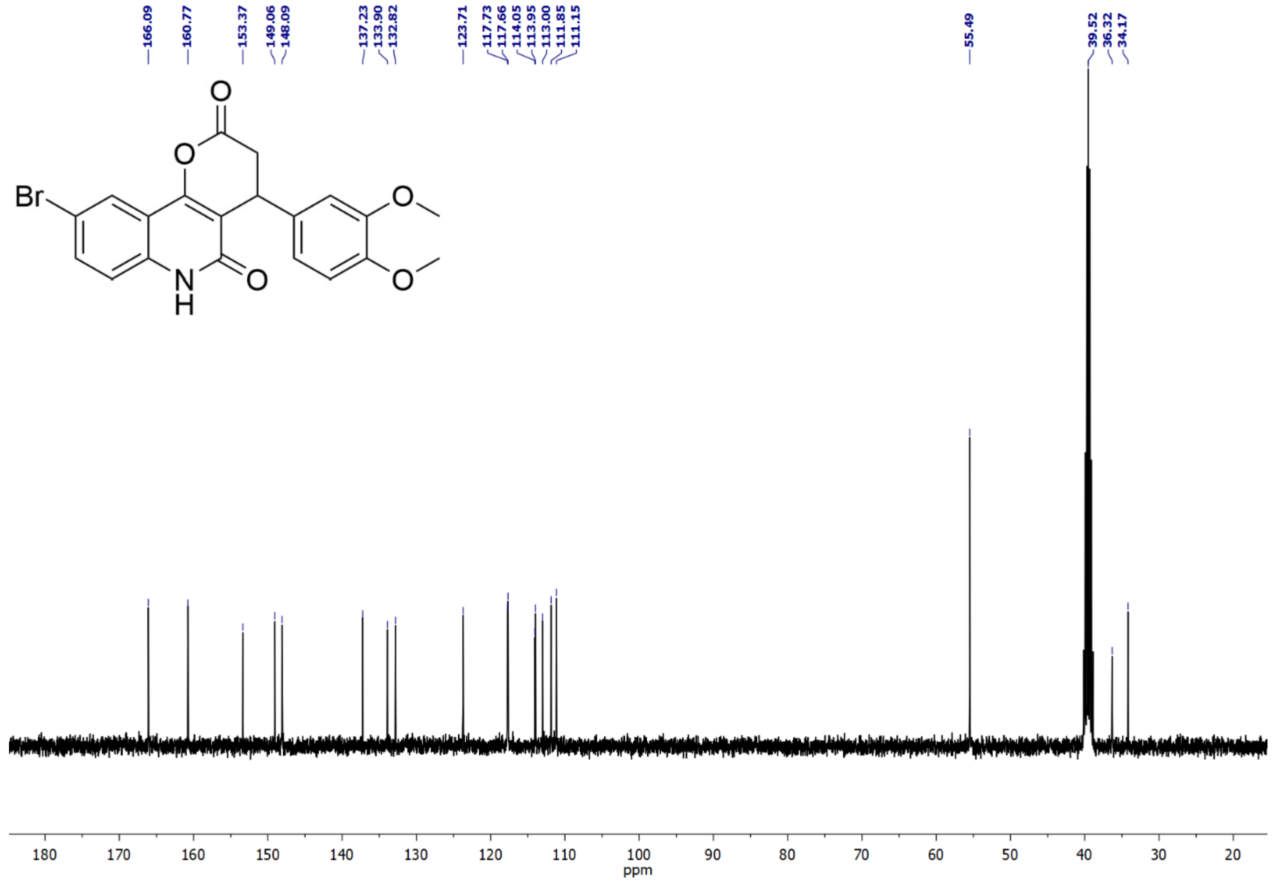

Equilibrium geometry configurations of considered molecules

Pyranoquinoline

C	-3.12234909712439	-0.03597787011098	0.82929143345980
C	-2.32665939033558	-0.67591660369805	-0.13601440299578
C	-2.73644773530807	-1.90938398603897	-0.67458888409810
C	-3.95065877356861	-2.47983150422652	-0.25555624473732
C	-4.71246452389567	-1.84223753413677	0.71086287073097
C	-4.30028731953168	-0.61912886436659	1.26180527168545
N	-1.92653433122437	-2.53316061784343	-1.60155126565900
C	-0.69163302129714	-2.08590750113030	-2.03834774619812
C	-0.28384557487996	-0.78245025108482	-1.50417032736463
C	-1.07570366084122	-0.12895104976696	-0.61956729967750
O	-0.75022765207616	1.08729576473109	-0.09976493580011
C	0.23794321516040	1.86787975528949	-0.64872796092273
C	0.84436792294024	1.34938046412110	-1.92928277521322
C	1.01424345261996	-0.17124604853026	-1.96886134462365
C	2.20938279873964	-0.71133849523336	-1.18974646491132
C	2.78686082918964	-1.92005512355675	-1.59795279200675
C	3.87134098918010	-2.45931262777215	-0.91141846197250
C	4.39170213466338	-1.80525880929193	0.20568500118931
C	3.82561931399891	-0.60225237681825	0.61975002298427
C	2.74259098831314	-0.05839996575549	-0.07350473687850
O	0.50974437453431	2.89303472553912	-0.10540340319930
O	-0.02844010077138	-2.74407897008965	-2.82073919864005
H	-2.78217971723920	0.91903329730790	1.23356826126805
H	-4.28601168121206	-3.42156649625046	-0.69941583750138
H	-5.64114280458202	-2.30483832256516	1.05506178495039
H	-4.91271766163661	-0.12330637354537	2.01717016378991
H	-2.21270213947629	-3.42580038687508	-1.98998460220320
H	0.14587823233689	1.64541359346804	-2.73128758455984
H	1.78360453430401	1.89124760695589	-2.09760057023562
H	1.15271249016580	-0.47717092873983	-3.01601713784615
H	2.35611816042070	-2.44489600934652	-2.45392574314998
H	4.31821410862522	-3.39437729835506	-1.26027880945676
H	5.23583928378470	-2.23326578268837	0.75220628107971
H	4.23691420256797	-0.07042457223433	1.48139619422916
H	2.32892815345539	0.89224916263886	0.27291124448455

Open-chain methyl ether

C	-3.72809313853103	-0.66966680604222	-1.53405548187202
C	-2.54768400471733	-0.74722046103160	-0.77220359944046
C	-2.45307835399952	-1.70970145806925	0.25220338231691
C	-3.51930916960337	-2.59809248206003	0.48528153470669
C	-4.66402620550741	-2.51606445416229	-0.28956788232474

C	-4.77616993846146	-1.54502081802302	-1.29914783281963
N	-1.30308356856960	-1.76282108624048	1.00454965126446
C	-0.17413049604411	-0.98207172741155	0.81449028424909
C	-0.21813637422972	-0.06626950687309	-0.33210418875517
C	-1.37858351611328	0.08133084372624	-1.03852749943121
O	3.19983754481692	2.18683688102090	-1.49834652224605
C	3.04643798450718	2.12050852877360	-0.16649060175775
C	1.73597905550184	1.48474434921487	0.24936425009020
C	1.10537526631233	0.53871907416426	-0.77767938115999
C	1.99495043082750	-0.61885190378572	-1.23054411597742
C	1.60572935916802	-1.33937319292000	-2.36645709059732
C	2.33440588123526	-2.44188010317057	-2.80170968933900
C	3.47782163092966	-2.83816668680289	-2.10576652623894
C	3.87195940777369	-2.12881028684442	-0.97312256055556
C	3.13323655010439	-1.02770315178931	-0.53501891894321
O	3.87431938659574	2.52938629125590	0.60407116874762
O	0.77695749868136	-1.09189603978583	1.56989700501044
H	-3.79831897759595	0.08549508040323	-2.31850759520613
H	-3.43440944119750	-3.34551080681582	1.27801360538328
H	-5.48680993101787	-3.20998274546737	-0.09831460506084
H	-5.68875110929449	-1.47358953305099	-1.89449196245932
H	-1.23912476520146	-2.41313576294445	1.78008518291114
H	1.91655214919173	0.96984655808087	1.20124094608278
H	1.03037305034671	2.30215524170700	0.47638535878607
H	0.94912034241881	1.14150869025320	-1.69113287519908
H	0.70479142292192	-1.03602881220360	-2.91007103251092
H	2.00235899846214	-2.99856560648623	-3.68174288043933
H	4.05705638822688	-3.69951120796833	-2.44732074425534
H	4.75329714108313	-2.44486654790373	-0.40941479740489
H	3.44256723476428	-0.50409785068238	0.37230427337025
O	-1.51800612733025	0.93743463559972	-2.06614994309733
C	4.47413218766982	2.61725563309237	-1.95644040352845
H	-0.69295600816120	1.41925676475131	-2.19246611228985
H	4.48638181639826	2.45183738431854	-3.04018845337409
H	5.26729262139152	2.02889364283271	-1.47548093793404
H	4.64173777624641	3.68068943934048	-1.72942240870080

In vitro antibacterial activity studies

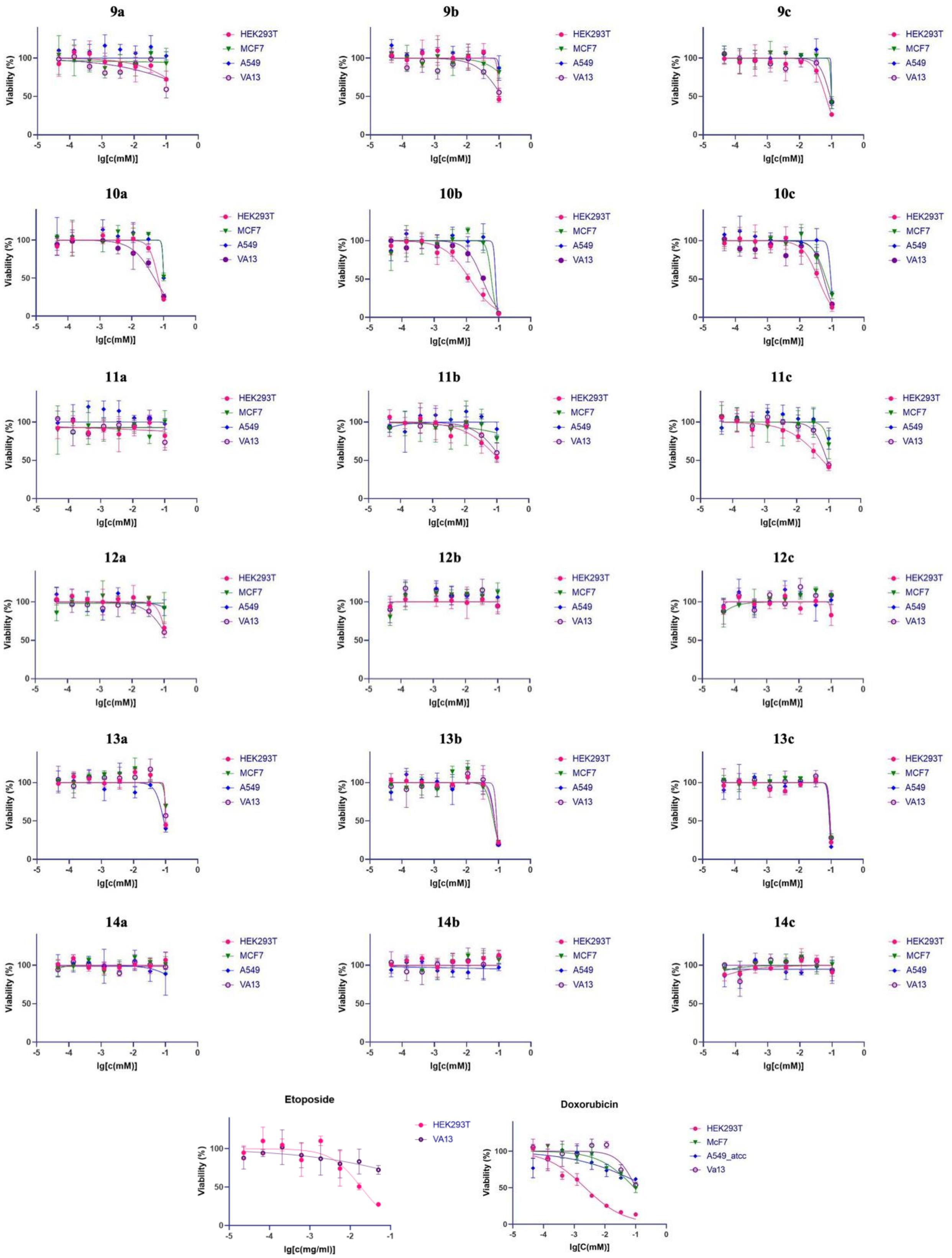

Minimum inhibitory concentration evaluation

Activity was evaluated by determining the minimum inhibitory concentration (MIC) values using the microdilution method in Mueller–Hinton Broth (Beckton and Dickinson, Le Pont-de-Claix,

France), in accordance with the standard procedure for assessing the antimicrobial susceptibility of microorganisms [16]. The analysis was conducted in 96-well microtiter plates (Medpolymer, Saint Petersburg, Russia). The bacterial inoculum was introduced within 15 min of preparation. MIC values were determined after incubation for 15–18 h at (36 ± 1) °C. Microbial growth in the presence of the tested compounds was compared to the growth control (without exposure to the test samples). The MIC was defined as the lowest concentration at which visible microbial growth was inhibited.

Table S1: Results of the study of antibacterial activity of compounds **9–14**.

Minimum inhibitory concentration	K12	Δtolc	lptd	Dimension	c_{max} (μmol/L)
Compound **10a**	$>c_{max}$	$>c_{max}$	$>c_{max}$	μmol/L	400
Compound **10b**	$>c_{max}$	$>c_{max}$	$>c_{max}$	μmol/L	400
Compound **10c**	$>c_{max}$	$>c_{max}$	$>c_{max}$	μmol/L	400
Compound **9a**	$>c_{max}$	$>c_{max}$	$>c_{max}$	μmol/L	400
Compound **9b**	$>c_{max}$	$>c_{max}$	$>c_{max}$	μmol/L	400
Compound **9c**	$>c_{max}$	400	$>c_{max}$	μmol/L	400
Compound **11a**	$>c_{max}$	$>c_{max}$	$>c_{max}$	μmol/L	400
Compound **11b**	$>c_{max}$	$>c_{max}$	$>c_{max}$	μmol/L	400
Compound **11c**	$>c_{max}$	$>c_{max}$	$>c_{max}$	μmol/L	400
Compound **12a**	$>c_{max}$	400	$>c_{max}$	μmol/L	400
Compound **12b**	$>c_{max}$	$>c_{max}$	$>c_{max}$	μmol/L	400
Compound **12c**	$>c_{max}$	$>c_{max}$	$>c_{max}$	μmol/L	400
Compound **13a**	$>c_{max}$	400	$>c_{max}$	μmol/L	400
Compound **13b**	$>c_{max}$	$>c_{max}$	$>c_{max}$	μmol/L	400
Compound **13c**	$>c_{max}$	$>c_{max}$	$>c_{max}$	μmol/L	400
Compound **14a**	$>c_{max}$	$>c_{max}$	$>c_{max}$	μmol/L	400
Compound **14b**	$>c_{max}$	$>c_{max}$	$>c_{max}$	μmol/L	400
Compound **14c**	$>c_{max}$	$>c_{max}$	$>c_{max}$	μmol/L	400

References

1. Mardirossian, N.; Head-Gordon, M. *J. Chem. Phys.* **2015**, *142*, 074111. doi: 10.1063/1.4907719

2. Grimme, S.; Ehrlich, S.; Goerigk, L. *J. Comput. Chem.* **2011**, *32* (7), 1456–1465. doi: 10.1002/jcc.21759

3. Neese, F.; Wennmohs, F.; Becker, U.; Riplinger, C. *J. Chem. Phys.* **2020**, *152*, 224103. doi: 10.1063/5.0004608

4. Lu, T.; Chen, F. *J. Comput. Chem.* **2012**, *33* (5), 580–592. doi: 10.1002/jcc.22885

5. Oláh, J.; Van Alsenoy, C.; Sannigrahi, A. B. *J. Phys. Chem. A* **2002**, *106*, 3885–3890. doi: 10.1021/jp014039h

6. Roy, R. K.; Krishnamurti, S.; Geerlings, P.; Pal, S. *J. Phys. Chem. A* **1998**, *102* (21), 3746–3755. doi: 10.1021/jp973450v

7. Mosmann, T. *J. Immunol. Methods* **1983**, *65* (1–2), 55–63. doi: 10.1016/0022-1759(83)90303-4

8. Ferretti, M. D.; Neto, A. T.; Morel, A. F.; Kaufman, T. S.; Larghi, E. L. *Eur. J. Med. Chem.* **2014**, *81*, 253–266. doi: 10.1016/j.ejmech.2014.05.024

9. Chauhan, J.; Yoshioka, M.; Pogash, S.; Strovel, J. W.; Fletcher, S. *Bioorg. Med. Chem. Lett.* **2023**, *86*, 129220. doi: 10.1016/j.bmcl.2023.129220

10. Gao, W. T.; Hou, W. D.; Zheng, M. R.; Tang, L. J. *Synth. Commun.* **2010**, *40* (5), 732–738. doi: 10.1080/00397910903013713

11. Stubba, D.; Lahm, G.; Geffe, M.; Runyon, J. W.; Arduengo, A. J.; Opatz, T. *Angew. Chem. Int. Ed.* **2015**, *54* (47), 14187–14189. doi: 10.1002/anie.201508500

12. Napoly, F.; Jean-Gérard, L.; Goux-Henry, C.; Draye, M.; Andrioletti, B. *Eur. J. Org. Chem.* **2014**, *2014* (4), 781–787. doi: 10.1002/ejoc.201301093

13. Fernandes, R. A.; Kumar, P.; Bhowmik, A.; Gorve, D. A. *Org. Lett.* **2022**, *24* (19), 3435–3439. doi: 10.1021/acs.orglett.2c00884

14. Gu, J. X.; Holland, H. L. *Synth. Commun.* **1998**, *28* (18), 3305–3315. doi: 10.1080/00397919808004439

15. Zhou, L. S.; Yang, K. W.; Feng, L.; Xiao, J. M.; Liu, C. C.; Zhang, Y. L.; Crowder, M. W. *Bioorg. Med. Chem. Lett.* **2013**, *23* (4), 949–954. doi: 10.1016/j.bmcl.2012.12.051

16. Cockerill, F. R.; Wikler, M. A.; Alder, J.; Dudley, M. N.; Eliopoulos, G. M.; Ferraro, M. J.; Hardy, D. J.; Hecht, D. W.; Hindler, J. A.; Patel, J. B.; Powell, M.; Swenson, J. M.; Thomson, R. B.; Traczewski, M. M.; Turnbrighe, J. D.; Weinstein, M. P.; Zimmer, B. L. *Methods for Dilution Antimicrobial Susceptibility Tests for Bacteria That Grow Aerobically*; National Committee for Clinical Laboratory Standards: Annapolis Junction, MD, USA, **2012**; Volume 32, ISBN 1562387839
